# Detailed Inspection of *γ*-ray, Fast and Thermal Neutrons Shielding Competence of Calcium Oxide or Strontium Oxide Comprising Bismuth Borate Glasses

**DOI:** 10.3390/ma14092265

**Published:** 2021-04-27

**Authors:** Gandham Lakshminarayana, Youssef Elmahroug, Ashok Kumar, Huseyin Ozan Tekin, Najeh Rekik, Mengge Dong, Dong-Eun Lee, Jonghun Yoon, Taejoon Park

**Affiliations:** 1 Intelligent Construction Automation Center, Kyungpook National University, 80 Daehak-ro, Buk-gu, Daegu 41566, Korea; 2 Unité de Recherche de Physique Nucléaire et des Hautes Energies, Faculté des Sciences de Tunis, Université de Tunis El Manar, Tunis 2092, Tunisia; youssef_phy@hotmail.fr; 3 Ecole Centrale Polytechnique Privée de Tunis, Univesité Centrale, La Goulette 2015, Tunisia; 4 Department of Physics, University College, Benra-Dhuri, Punjab 148024, India; ajindal9999@gmail.com; 5 Medical Diagnostic Imaging Department, College of Health Sciences, University of Sharjah, Sharjah 27272, United Arab Emirates; tekin765@gmail.com; 6 Medical Radiation Research Center (USMERA), Uskudar University, Istanbul 34672, Turkey; 7 Physics Department, Faculty of Science, University of Ha’il, Ha’il 81451, Saudi Arabia; na.rekik@uoh.edu.sa; 8 Department of Chemistry, University of Alberta, Edmonton, AB T6G 2G2, Canada; 9 Department of Resource and Environment, Northeastern University, Shenyang 110819, China; mg_dong@163.com; 10 School of Architecture, Civil, Environment and Energy, Kyungpook National University, 1370 Sangyeok-dong, Buk-gu, DaeGu 702-701, Korea; 11 Department of Mechanical Engineering, Hanyang University, 55 Hanyangdaehak-ro, Ansan 15588, Gyeonggi-do, Korea; 12 Department of Robotics Engineering, Hanyang University, 55 Hanyangdaehak-ro, Ansan 15588, Gyeonggi-do, Korea

**Keywords:** B_2_O_3_-Bi_2_O_3_-CaO glass, B_2_O_3_-Bi_2_O_3_-SrO glass, *γ*- and neutron radiation, Phy-X/PSD software, PENELOPE code, radiation protection efficiency

## Abstract

For both the B_2_O_3_-Bi_2_O_3_-CaO and B_2_O_3_-Bi_2_O_3_-SrO glass systems, *γ*-ray and neutron attenuation qualities were evaluated. Utilizing the Phy-X/PSD program, within the 0.015–15 MeV energy range, linear attenuation coefficients (µ) and mass attenuation coefficients (μ/ρ) were calculated, and the attained μ/ρ quantities match well with respective simulation results computed by MCNPX, Geant4, and Penelope codes. Instead of B_2_O_3_/CaO or B_2_O_3_/SrO, the Bi_2_O_3_ addition causes improved *γ*-ray shielding competence, i.e., rise in effective atomic number (*Z_eff_*) and a fall in half-value layer (HVL), tenth-value layer (TVL), and mean free path (MFP). Exposure buildup factors (EBFs) and energy absorption buildup factors (EABFs) were derived using a geometric progression (G–P) fitting approach at 1–40 mfp penetration depths (PDs), within the 0.015–15 MeV range. Computed radiation protection efficiency (RPE) values confirm their excellent capacity for lower energy photons shielding. Comparably greater density (7.59 g/cm^3^), larger μ, μ/ρ, *Z_eff_*, equivalent atomic number (*Z_eq_*), and RPE, with the lowest HVL, TVL, MFP, EBFs, and EABFs derived for 30B_2_O_3_-60Bi_2_O_3_-10SrO (mol%) glass suggest it as an excellent *γ*-ray attenuator. Additionally, 30B_2_O_3_-60Bi_2_O_3_-10SrO (mol%) glass holds a commensurably bigger macroscopic removal cross-section for fast neutrons (*Σ_R_*) (=0.1199 cm^−1^), obtained by applying Phy-X/PSD for fast neutrons shielding, owing to the presence of larger wt% of ‘Bi’ (80.6813 wt%) and moderate ‘B’ (2.0869 wt%) elements in it. 70B_2_O_3_-5Bi_2_O_3_-25CaO (mol%) sample (B: 17.5887 wt%, Bi: 24.2855 wt%, Ca: 11.6436 wt%, and O: 46.4821 wt%) shows high potentiality for thermal or slow neutrons and intermediate energy neutrons capture or absorption due to comprised high wt% of ‘B’ element in it.

## 1. Introduction

Nowadays, utilization and generation of radiation are eminent in distinct technological applications, such as nuclear fission reactors for clean energy (e.g., ^235^U or ^239^Pu fissile isotopes’ usage), therapeutic nuclear medicine (radiopharmaceuticals, e.g., ^137^Cs, ^60^Co, ^99m^Tc, and ^123^I radioisotopes handling for disease (oncology) diagnosis and treatment, single-photon emission computed tomography (SPECT)—body tissues and organs imaging), and outer space research. In these fields, to assure safety and protection of radiation workers, nuclear medicine staff members, and astronauts from deleterious effects of undesired radiation (e.g., *γ*-rays, neutrons, *β*-particles, in space—high energy electrons, protons, and heavy ions, etc.) exposure, appropriate shielding materials are compulsory. For instance, as a fission product of ^235^U, ^137^Cs radioactive isotope, which emits high energy *β*-particles and *γ*-rays (charge = 0, rest mass = 0), highly contaminates the surrounding environment (water, soil, air) once any nuclear reactor accident occurs (e.g., Fukushima Daiichi Nuclear Power Plant (FDNPP) accident, Japan, 2011) [1]. In humans, external exposure to ^137^Cs (in greater amounts) can cause radiation burns, acute radiation sickness (ARS), coma, and even death, while internal exposure through inhalation/ingestion increases cancer (abnormal growth in cells) risk. Likewise, neutrons (originated as a product of nuclear fission and radioactive decay, mass and charge = 939.57 MeV and 0) can travel larger distances in air, and, owing to their remarkable ability to penetrate other materials unlike *α*-particles, they are harmful to humans’ soft tissues in organs when interacting with the body (which consists mostly of water). They directly interact with the atomic nuclei of living cells, causing ionization among nearby atoms. Generally, within the nucleus, neutron interaction greatly relies on incident energy and nucleus movement. The International Atomic Energy Agency (IAEA) [2] and International Commission on Radiological Protection (ICRP) [3] are the principal organizations that promote radiation safety and safeguard and set standards concerning radiation exposure limits for working personnel and the general public, apart from International Radiation Protection Association (IRPA) defined ALARA (As Low as Reasonably Achievable) principle. It is essential to limit radiation exposure by following the nuclear regulatory instructions at nuclear energy facilities and radiotherapy centers.

Customarily, concrete, owing to its favorable chemical composition (holds both light and heavy nuclei), low fabrication cost, ease of construction, and superior *γ*-rays and neutron attenuation ability, has been used for nuclear radiation shielding objectives. Nevertheless, concrete is known for some demerits, such as loss of moisture and consequent cracking due to radiation heat, poor mechanical features upon exposure to high energy *γ*-rays multiple scattering over time, elastic modulus and compressive and tensile strength degradation by neutron irradiation, bigger space occupation, opacity, and immovability [4,5]. An alternative to concrete, lead (Pb) and Pb-containing materials (manufactured in various shapes—slabs, plates, and sheets, etc.) possess excellent *X*-ray and *γ*-ray attenuation capacity, but ‘Pb’ has disadvantages, such as low melting point (600.6 K), and is detrimental to human health and the surrounding environment [6,7]. For these reasons, in recent times, different research groups have actively focused their efforts on finding suitable replacement materials for concrete and ‘Pb’, e.g., glasses, which demonstrate encouraging characteristics, such as low cost, medium to high density (*ρ*), structural stability with prolonged irradiation, high mechanical and thermal strength, better optical (visible light) transparency, nontoxicity (100% recyclable), and environmental safety [8,9,10,11,12,13,14,15].

B_2_O_3_ (B (*Z* = 5)) glasses possess low manufacturing cost compared to TeO_2_ and GeO_2_ glasses, lower melting points than SiO_2_ glasses, adequate optical transparency, good thermal stability, and ample glass formation tendency when B_2_O_3_ has high dissociation energy (=356 kcal/mol) and large single B–O bond strengths—498 kJ/mol (‘B’ CN (coordination number) = 3) and 373 kJ/mol (‘B’ CN = 4), accordingly [16]. Pure B_2_O_3_ glass (holds planer BO_3_ and B_3_O_3_ boroxol ring structural units (‘B’ CN = 3) [17]) has large phonon energy (~1300–1500 cm^−1^) and high hygroscopicity. Bi_2_O_3_ (Bi (*Z* = 83)), Bi^3+^ cation—low field strength and huge polarizability), a heavy metal oxide, plays a network forming or modifying role when included in the glass composition (e.g., B_2_O_3_), forming [BiO_3_] pyramidal units or [BiO_6_] octahedral units in glass structure, contingent upon its high or low content [18]. Moreover, glasses with high Bi_2_O_3_ (*ρ* = 8.9 g/cm^3^) content exhibit high ‘*ρ*’, high refractive index (>2), and large third-order nonlinear optical susceptibility (*χ*^(3)^, about 10^−11^ esu), apart from good chemical, thermal, and mechanical stabilities [19]. In addition, when added, alkaline earth oxides, such as CaO and SrO modify (breaks the B–O bonds) the B_2_O_3_ glass network structure, converting ‘B’ CN from 3→4 by forming nonbridging oxygens, and also enhance the glass-forming regions [20,21].

It is vital to explore definite parameters, such as linear attenuation coefficient (*μ*), mass attenuation coefficient (μ/ρ), effective atomic number (*Z_eff_*), effective electron density (*N_eff_*), half-value layer (HVL), tenth-value layer (TVL), mean free path (MFP), radiation protection efficiency (RPE), equivalent atomic number (*Z_eq_*), exposure buildup factor (EBF), and energy absorption buildup factor (EABF) for *γ*-rays and other quantities, such as macroscopic effective removal cross-sections for fast neutrons (*Σ_R_*), scattering cross-sections (coherent (*σ_cs_*) and incoherent (σ_ics_)), and absorption cross-section (*σ_A_*), including total cross-section (*σ_T_*) for slow or thermal neutrons, by utilizing appropriate experimental procedures or theoretical and simulation methods to test glasses [8,9,10,11,12,13,14,15,22], glass ceramics [23], ceramics [24], metallic glasses [25], concretes [26,27], steels [28], alloys [29,30,31], and polymers [32,33], etc. for radiation shielding. Here, photons interact with a medium chiefly in PEA (photoelectric absorption), CS (Compton scattering), and PP (pair production) modes.

Saddeek et al. [34], for the selected four types of tellurovanadate glasses containing TiO_2_, Ag_2_O, PbO, and Bi_2_O_3_, studied µ/ρ (at photon energy range of 0.015–15 MeV by XCOM, XmuDat, and MCNPX), HVL, *Z_eff_*, *Z_eq_*, EABF, *Σ_R_*, proton and alpha mass stopping power (MSP), and projected range values (using SRIM code). They identified that among all the investigated samples, 11Bi_2_O_3_-27V_2_O_5_-62TeO_2_ (mol%) glass (VTBi6) possesses the highest µ/ρ and *Σ_R_*, and the minimum HVL quantities for *γ*-ray and neutron attenuation. In a different work, for the chosen five [(100 − *x*) SiO_2_-*x* (SnO + SnF_2_)] (*x* = 40, 45, 50, 55, and 60 mol%) composition glasses, El-Agawany et al. [35] explored µ/ρ (by both XCOM and MCNP5), µ, HVL, MFP, *Z_eff_*, EBF, and EABF, and *Σ_R_*. Here, the authors found that the SSS50 (40SiO_2_-50SnO-10SnF_2_ (mol%)) sample shows superior *γ*-ray shielding capacity among all the inspected glasses, while the SSS30 glass has the largest *Σ_R_* = 0.08931 cm^−1^. For the selected five distinct glasses in the composition of 75TeO_2_ + 15ZnO + (10 − *x*) Nb_2_O_5_ + *x*Gd_2_O_3_ (*x* = 0, 1, 1.5, 2, and 2.5 mol%), µ/ρ (applying both Geant4 code and Phy-X/PSD), *Z_eff_* (for total electrons, protons, and alpha particles interactions also), total stopping power for total electron particle interactions, MFP, HVL, *Z_eq_*, EBF, and *Σ_R_* values are determined by Al-Buriahi et al. [36], and they concluded that the TZNG-E glass (the highest Gd_2_O_3_ content added one) demonstrates superior radiation attenuation ability than the rest of the samples. Rammah [37] reported µ/ρ applying both WinXCOM and Geant4, *Z_eff_*, MFP, HVL, EBF, and *Σ_R_* for selected seven glasses in TeO_2_-B_2_O_3_-SrCl_2_-LiF-Bi_2_O_3_ system, and noticed that μ/ρ and *Z_eff_* quantities increase, while MFP and HVL values decrease with TeO_2_ content increment. Further, at 59.54, 356, 662, 1173, and 1333 keV *γ*-ray energies by WinXCOM and experimentally, for the fabricated six glasses in *x*PbO-(40 − 0.5*x*) B_2_O_3_-(40 − 0.5*x*) P_2_O_5_-9.0Na_2_O-1.0Al_2_O_3_-9.6ZnO-0.2Sm_2_O_3_- 0.2Gd_2_O_3_ (*x =* 10, 15, 20, 25, 30, and 35 mol%) composition, Singh et al. [38] investigated µ/ρ, *Z_eff_*, *N_eff_*, MFP, and HVL and found that Pb35 sample has better shielding features in terms of high µ/ρ and *Z_eff_*, and low HVL and MPF among all samples. Salama et al. [39] calculated µ/ρ, *Z_eff_*, *N_eff_*, HVL, *Z_eq_*, EBF, and *Σ_R_* quantities for the prepared *x*PbO-40B_2_O_3_-25Na_2_O-5Li_2_O-(30 − *x*) SiO_2_ (*x* = 0, 5, 10, 15, 20, and 25 mol%) glasses, using XCOM (experimentally at 0.239, 0.662, 0.911, and 1.332 MeV energies). In this work, the authors observed that µ/ρ, *Z_eff_*, and *N_eff_* values increase with the PbO content increment and decrease as the photon energy increases, and *Σ_R_* is the highest (=0.1375 cm^−1^) for 25 mol% PbO containing sample. In another work, for the chosen 26.66 B_2_O_3_-16GeO_2_-4Bi_2_O_3_-(53.33 − *x*) PbO-*x*PbF_2_ (*x* = 0, 15, 30, and 40 mol%) glass system, Kumar et al. [40] deduced µ/ρ (utilizing XCOM, Geant4 code, and MCNPX at 0.122, 0.356, 0.511, 0.662, 0.84, 1.17, 1.275, and 1.33 MeV photon energies), *Z_eff_*, *N_eff_*, MFP, and EBF values, and identified that BPBG0 sample possesses the lowest MFP in all studied glasses, indicating good *γ*-ray shielding effectiveness. Ahmad et al. [41], for the synthesized *x* [R_m_O_n_] (0.5 − *x*) [ZnO] 0.2 [B_2_O_3_] 0.3 [Soda Lime Silica (SLS)] (R_m_O_n_ = Bi_2_O_3_ and PbO, *x* = 0.05, 0.10, 0.20, 0.30, 0.40, and 0.45 mol%) glasses, reported µ/ρ (experimentally and by WinXCOM at 59.54, 122, and 662 keV *γ*-ray energies), *Z_eff_*, HVL, and MFP values, and they established that the PbZnBo-SLS glass system exhibits slightly superior shielding features than BiZnBo-SLS glass system. For (ZnO)_x_ (SLS)_1−x_ (0 ≤ x ≤ 50 wt%) glasses, Sayyed et al. [42] explored µ/ρ, HVL, *Z_eq_*, and EBF, and identified that G6 glass owns better attenuation capability than the remaining glasses owing to its relatively larger µ/ρ and minimal HVL and EBF quantities. Waly et al. [43], for six different PbO based glass compositions, computed µ/ρ (using MicroShield code), HVL, and EBF values, and found that among all selected samples, the ‘Glass 6′ (the highest PbO wt% hold one) shows the largest µ/ρ and the lowest HVL for better *γ*-ray shielding efficacy.

With a motivation to propose cost-effective glasses as radiation shields as the primary aim of this current work, we studied µ, μ/ρ, *Z_eff_*, *N_eff_*, HVL, TVL, MFP, RPE, *Z_eq_*, EBF, and EABF using Phy-X/PSD for both B_2_O_3_-Bi_2_O_3_-CaO and B_2_O_3_-Bi_2_O_3_-SrO glass systems. EBFs and EABFs are deduced up to 40 mfp PDs. Further, *Σ_R_* and σ_T_ values are also derived using Phy-X/PSD software and Geant4 code, including σ_cs_, σ_ics_, σ_A_, and σ_T_ for thermal neutrons by a suitable formula.

## 2. Materials and Methods

For the twelve chosen, i.e., six B_2_O_3_-Bi_2_O_3_-CaO and six B_2_O_3_-Bi_2_O_3_-SrO ternary glass compositions, the related measured ‘*ρ*’ was found in Ref. [44]. Selected calcium bismuth borate and strontium bismuth borate glasses are marked as ‘C1’, ‘C2’, ‘C3’, ‘C4’, ‘C5’, and ‘C6’, and ‘S1’, ‘S2’, ‘S3’, ‘S4’, ‘S5’, and ‘S6’, accordingly. For each studied sample, Table 1 and Table 2 provide composition mol% and wt%, and ‘*ρ*’. By melting proper H_3_BO_3_, Bi_2_O_3_, Ca(NO_3_)_2_·4 H_2_O, and Sr(NO_3_)_2_ chemical mixture ratios at 950 °C/3 h, utilizing covered Pt crucibles, all C1–C6 and S1–S6 glasses were prepared [44]. For all samples, ‘*ρ*’ (error < 0.003 g/cm^3^) was acquired by the hydrostatic weighing technique [44]. Following Table 1 and Table 2, one can identify that from C1 to C6 and S1 to S6 glasses, ‘*ρ*’ increases continually because of higher *M.W.* (molecular weight) and ‘*ρ*’ of Bi_2_O_3_ additive (465.96 g/mol and 8.9 g/cm^3^) as opposed to relatively lesser *M.W.* and ‘*ρ*’ B_2_O_3_ (69.63 g/mol and 2.46 g/cm^3^) and CaO (56.08 g/mol and 3.34 g/cm^3^)/SrO (103.62 g/mol and 4.7 g/cm^3^) constituents.

**Figure 1:**
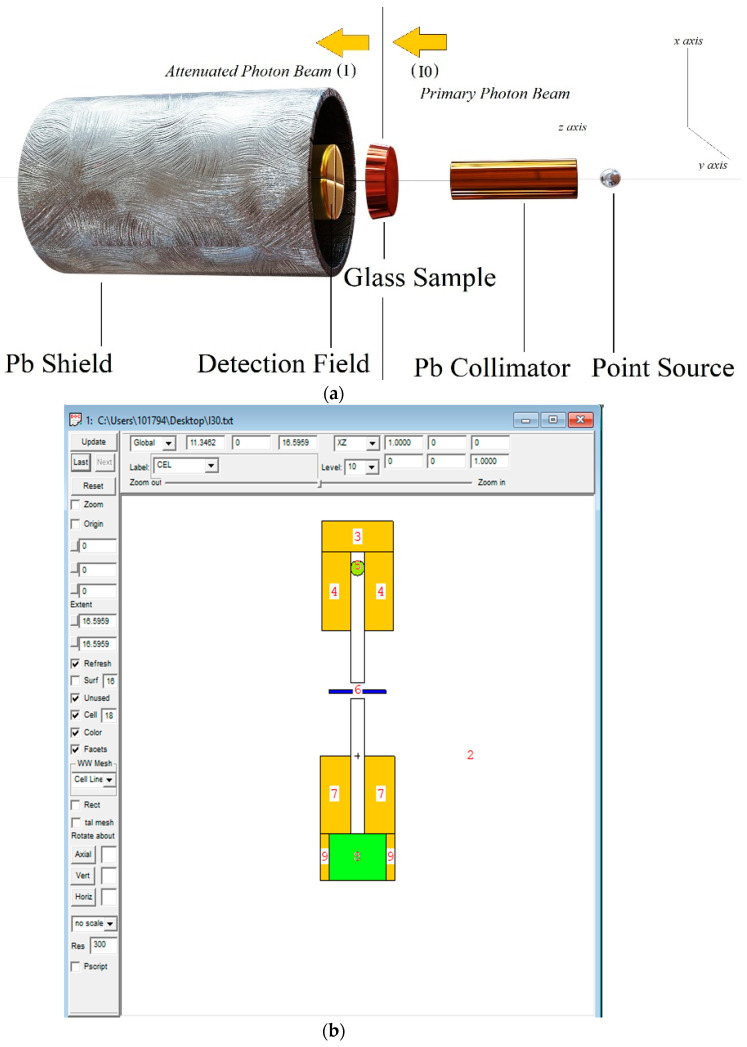
(**a**) Photon transmission setup for µ/ρ simulations of investigated glasses (3-D view) (**b**) MCNPX simulation setup (2-D view) of µ/ρ computations obtained from MCNPX Visual Editor (version X_22S).

For related details on µ, μ/ρ, *Z_eff_*, *N_eff_*, HVL, TVL, MFP, and RPE parameters, including *Z_eq_* and a five parameter G–P fitting approximation for EBFs and EABFs computation, one can review Refs. [8,9,10,11,27,29,31,32,33,34,35,36,37,38,39], as these perspectives are not restated in this part. Likewise, specifics on ‘*Σ_R_*’ and related expressions can be found elsewhere [8,9,22,27,45]. Further, narrations about the utilized WinXCOM [46] dependent Phy-X/PSD (Photon Shielding and Dosimetry) software [47] can be found in Ref. [9] and https://phy-x.net/PSD web page. Moreover, the applied MCNPX (Monte Carlo N-Particle eXtended) [48] simulation set-up and process (Figure 1), Geant4 (for GEometry ANd Tracking) code [49,50,51], and PENELOPE^®^ (Penetration and ENErgy LOss of Positrons and Electrons) code [52] descriptions are the same as those we have given in our recent works [22,45,53,54], and they are not reproduced here.

## 3. Results and Discussion

### 3.1. γ-ray Shielding Features

**Figure 2:**
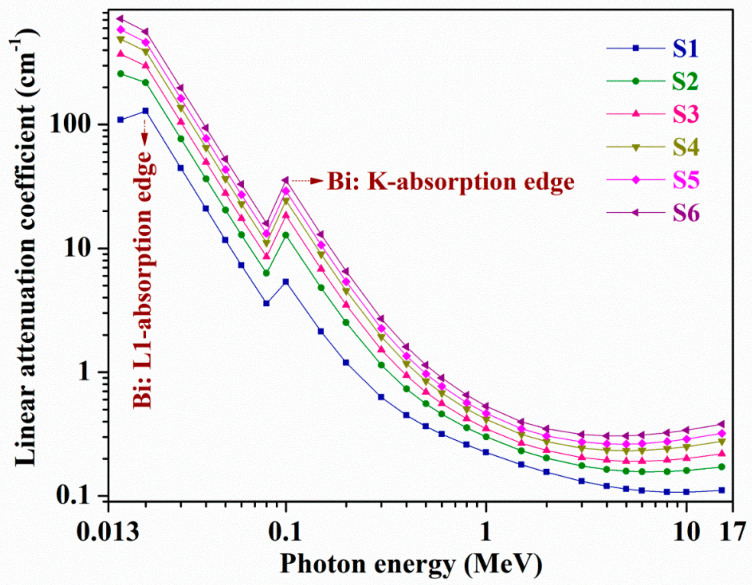
Variations of linear attenuation coefficient (µ, cm^−1^) with photon energy (MeV) for all S1–S6 glasses.

All discussed results in this sub-section are for the 0.015–15 MeV photon energy range. For all S1–S6 samples, Figure 2 shows the variations of ‘µ’, calculated utilizing Phy-X/PSD, whereas for all C1–C6 samples obtained ‘µ’ variations are depicted in Figure S1 of the Supplementary Material. Following Figure 2 and Figure S1, one can find that with photon energy increment, ‘µ’ has an identical *γ*-rays reliance, and it increases considerably with the increasing Bi_2_O_3_ content, i.e., larger wt% of high *Z* (Bi (*Z* = 83)) element in place of proportionately lower *Z* constituents, B (*Z* = 5)/Ca (Z = 20)/Sr (Z = 38) in samples C1 to C6 and S1 to S6. Among all C1–C6 and S1–S6 samples, glass S6 has, relatively, the highest ‘µ’ due to the largest wt% of Bi (=80.6813 wt%, see Table 2) in it.

Except for S1 glass, the remaining all samples possess maximal ‘µ’ at 15 KeV energy while sample S1 holds maximum ‘µ’ (=128.586 cm^−1^) at 20 KeV energy owing to Bi: L1-absorption edge (L1-absorption edge—Bi: 16.3875 KeV). At 0.015 MeV energy, 101.093, 194.0115, 330.0832, 449.4279, 505.5198, and 641.4454 cm^−1^ are the obtained ‘µ’ values for samples C1, C2, C3, C4, C5, and C6, respectively, while they are 109.24, 256.982, 369.9715, 490.5416, 586.3835, and 716.641 cm^−1^, accordingly, for S1 to S6 glasses. Within a lower 15 KeV–0.4 MeV energy range, on account of photoelectric absorption (PEA) (∝ E^−3.5^) supremacy [55], for all chosen samples, a sharp decrement in ‘µ’ is observed. As an example, for C6 and S6 glasses, at 0.04 and 0.4 MeV energies, the derived ‘µ’ quantities are 82.55021 and 1.454471 cm^−1^ and 93.97439 and 1.604014 cm^−1^, respectively. For all C1–C6 and S1–S6 glasses, at 0.1 MeV, a quick rise in ‘µ’ transpired because of Bi: K-absorption edge at 90.5259 KeV (see Figure S1 and Figure 2). Then, for C1 and S1 glasses from 0.5 to 10 MeV, C2 sample from 0.5 to 8 MeV, C3 glass from 0.5 to 6 MeV, C4, C5, and C6 samples from 0.5 to 5 MeV, S2 and S3 samples from 0.5 to 6 MeV, S4 and S5 glasses from 0.5 to 5 MeV, and S6 glass from 0.5 to 4 MeV energies, changes or reductions in ‘µ’ are very small as the Compton scattering (CS) (∝ E^−1^) mechanism [55] controls these intermediate energy ranges. Next, in the higher energy regions, i.e., for C1 and S1 samples, above 10 MeV; for C2 glass, after 8 MeV; for C3 sample, beyond 6 MeV; for C4, C5, and C6 glasses, after 5 MeV; for S2 and S3 glasses, above 6 MeV; for S4 and S5 samples, after 5 MeV; and for S6 sample, beyond 4 MeV up to 15 MeV, a slight hike in ‘μ’ owing to pair production (PP) (∝ log E) [55] phenomenon command is identified. For instance, at 4, 5, and 15 MeV energies, for S6 glass, the calculated ‘µ’ values are 0.3052, 0.3057, and 0.3809 cm^−1^, accordingly. By emitting minimal penetrating capability charged particles, commonly, in both PEA and PP actions, photons can be fully absorbed by the substances whereas the CS process results in photons energy partial degradation only and allows them to possess significant leftover energy for larger penetration depths to reach that lead to bigger fleeing probabilities. From the achieved ‘µ’ results, one can see that for the lowest energy photons absorption or reduction, all selected C1–C6 and S1–S6 samples are good.

For all C1–C6 and S1–S6 glasses, Figure S2a–l (see Supplementary Material) illustrates the derived μ/ρ (by Phy-X/PSD software, MCNPX, Geant4, and Penelope codes) comparison, accordingly, and in Tables S1 and S2ⅰ–ⅳ, the respective μ/ρ results are listed. A quality congruity is noticed among all respective derived μ/ρ quantities (see Tables S1 and S2 data and Figure S2). For example, for sample C6, at 30 KeV energy, by Phy-X/PSD, MCNPX, Geant4, and Penelope code 24.851, 24.963, 24.837, and 24.362 cm^2^/g are the obtained individual μ/ρ quantities, while they are 26.094, 26.105, 26.080, and 25.589 cm^2^/g, accordingly, for the S6 glass at the same energy. Likewise, at 15 MeV energy, 0.0489, 0.04905, 0.0489, and 0.0489 cm^2^/g are the deduced corresponding Phy-X/PSD, MCNPX, Geant4, and Penelope code μ/ρ for C6 glass, whereas at the same energy, for sample S6, 0.0502, 0.0503, 0.0502, and 0.0501 cm^2^/g are the respective μ/ρ values. All C1–C6 and S1–S6 glasses show identical variations in μ/ρ with energy (15 KeV to 15 MeV) and from samples C1 to C6 and S1 to S6 because of ‘*ρ*’ improvement (3.104 to 6.993 g/cm^3^ and 3.572 to 7.59 g/cm^3^, see Table 1 and Table 2), μ/ρ ascertainably increased, for instance, at 15 KeV energy, 32.569, 50.21, 68.51, 80.572, 83.502, and 91.727 cm^2^/g, and 30.582, 56.867, 71.979, 81.391, 87.835, and 94.419 cm^2^/g, are the relevant μ/ρ (utilizing Phy-X/PSD) calculated for C1, C2, C3, C4, C5, and C6, and S1, S2, S3, S4, S5, and S6 glasses. Here, the larger the photon energy, the bigger the photons’ penetration ability through the glasses. As clarified for Figure 2 ‘µ’ outcomes, for all C1–C6 and S1–S6 glasses, for μ/ρ changes with *γ*-ray energy also, PEA (lowest energy region (15 KeV–0.4 MeV), quick decline), CS (medium energy ranges (0.5–(4–10) MeV, less reduction), and PP (greater energy regions ((>4–10)→15 MeV), minimal increase) phenomena play crucial roles. In their respective series, samples C6 and S6 possess relatively the larger μ/ρ, and again, S6 glass has higher μ/ρ than sample C6 at all selected energies, specifying its superior photon attenuation capacity.

In Figures S3–S7 of the Supplementary Material, we presented all the changes of *Z_eff_*, *N_eff_*, HVL, TVL, and MFP values within inspected *γ*-rays energy range for all C1–C6 and S1–S6 glasses with relevant discussion.

**Figure 3:**
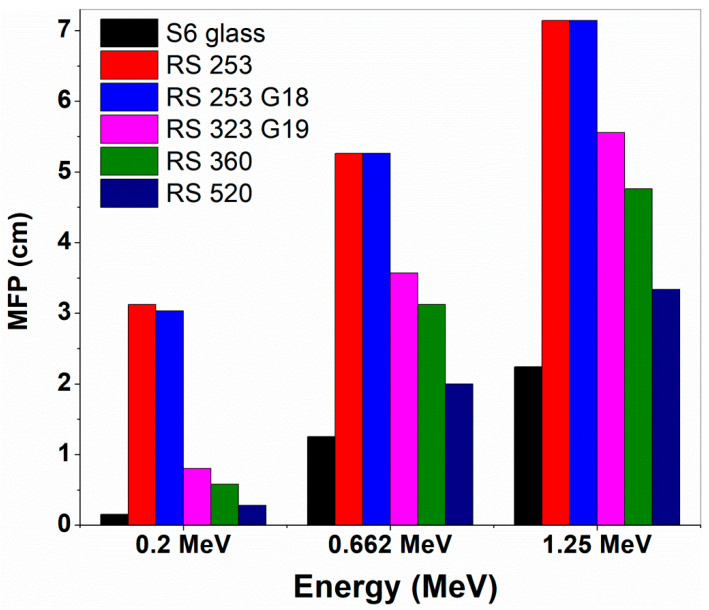
Comparison of MFP of the glass ‘S6′ with some commercial glasses.

Figure 3 demonstrates the sample S6 MFP comparison with relevant commercial shielding glasses’ [56] values, and likewise glass S6 HVL comparison with these commercial glasses is shown in Figure S8 of the Supplementary Material. Here, at all three corresponding 0.2 MeV, 0.662 MeV (^137^Cs), and 1.25 MeV (^60^Co) *γ*-ray energies, sample S6 has less HVL and MFP than the commercial glasses (see Figure S8 and Figure 3). So sample S6 has superior *γ*-ray shielding capacity than the compared commercial glasses owing to its′ larger ‘µ’ than them. Earlier, SS403, CN, CS516, IL600, and MN400 alloys [57], C_5_H_8_, C_3_H_3_N, C_5_H_8_O_2_, C_10_H_8_O_4_, CH_2_O, and C_10_H_10_O_2_ polymers [58], and concretes, such as OC, BMC, HSC, IC, ILC, SMC, and SSC [59] were also reported for nuclear radiation shielding purpose by other researchers.

For high *Z* elements (e.g., Bi) containing compounds, generally, at <0.5 MeV photon energies, the PEA (all photon energy fully passing on to a bound electron) is so common, and the CS phenomenon prevails at relatively moderate and greater *γ*-ray energies (~500 KeV–1.5 MeV). Further, to undergo the PP process, incident photons must possess energies >1.022 MeV, specifically in larger *Z* substances, and subsequently for the two 511 keV *γ*-rays (owing to positron + electron annihilation) generations that go separately in opposed directions. Here, with improving *Z*, the atomic cross-section for a certain PEA, CS, and PP phenomena enhances.

**Figure 4:**
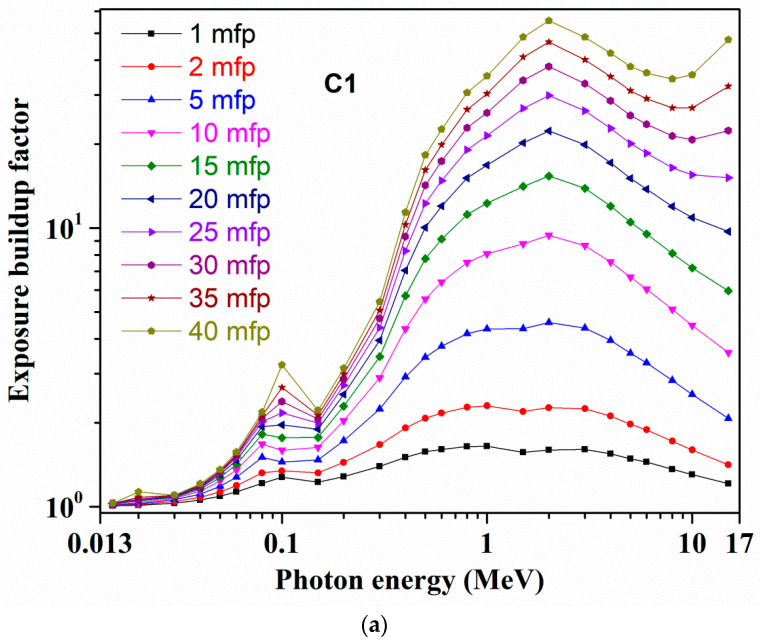

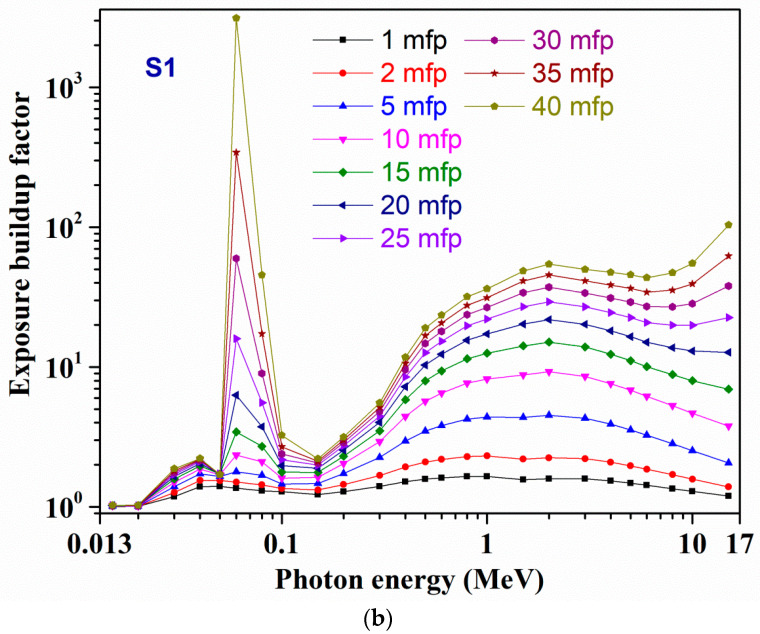
Variations of exposure buildup factor (EBF) with photon energy at different mean free paths for (**a**) C1 and (**b**) S1 glasses.

**Figure 5:**
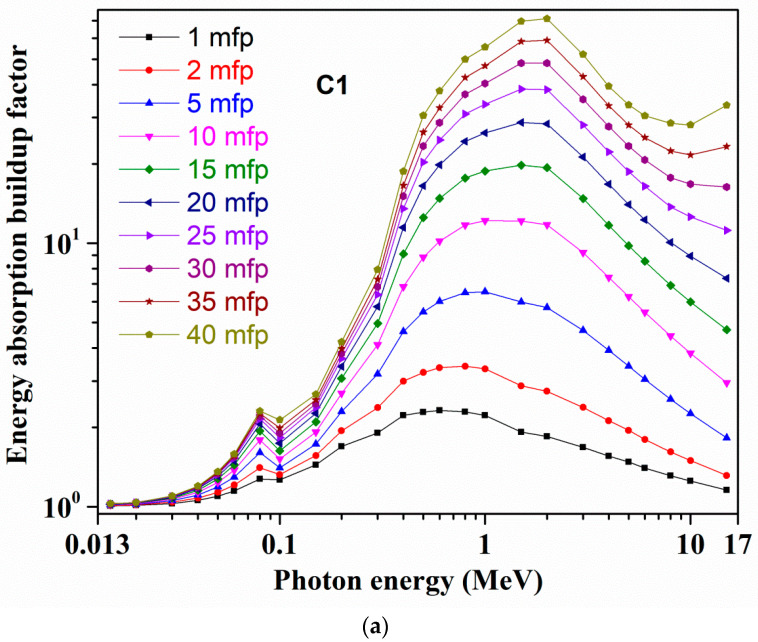

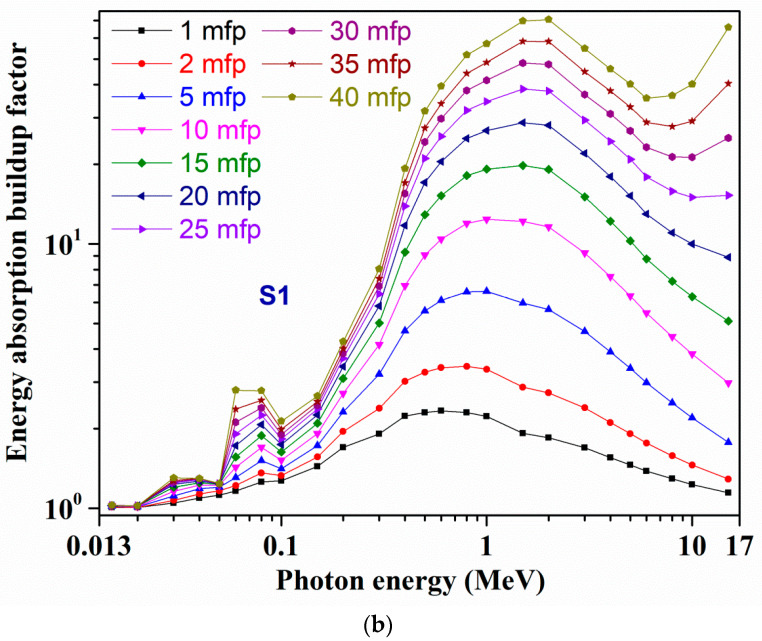
Variations of energy absorption buildup factor (EABF) with photon energy at different mean free paths for (**a**) C1 and (**b**) S1 glasses.

Figure 4a,b and Figure 5a,b show, for C1 and S1 glasses, at 1–40 mfp PDs the computed EBFs and EABFs variations appropriately. For all the remaining C2–C6 and S2–S6 samples, the respective EBFs and EABFs variations at 1–40 mfp PDs are presented in Figures S9a–j and S10a–j in the Supplementary Material. The computed *Z_eq_* and related G–P fitting parameters for EBFs and EABFs derivations for all C1–C6 and S1–S6 samples are tabulated in Tables S3–S14 of the Supplementary Material, accordingly. All studied glasses EBFs and EABFs exhibit an alike course with photon energy along with a sharp increase in these values at respective 0.02, 0.03, 0.06, 0.08, and 0.1 MeV energies owing to ‘Bi’ L1 and K-absorption edges. At lower *γ*-ray energies, i.e., from 15 KeV up to 150 KeV, EBFs and EABFs hold minimal quantities with negligible deviations, except the mentioned rises. As stated earlier, the PEA process commands this lower energy region. Beyond 0.015 MeV up to 1–2 MeV energy (up to 0.06/0.08 MeV at 1 mfp/2 mfp lower penetration depths) for C1 and S1 glasses, corresponding EBFs and EABFs are progressively improved, while for C2–C6 and S2––S6 samples these quantities are enhanced up to 2–3 MeV at higher ‘mfp’ due to CS mechanism (multiple scattered photons) supremacy over this moderate energy range. Usually, EBFs and EABFs move to larger energies for greater *Z_eq_* compounds. Then, with increasing *γ*-ray energy up to 15 MeV both C1 and S1 glasses show a complete decreasing trend in according EBFs and EABFs from 1 to 15 mfp, and at larger mfp, these values slowly increase at higher energies. For the remaining samples also, at smaller penetration depths, EBFs and EABFs changes are smaller up to 15 MeV energy, while they considerably enhance at greater mfp with energy rise up to 15 MeV. Usually the ‘buildup’ of photons appears at the bigger mfp, specifically for higher thickness substances and diversity of the incoming *X*-rays or *γ*-rays. Thus, relying on glass chemical composition (C1 to C6 and S1 to S6 glasses), for the obtained EBFs and EABFs alterations at bigger photon energies, the PP process dominates. Generally at higher energies and greater penetration depths, secondary photons scatterings happen frequently, leading to larger buildups. Overall, absorption activities lower the EBFs and EBFs whereas scattering phenomena improve them. Because of comparatively greater *Z_eq_*/*Z_eff_*, in all selected C1–C6 and S1–S6 glasses, sample S6 possesses the lesser EBFs and EABFs, indicating it as a more potent photon attenuator.

**Figure 6:**
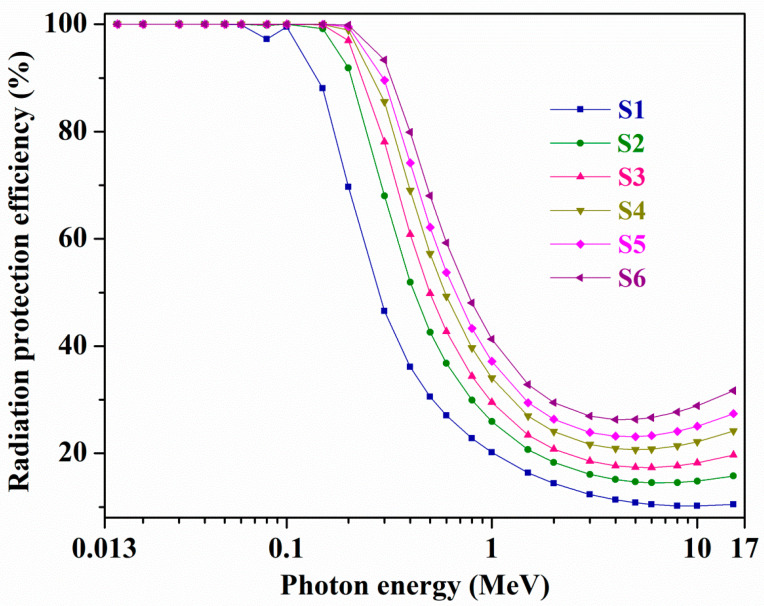
Variations of radiation protection efficiency (RPE) with photon energy (MeV) for all S1–S6 glasses.

For all C1–C6 and S1–S6 samples (thickness, *t* = 1 cm), Figure S11 in the Supplementary Material and Figure 6 portrays variations of evaluated RPE quantities, separately. From samples C1 to C6 and S1 to S6 with improving Bi_2_O_3_ content from 5 to 50 mol% and 5 to 60 mol% respectively, RPE quantities are increased owing to Bi (*Z* = 83), a heavy element, which boosts the glass capacity in reducing incoming photons’ intensity. Here among all studied samples, glass S6 (contains 60 mol% Bi_2_O_3_) has the relatively largest RPE at all energies. From 15 KeV up to 0.06 MeV energy, RPE has the biggest values for all glasses, indicating all samples’ exceptional competence in obstructing the low energy *γ*-rays. As an example, the computed RPE values for C1, C2, C3, C4, C5, and C6 glasses at 15 and 60 KeV energies are 100% (for all samples) and 98.94%, 99.98%, 100%, 100%, 100%, and 100% accordingly, and these quantities at the same energies for S1, S2, S3, S4, S5, and S6 samples are 100% (for all glasses) and 99.93%, 100%, 100%, 100%, 100%, and 100% correspondingly. Further, beyond 0.1–0.2 MeV energy range, all C1–C6 and S1–S6 glasses RPE values are quickly decreased with enhancing photon energy, as evidenced from both Figure S11 and Figure 6, which means higher energy photons can easily pass through the samples. At 0.15 and 1.5 MeV energy, 84.96%, 97.05%, 99.76%, 99.97%, 99.99%, and 100%, and 14.75%, 18.14%, 22.19%, 25.32%, 27.17%, and 30.73%, respectively, are the RPE quantities for C1 to C6 samples, while at the same energy, they are 88.09%, 99.18%, 99.89%, 99.99%, 100%, and 100%, and 16.37%, 20.7%, 23.4%, 26.94%, 29.45%, and 32.82%, accordingly, for S1 to S6 glasses. This indicates that, for instance, samples C6 and S6 can effectively shield only 30.73% and 32.82% of the incoming 1.5 MeV energy *γ*-rays and the rest of the 69.27% and 67.18% of the *γ*-rays can go through these glasses. Next, within the 2–15 MeV energy range, the depletion and/or variation in RPE is small for all chosen glasses, for example, samples C6 and S6 owns the RPE quantities 27.53% and 28.97%, 29.48% and 31.68%, accordingly, at 2 MeV and 15 MeV energies. Further, S6 glass exhibits the minimum RPE (=26.31%) for 4 MeV energy photons. Based on the RPE outputs, one can affirm that sample S6 has an excellent shielding efficiency, specifically for lower energy *γ*-rays, among all selected C1–C6 and S1–S6 glasses.

### 3.2. Neutron Attenuation Aspects

Tables S15 and S16 of the Supplementary Material present the *Σ_R_* calculation processes and the corresponding *Σ_R_* quantities for all C1–C6 and S1–S6 samples, accordingly. From Tables S15 and S16 data, from samples C1 to C6 and S1 to S6, one can see that the *Σ_R_* values are improved at 0.1064–0.1198 cm^−1^ and 0.1076–0.1199 cm^−1^ ranges accordingly, because of enhanced shares of Bi element in them. Overall, among all C1–C6 and S1–S6 glasses, owing to larger ‘*ρ*’ (=7.59 g/cm^3^), sample S6 owns the larger *Σ_R_* (=0.1199 cm^−1^) with a collective contribution of all elements (O, Sr, B, and Bi) *Σ_R_*, which is very slightly higher than C6 glass *Σ_R_* (=0.1198 cm^−1^, *ρ* = 6.993 g/cm^3^), as substances ‘*ρ*’ play a principal role in fast neutrons shielding. Hence, sample S6 (S6—B: 2.0869 wt%, Bi: 80.6813 wt%, Sr: 2.8189 wt%, and O: 14.4128 wt%) has a superior ability for fast neutron attenuation. The derived *Σ_R_* results specify that for any glass, the proper mixture of both light (e.g., B) and heavy (e.g., Bi) elements is essential in attaining better attenuation effectiveness for fast neutrons. Additionally, glass S6 *Σ_R_* value is compared with distinct established (e.g., graphite, H_2_O, concretes) and some other freshly reported radiation shielding substances *Σ_R_* quantities [8,22,23,31,33,34,35,36,39,45,53,55,59] and listed in Table 3.

Following Table 3 data, one can identify that, proportionately, S6 glass possesses a greater *Σ_R_* than respective graphite, H_2_O, OC, HSC, and ILC [59], G8 [8], and G0 [22] glasses, S0 glass-ceramic [23], PA-6 and PVDC polymers [33], SSS30 [35], and TZNG-E [36] glasses values, and a lower *Σ_R_* than BBLNi6 [55] glass, NS1 alloy and SMC [31], and VTBi6 [34], S5 [39], E [45], and S7 [53] glasses quantities.

**Figure 7:**
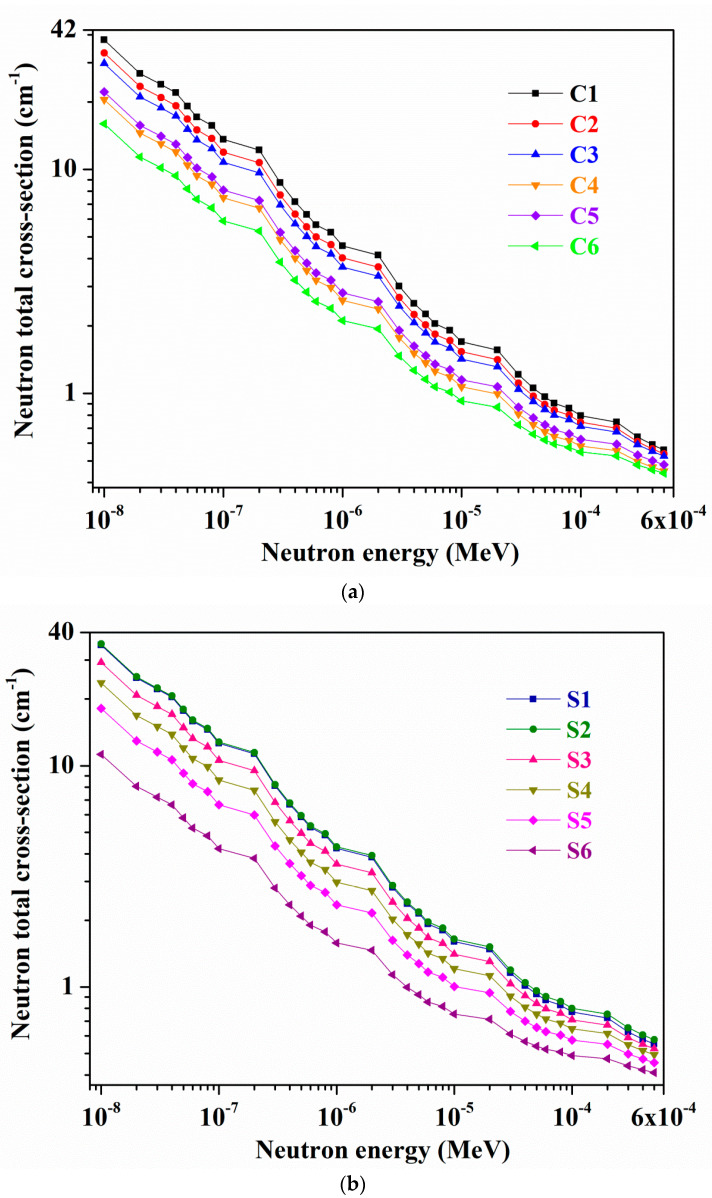

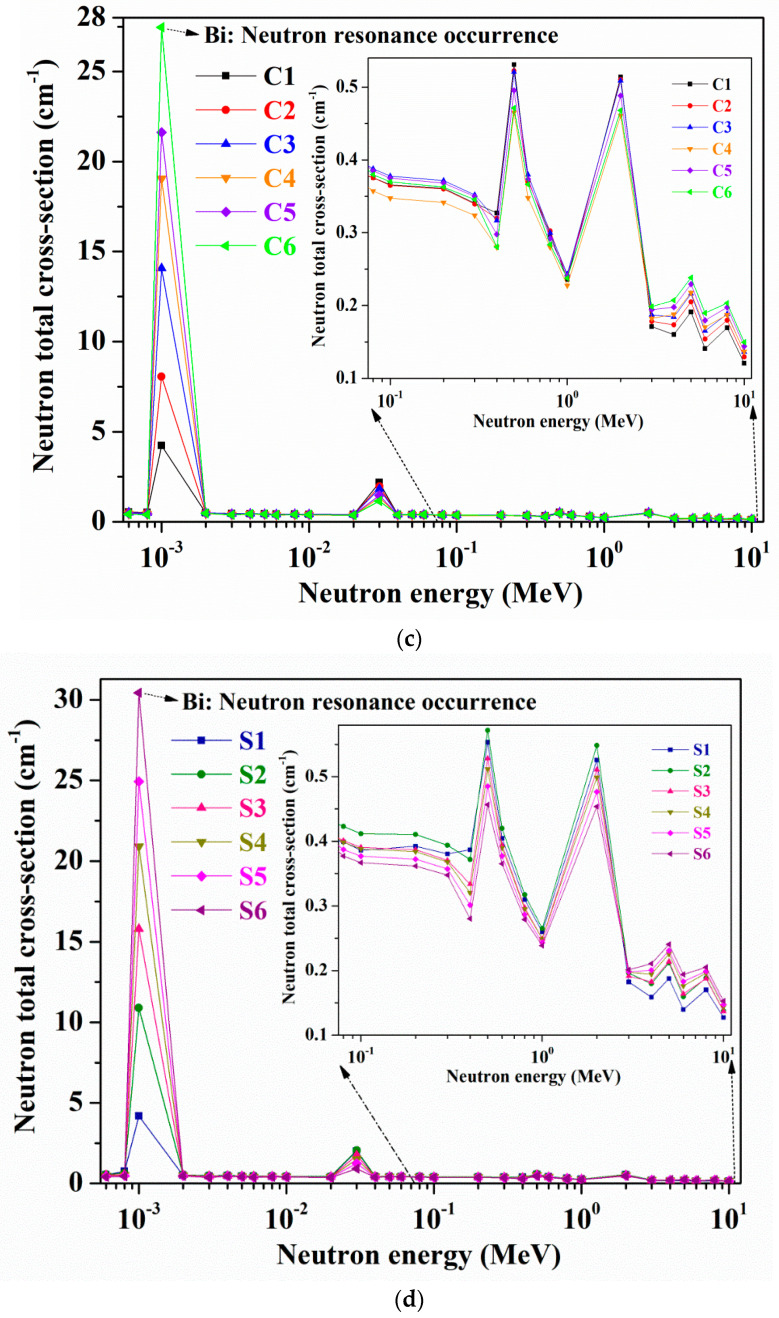
Variations of neutron total cross-section (*σ_T_*) within the neutron energy range of (**a**,**b**) (1 × 10^−8^–5 × 10^−4^ MeV), and (**c**,**d**) (6 × 10^−4^–10 MeV) (insets, within the ranges of 0.075–10 MeV energies) for all C1–C6 and S1–S6 glasses.

For all C1–C6 and S1–S6 glasses, the variations in ‘*σ_T_*’ quantities at 1 × 10^−8^–5 × 10^−4^ MeV and 6 × 10^−4^–10 MeV neutron energies, derived by applying the Geant4 code, are shown in Figure 7a–d, individually. Here inset plots of Figure 7c,d depict zoom-in neutron energy ranges at 0.075–10 MeV, respectively. At all chosen distinct neutron energies within the range of 1 × 10^−8^–5 × 10^−4^ MeV, among C1–C6 and S1–S6 samples, the σ_T_ values are increased in the order C1 > C2 > C3 > C5 > C4 > C6 and S2 > S1 > S3 > S4 > S5 > S6 (see Figure 7a,b) contingent upon the B_2_O_3_ content in them. Though sample S1 possesses higher wt% of ‘B’ (=14.1055 wt%) than S2 glass (B: 11.2807 wt%), sample S2 owns slightly larger ‘*σ_T_*’ values, which might be owing to some contribution of greater wt% of Bi (=46.727 wt%) element in it than S1 glass (Bi: 20.9742 wt%) (see Table 2). One can notice a similar result for C4 and C5 glasses ‘*σ_T_*’ (i.e., C5 > C4) also (C4—B: 5.2555 wt%, Bi: 67.7275 wt%, and C5—B: 5.2402 wt%, Bi: 70.9069 wt%) (see Table 1). However, for equal molar ratio B_2_O_3_ containing samples, i.e., C2 and C3 and S1 and S3, ‘B’ element wt% appear to solely play a principal role in the C2 and S1 samples enhanced ‘*σ_T_*’ quantities than respective C3 and S3 glasses values. Besides, overall, within range of 0.01 eV–10 MeV, for all studied glasses, σ_T_ values are decreased with increasing energy. Among all selected samples, glass C1 holds comparatively the bigger σ_T_ values, for example, at 0.01 eV and 1 eV energies. At 0.01 eV neutron energy, 37.9099, 33.1434, 29.7834, 20.5163, 22.1935, and 15.9836 cm^−1^, and 35.0324, 35.4587, 29.2474, 23.6623, 18.1469, and 11.2638 cm^−1^ are the σ_T_ quantities for C1, C2, C3, C4, C5, and C6, and S1, S2, S3, S4, S5, and S6 glasses, accordingly, while they are 4.5518, 4.0242, 3.6567, 2.6013, 2.8082, and 2.1116 cm^−1^, and 4.2295, 4.2997, 3.5927, 2.9719, 2.3509, and 1.5796 cm^−1^ for the same respective samples at 1 eV energy. Further, all C1–C6 and S1–S6 glasses have smaller *σ_T_* values with minimal changes at 6 × 10^−4^–10 MeV energies (see Figure 7c,d).

For instance, at 600 eV neutron energy, from C1 to C6 glass, simulated *σ_T_* values are 0.5400, 0.5196, 0.5080, 0.4366, 0.4652, and 0.4277 cm^−1^, whereas for S1, S2, S3, S4, S5, and S6 samples, they are 0.5337, 0.5579, 0.5076, 0.4783, 0.4411, and 0.3985 cm^−1^, accordingly. Moreover, from Figure 7c,d, one can notice an acute increase in *σ_T_* with a strong peak at 1 KeV energy because of an occurrence of the resonance between the neutron energy and the ‘Bi’ nucleus [60] that present in all the samples. Usually, various nuclides, at a particular or pretty limited neutron energy region, possess the large ability for interactions with neutrons, and consequently, higher *σ_T_* occurs at these neutron energies (i.e., intense peaks in *σ_T_* against energy figures) [61]. From samples C1 to C6 and S1 to S6, with increasing Bi_2_O_3_ content in the glasses, the *σ_T_* for the identified ‘Bi’ resonance peak at 1 KeV progressively increases, having maximal values for C6 and S6 samples in related glass series, where again glass S6 has the larger *σ_T_* than C6 sample (C6—Bi: 78.4356 wt%, S6—Bi: 80.6813 wt%) (see Table 1 and Table 2). At 1 KeV energy, C1, C2, C3, C4, C5, and C6 samples exhibit 4.2229 cm^−1^, 8.0614 cm^−1^, 14.0697 cm^−1^, 19.0511 cm^−1^, 21.6176 cm^−1^, and 27.4667 cm^−1^ *σ_T_* quantities while 4.1840 cm^−1^, 10.8921 cm^−1^, 15.7994 cm^−1^, 20.8961 cm^−1^, 24.9439 cm^−1^, and 30.4472 cm^−1^ are the corresponding S1–S6 glasses deduced *σ_T_* values. Based on the *σ_T_* outcomes, among all chosen glasses, one can identify that sample C1 (B: 17.5887 wt%, Bi: 24.2855 wt%, Ca: 11.6436 wt%, and O: 46.4821 wt%) has high potentiality for thermal or slow neutrons and intermediate energy neutrons capture or absorption.

Further, applying related formula reported in Ref. [45], for all C1–C6 and S1–S6 glasses, at 0.0253 eV neutron energy, ‘σ_cs_’, ‘σ_ics_’, ‘σ_A_’, and ‘σ_T_’ are assessed and corresponding quantities are listed in Table S17i,ii of the Supplementary Material, respectively. Also, Table S18 in the Supplementary Material presents all the B, Bi, Ca, Sr, and O elements ‘σ_cs_’, ‘σ_ics_’, ‘σ_A_’, and ‘σ_T_’ values, which are contained in C1–C6 and S1–S6 glasses. Relying on Tables S17 and S18 data, one can observe that in all studied samples, C1 glass exhibits relatively bigger ‘*σ_T_*’ for thermal neutrons’ absorption, followed by the S2 sample. Also, obtained ‘*σ_T_*’ values of all samples nicely coincide with 0.03 eV energy neutrons ‘*σ_T_*’ results simulated by Geant4 for them. Finally, computational techniques, such as Geant4 and MCNPX, are promising because of relevant data availability in large size for designing complex media and novel structures. These simulation processes are also useful to determine µ/ρ for distinct radiation shields at various energies and can be chosen as best scenarios in place of experimental procedures. Interestingly, compared to ‘S6′ (20SrO-60Bi_2_O_3_-20B_2_O_3_ (mol%)) glass reported by Sayyed et al. [62], in our work, ‘S6′ (30B_2_O_3_-60Bi_2_O_3_-10SrO (mol%)) sample possesses superior photon and neutron (*Σ_R_* = 0.10521 cm^−1^ [62]) attenuation factors owing to its relatively larger ‘*ρ*’ [‘S6′ glass ‘*ρ*’ = 6.892 g/cm^3^ [62]]. This establishes a fact that by simply tuning the glass composition with the same components one can achieve more favorable shielding qualities through obtaining bigger ‘*ρ*’.

## 4. Conclusions

For a total of twelve calcium bismuth borate and strontium bismuth borate selected samples, radiation shielding aspects were explored by estimating μ, μ/ρ, *Z_eff_*, *N_eff_*, HVL, TVL, MFP, *Z_eq_*, EBF, EABF, RPE, *Σ_R_*, and σ_T_ quantities. Among all C1–C6 and S1–S6 glasses, owing to its higher ‘*ρ*’, sample S6 owns accordingly larger ‘μ/ρ’ values, for instance, obtained by Geant4 code, at 0.015 MeV energy, 32.548, 50.1753, 68.4595, 80.511, 83.4382, and 91.6563 cm^2^/g, and 30.4967, 56.8002, 71.9114, 81.3181, 87.7584, and 94.3393 cm^2^/g, respectively, were ‘μ/ρ’ for C1, C2, C3, C4, C5, and C6, and S1, S2, S3, S4, S5, and S6 samples. Likewise, at any definite photon energy, the inclusion of Bi_2_O_3_ for B_2_O_3_ (*ρ* = 2.46 g/cm^3^)/CaO (3.34 g/cm^3^)/SrO (*ρ* = 4.7 g/cm^3^) leads to *Z_eff_* enhancement in all C1–C6 and S1–S6 glasses. Glass S6 owns the lowest HVL (=1.819 cm), TVL (=6.044 cm), and MFP (=2.625 cm) in all C1–C6 and S1–S6 samples, for instance, at 15 MeV energy. For computed EBFs and EABFs of all C1–C6 and S1–S6 glasses, up to 40 mfp PDs at discrete intervals, relevant PEA, CS, and PP phenomena at the lowest, intermediate, and higher energy ranges show preeminence. Amongst all, for S6 glass, comparatively the bigger ‘*ρ*’ (7.59 g/cm^3^), claimed greater μ, μ/ρ, *Z_eff_*, *Z_eq_*, and RPE, minimal HVL, TVL, MFP, EBFs, and EABFs hints on its’ high shielding aptitude for *γ*-rays. Thus, for the patients’ and occupational workers’ safety at nuclear medicine centers, and at nuclear power plants for radiation workers’ protection, in a non-toxicity point of view, S6 glass is a better alternative to Pb-glass and its derivatives in terms of *γ*-ray attenuation. Moreover, higher *Σ_R_* (=0.1199 cm^−1^) achieved for sample S6 indicates its superior fast neutrons’ attenuation ability. Sample C1 possesses relatively bigger ‘*σ_T_*’ values, consequently, good absorption or capture capability for thermal or slow neutrons, in all C1–C6 and S1–S6 samples, for example, at 0.02 eV neutron energy, 26.8141, 23.4572, 21.0931, 14.557, 15.7457, and 11.3698 cm^−1^, and 24.88, 25.1898, 20.7932, 16.8448, 12.943, and 8.07484 cm^−1^ were the σ_T_ quantities for C1, C2, C3, C4, C5, and C6, and S1, S2, S3, S4, S5, and S6 glasses, accordingly, while they are 19.1652, 16.7814, 15.1033, 10.4515, 11.3026, and 8.19143 cm^−1^, and 17.7146, 17.9406, 14.8238, 12.0295, 9.26565, and 5.81853 cm^−1^ for the same respective samples at 0.05 eV energy. Thus, C1 glass can be useful to prevent neutron radiation leaks that may occur at radioactive waste transport and storage sites.

## Figures and Tables

**Table 1:** Chemical composition (mol%) and elements (wt%) present in the selected B_2_O_3_-Bi_2_O_3_-CaO glasses, including their density [44].

Glass Code	Glass Composition (mol%)			Elemental Composition (wt%)				Density (g/cm^3^)
	B_2_O_3_	Bi_2_O_3_	CaO	B	Bi	Ca	O	
C1	70	5	25	17.5887	24.2855	11.6436	46.4821	3.104
C2	60	10	30	12.3329	39.7334	11.4300	36.5035	3.864
C3	60	20	20	8.8748	57.1846	5.4834	28.4571	4.818
C4	45	30	25	5.2555	67.7275	5.4119	21.6049	5.578
C5	50	35	15	5.2402	70.9069	2.9139	20.9388	6.054
C6	40	50	10	3.2461	78.4356	1.5042	16.8139	6.993

**Table 2:** Chemical composition (mol%) and elements (wt%) present in the selected B_2_O_3_-Bi_2_O_3_-SrO glasses, including their density [44].

Glass Code	Glass Composition (mol%)			Elemental Composition (wt%)				Density (g/cm^3^)
	B_2_O_3_	Bi_2_O_3_	SrO	B	Bi	Sr	O	
S1	65	5	30	14.1055	20.9742	26.3817	38.5385	3.572
S2	70	15	15	11.2807	46.7270	9.7957	32.1965	4.519
S3	65	25	10	8.1661	60.7131	5.0910	26.0296	5.140
S4	55	35	10	5.6164	69.0881	4.1381	21.1573	6.027
S5	45	45	10	3.8707	74.8221	3.4856	17.8214	6.676
S6	30	60	10	2.0869	80.6813	2.8189	14.4128	7.590

**Table 3:** Comparison of *Σ_R_* (cm^−1^) of glass S6 with reported different nuclear radiation shielding materials.

Sample	*Σ*_R_	Reference
S6 glass	0.1199	Present work
Graphite (C)	0.0771	[55]
Water (H_2_O)	0.1024	
BBLNi6 glass	0.1383	
Ordinary concrete (OC)	0.0937	[59]
Hematite-serpentine concrete (HSC)	0.0967	
Ilmenite-limonite concrete (ILC)	0.0950	
G8 glass	0.0984	[8]
G0 glass	0.11326	[22]
S0 glass-ceramic	0.11357	[23]
NS1 alloy	0.163	[31]
Steel-magnetite concrete (SMC)	0.142	
PA-6 polymer	0.1151	[33]
PVDC polymer	0.07058	
VTBi6 glass	0.1302	[34]
SSS30 glass	0.08931	[35]
TZNG-E glass	0.1125	[36]
S5 glass	0.1375	[39]
E glass	0.1474	[45]
S7 glass	0.1232	[53]

## Data Availability

Data is contained within the article or Supplementary Material.

## Supplementary Materials

The following are available online at https://www.mdpi.com/article/10.3390/ma14092265/s1, Variations of linear attenuation coefficient (µ, cm^−1^) with photon energy (MeV) for all C1–C6 glasses (Figure S1)**,** comparison of Phy-X/PSD program, MCNPX, Geant4, and Penelope codes calculated mass attenuation coefficients (µ/ρ, cm^2^/g) versus photon energy for all C1–C6 and S1–S6 glasses (Figure S2, Tables S1 and S2), variations of *Z_eff_*, *N_eff_*, HVL, TVL, and MFP of all C1–C6 and S1–S6 glasses (Figures S3–S7) with relevant discussion, comparison of HVL of the glass ‘S6’ with some commercial glasses (Figure S8), variation of exposure buildup factor (EBF) and energy absorption buildup factor (EABF) with photon energy at different mean free paths for all C2–C6 and S2–S6 glasses (Figures S9 and S10), for all the C1–C6 and S1–S6 samples, calculated equivalent atomic numbers (*Z_eq_*), and G–P fitting parameters for EBF and EABF estimations within the photon energy range of 0.015–15 MeV (Tables S3–S14), variations of radiation protection efficiency (RPE) with photon energy (MeV) for all C1–C6 glasses (Figure S11), effective removal cross-sections for fast neutrons, *Σ_R_* (cm^−1^), for all C1–C6 and S1–S6 glasses (Tables S15 and S16), coherent scattering cross-section (σ_cs_), incoherent scattering cross-section (σ_ics_), absorption cross-section (σ_A_), and total cross-section (σ_T_) of all C1–C6 and S1–S6 glasses for thermal neutron attenuation (Table S17), and (σ_cs_, barn), (σ_ics_, barn), (σ_A_, barn), and (σ_T_, barn) of B, Bi, Ca, Sr, and O elements for thermal neutrons (Table S18) can be found in the Supplementary Materials to this article.
