# Supplementary material for: Detailed Inspection of γ-ray, Fast and Thermal Neutrons Shielding Competence of Calcium Oxide or Strontium Oxide Comprising Bismuth Borate Glasses
Source: Materials (Basel). 2021 Apr 27;14(9):2265. doi: 10.3390/ma14092265 (PMC8124740; doi:10.3390/ma14092265)
Supplement: Supplementary file 1 [file materials-14-02265-s001.zip › materials-1129769-supplementary.pdf]

materials

Supplementary Materials

Detailed Inspection of γ-ray, Fast and Thermal Neutrons Shielding Competence of Calcium Oxide or Strontium Oxide Comprising Bismuth Borate Glasses

Gandham Lakshminarayana, Youssef Elmahroug, Ashok Kumar, Huseyin Ozan Tekin, Najeh Rekik, Mengge Dong, Dong-Eun Lee, Jonghun Yoon and Taejoon Park

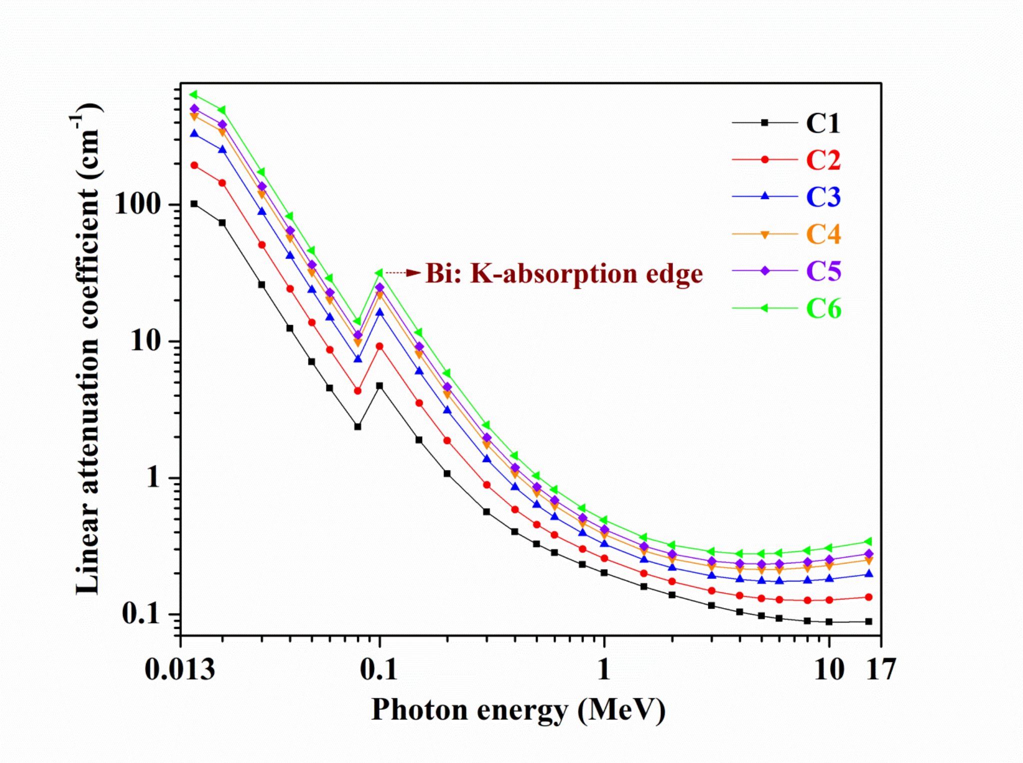

Figure S1. Variations of linear attenuation coefficient (μ, cm^{-1}) with photon energy (MeV) for all C1–C6 glasses.

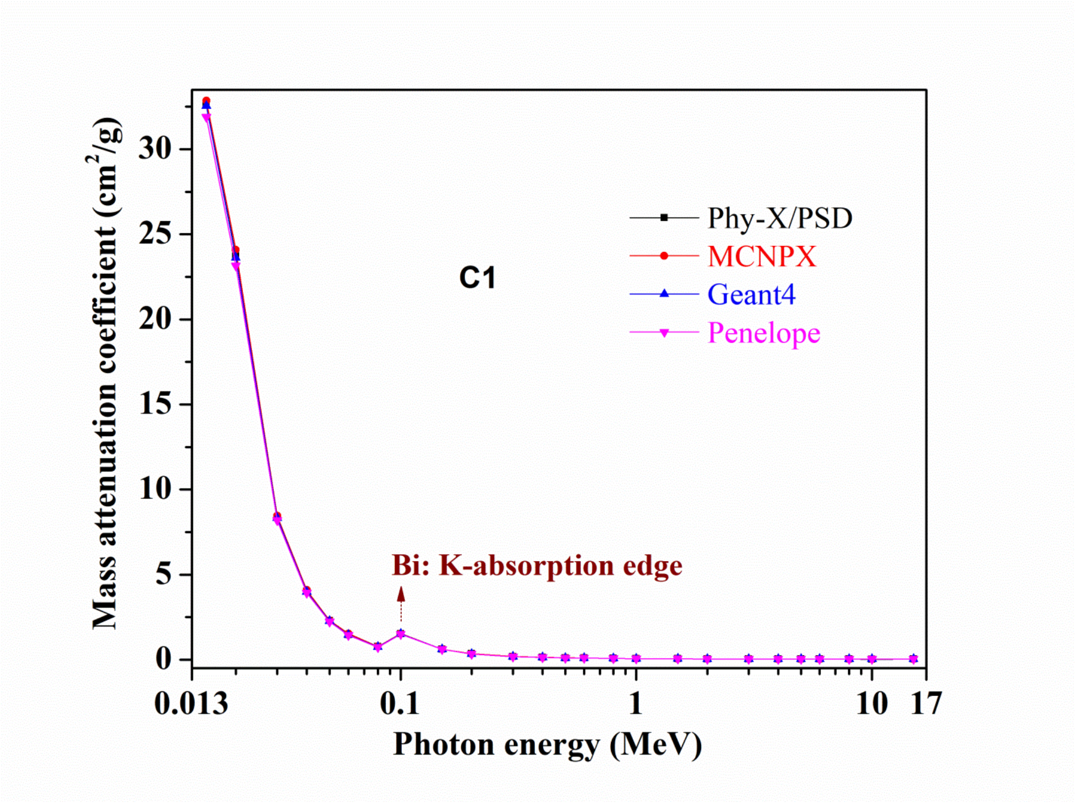

Mass attenuation coefficient (cm^2/g)
30
25
20
15
10
5
0
0.013
0.1
1
10
17
Photon energy (MeV)
C1
Phy-X/PSD
MCNPX
Geant4
Penelope
Bi: K-absorption edge

(a)

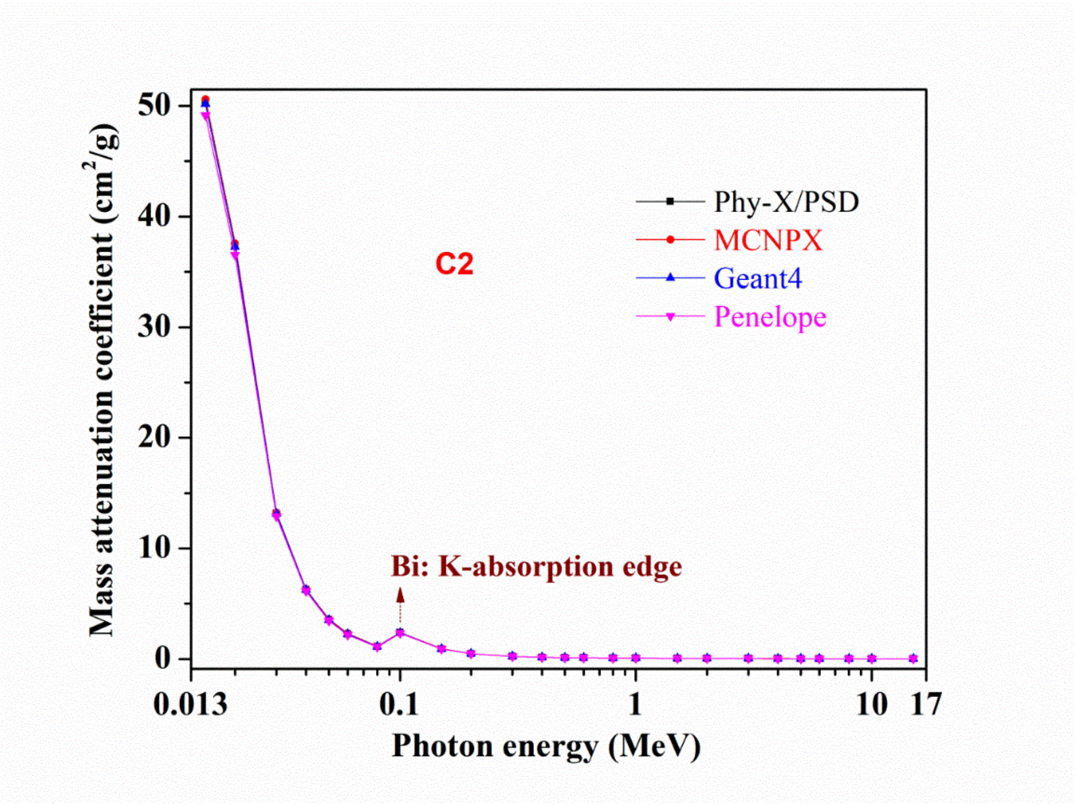

Mass attenuation coefficient (cm^2/g)
50
40
30
20
10
0
0.013
0.1
1
10
17
Photon energy (MeV)
C2
Phy-X/PSD
MCNPX
Geant4
Penelope
Bi: K-absorption edge

(b)

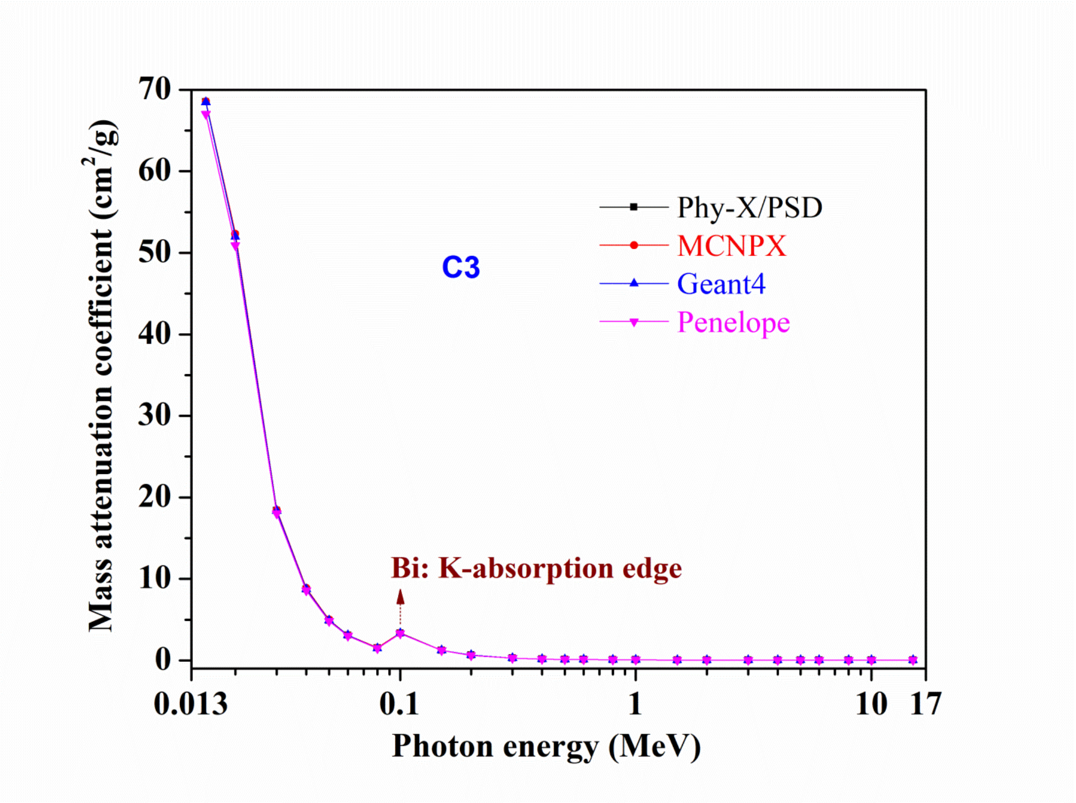

70
60
50
40
30
20
10
0
Mass attenuation coefficient (cm²/g)
C3
Phy-X/PSD
MCNPX
Geant4
Penelope
Bi: K-absorption edge
0.013
0.1
1
10
17
Photon energy (MeV)

(c)

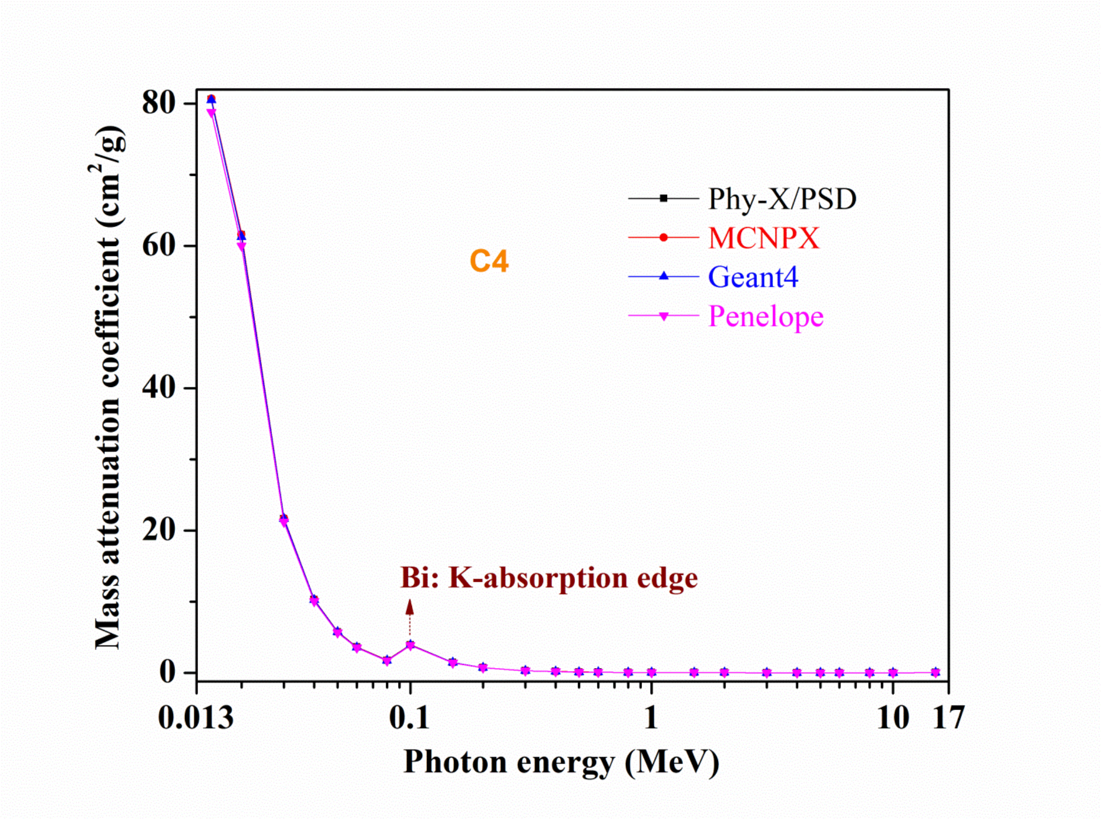

80
60
40
20
0
Mass attenuation coefficient (cm²/g)
C4
Phy-X/PSD
MCNPX
Geant4
Penelope
Bi: K-absorption edge
0.013
0.1
1
10
17
Photon energy (MeV)

(d)

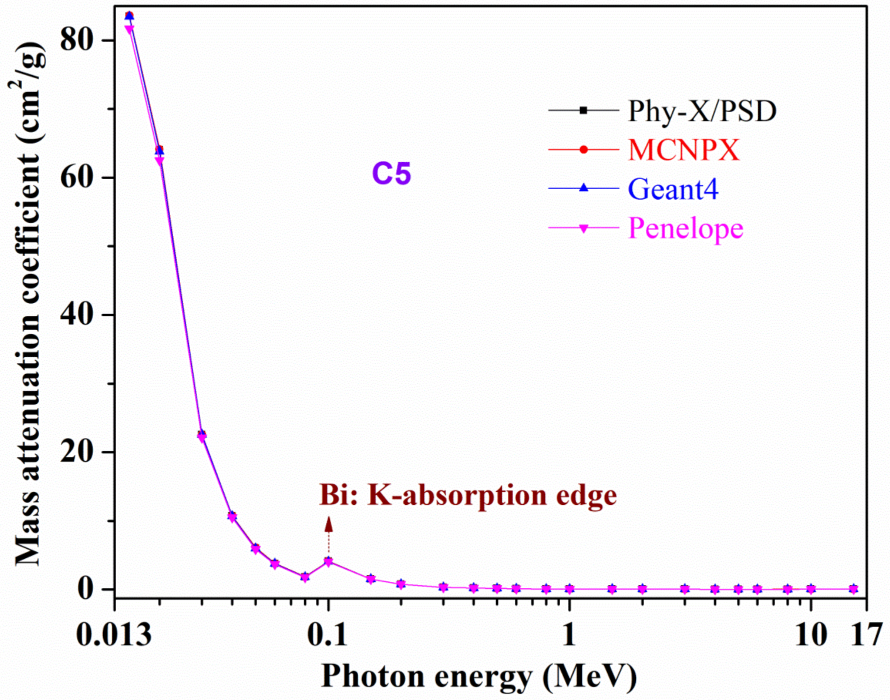
Mass attenuation coefficient (cm^2/g)
80
60
40
20
0
C5
Phy-X/PSD
MCNPX
Geant4
Penelope
Bi: K-absorption edge
0.013
0.1
1
10
17
Photon energy (MeV)

(e)

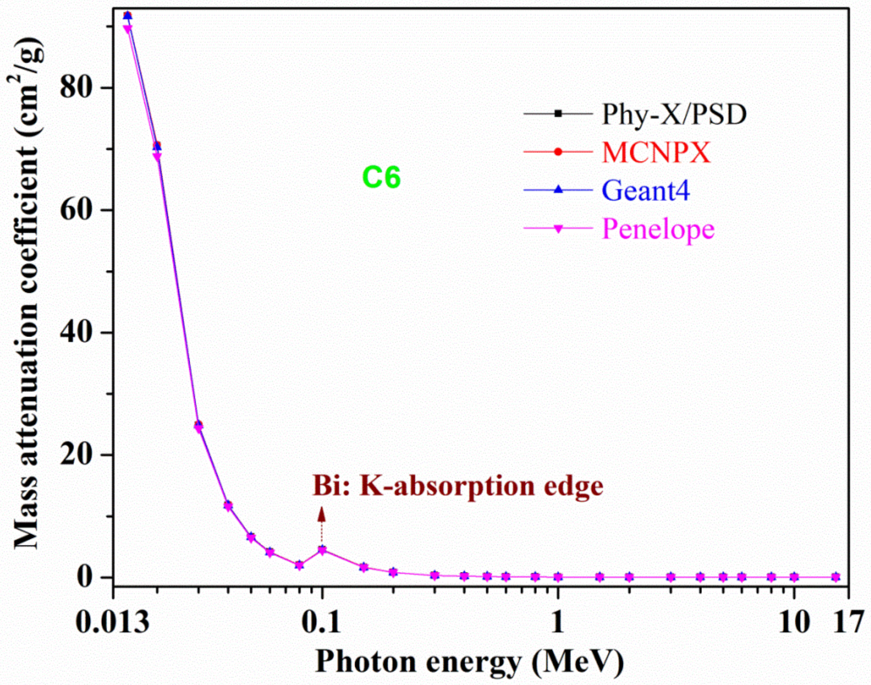
Mass attenuation coefficient (cm^2/g)
80
60
40
20
0
C6
Phy-X/PSD
MCNPX
Geant4
Penelope
Bi: K-absorption edge
0.013
0.1
1
10
17
Photon energy (MeV)

(f)

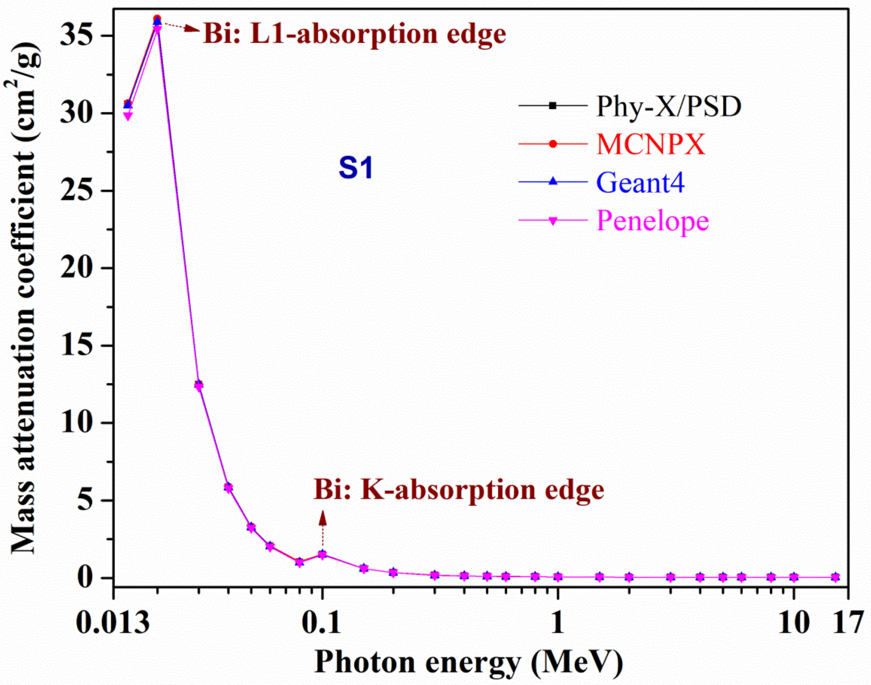

Bi: L1-absorption edge
S1
Phy-X/PSD
MCNPX
Geant4
Penelope
Bi: K-absorption edge
Mass attenuation coefficient (cm²/g)
35
30
25
20
15
10
5
0
0.013
0.1
1
10
17
Photon energy (MeV)

(g)

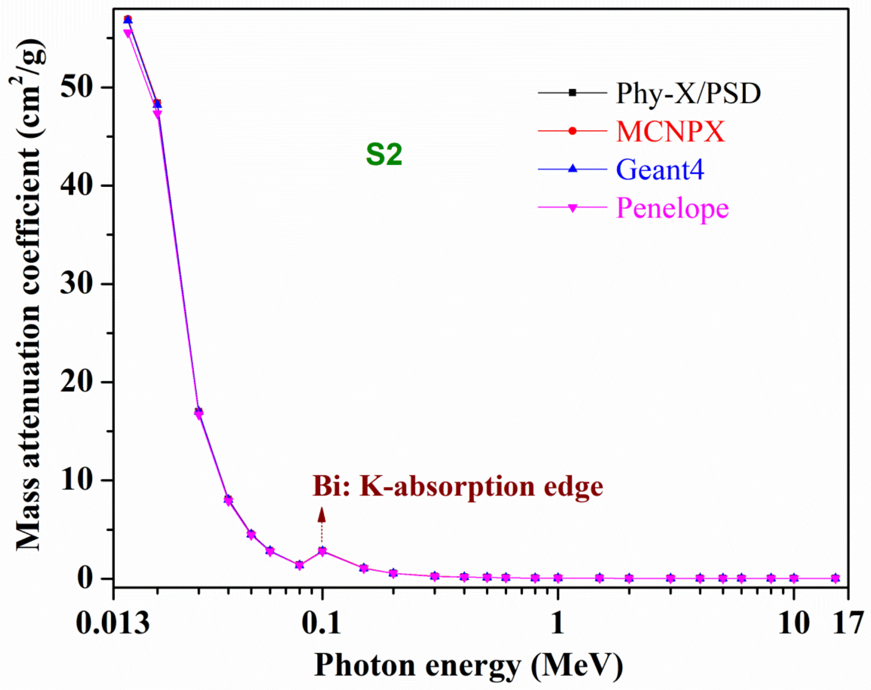

S2
Phy-X/PSD
MCNPX
Geant4
Penelope
Bi: K-absorption edge
Mass attenuation coefficient (cm²/g)
50
40
30
20
10
0
0.013
0.1
1
10
17
Photon energy (MeV)

(h)

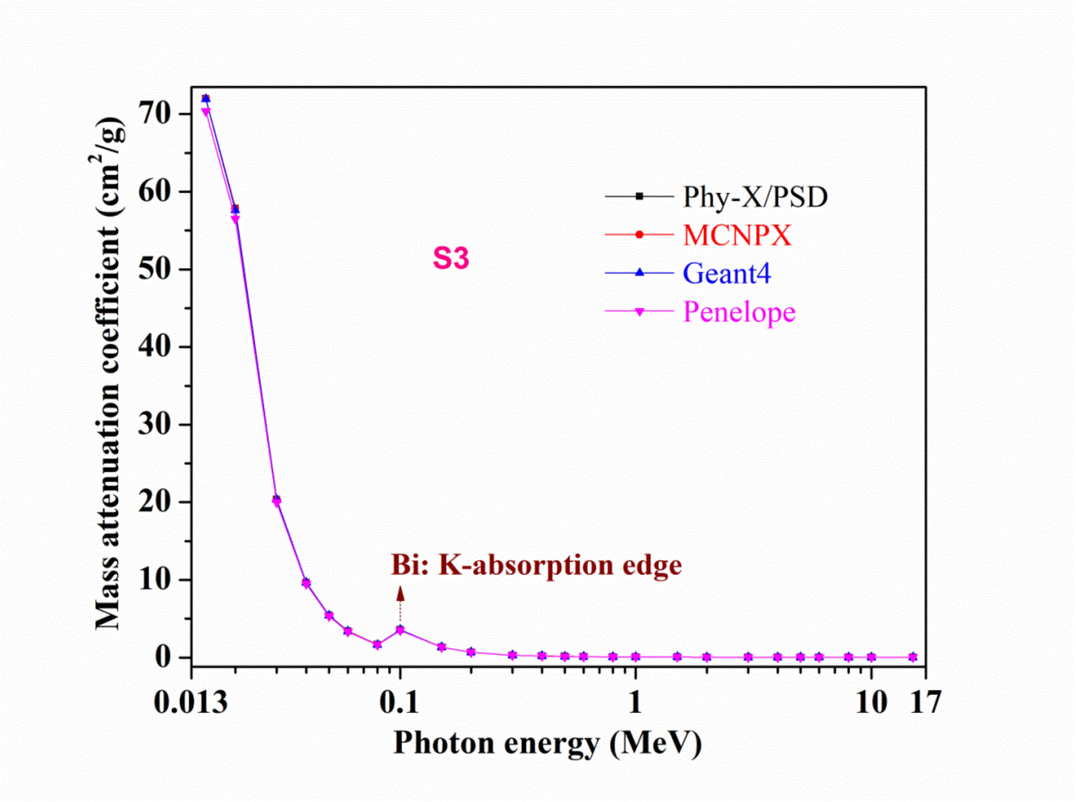
Mass attenuation coefficient (cm²/g)
Photon energy (MeV)
S3
Phy-X/PSD
MCNPX
Geant4
Penelope
Bi: K-absorption edge
0.013
0.1
1
10
17

(i)

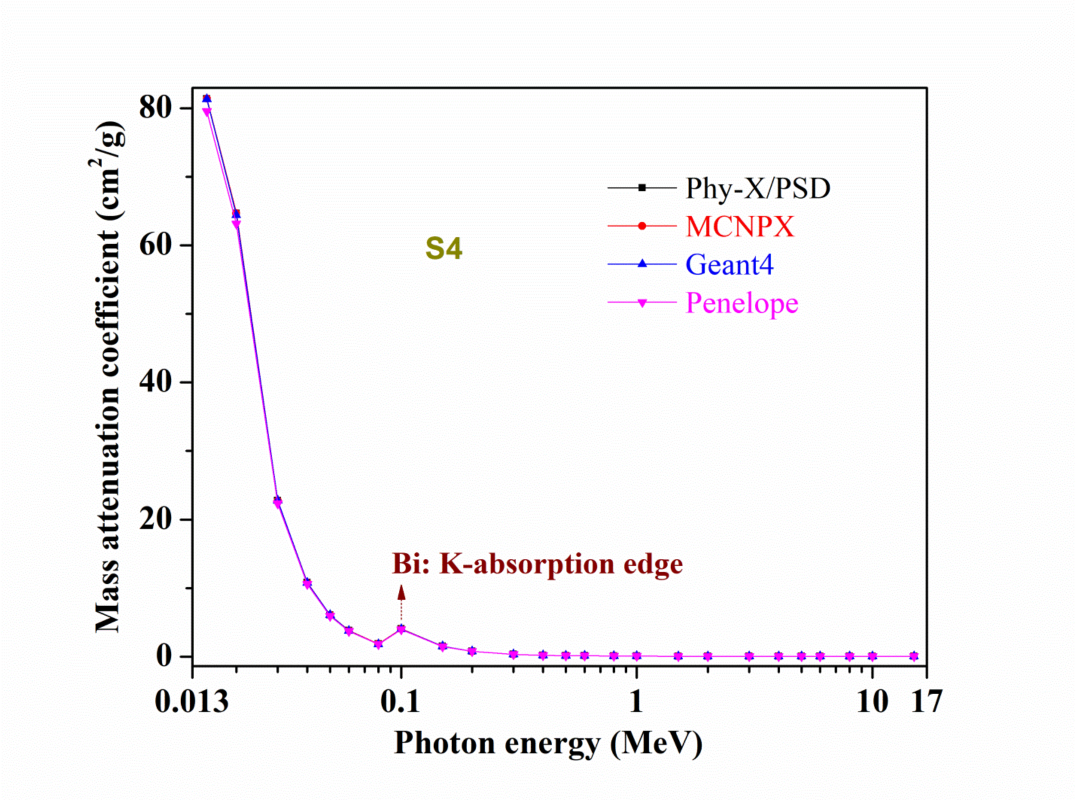
Mass attenuation coefficient (cm²/g)
Photon energy (MeV)
S4
Phy-X/PSD
MCNPX
Geant4
Penelope
Bi: K-absorption edge
0.013
0.1
1
10
17

(j)

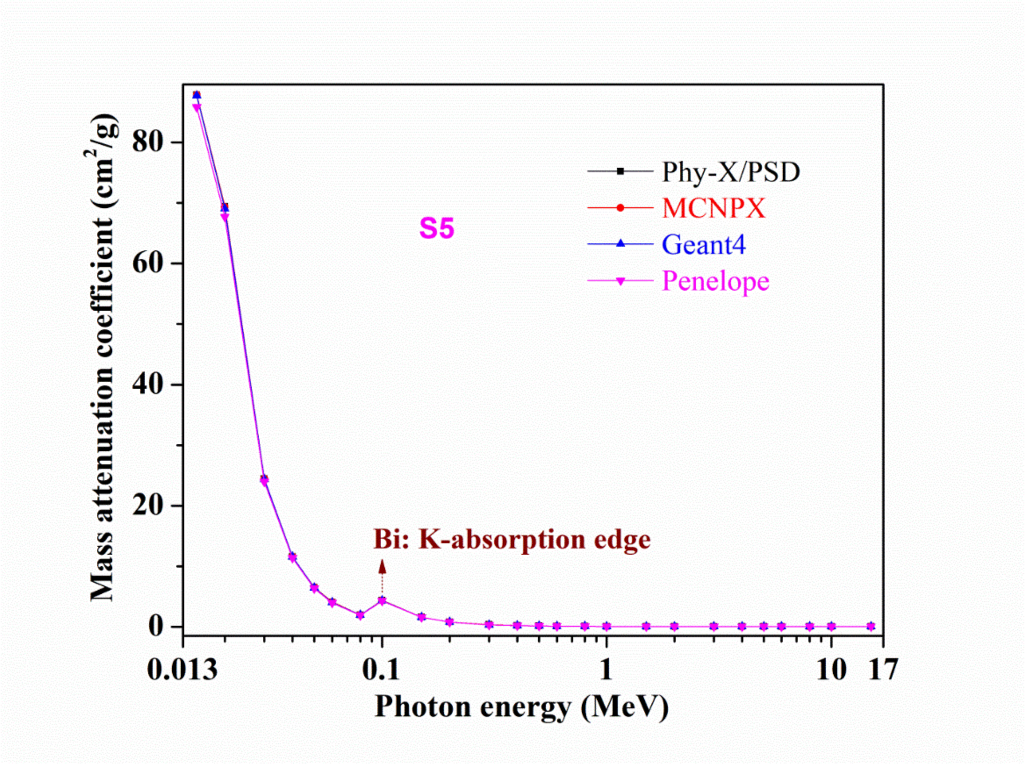

(k)

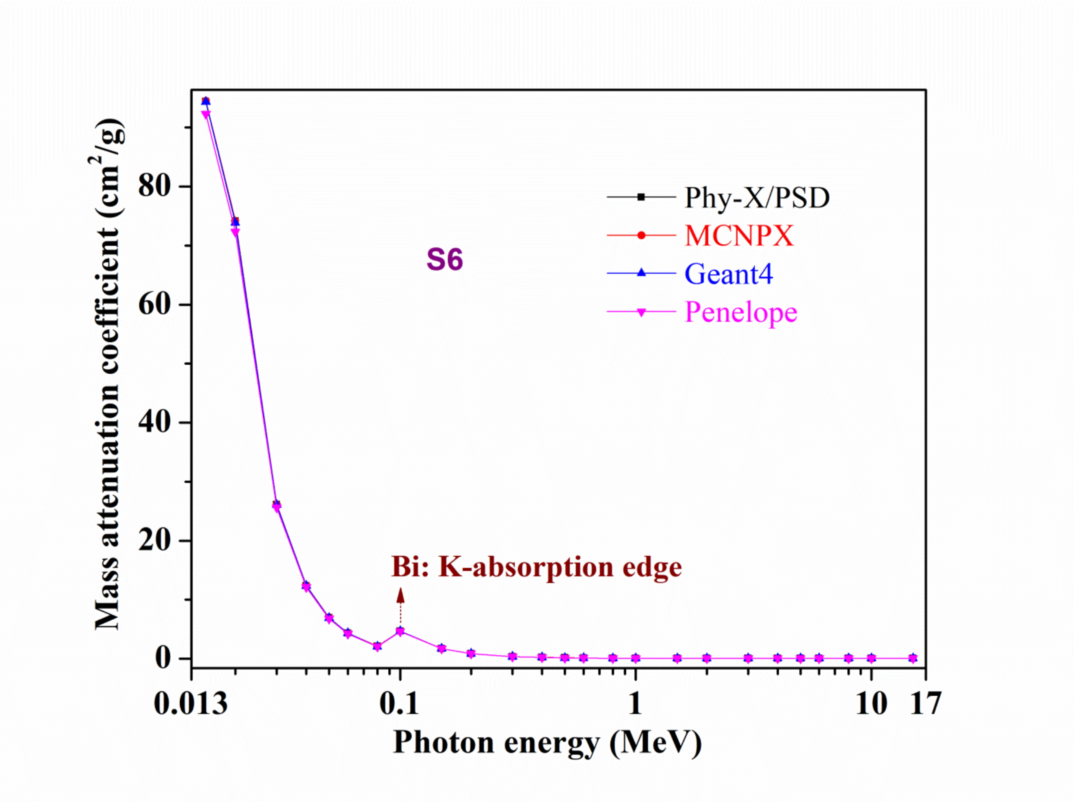

(l)

Figure S2. Comparison of Phy-X/PSD, MCNPX, Geant4, and Penelope code calculated mass attenuation coefficient versus photon energy for all (a-f) C1–C6 (g-l) S1–S6 glasses.

Z_{eff} changes for all C1–C6 and S1–S6 samples are displayed in Figs. S3 (a) and (b), individually. With greater Z element (*i.e*. Bi) addition instead of B/Ca and B/Sr elements from C1 to C6 and S1 to S6 glasses, Z_{eff} values are enhanced and stick to an akin movement with incident photon energy. So the inclusion of the heavy metal oxide, Bi_2O_3, to the glass improves the interactions between 'Bi' atoms and photons, leading to a less number of γ-rays to escape through it. Moreover, in all chosen glasses, within 15 KeV–15 MeV energy range the S6 sample owns relatively higher Z_{eff} due to its larger content of 'Bi' (=80.6813 wt%), for instance, 80.17 (at 0.015 MeV energy) and 51.12 (at 15 MeV energy). The deduced Z_{eff} for all C1, C2, C3, C4, C5, and C6, and S1, S2, S3, S4, S5, and S6 glasses at 15 KeV energy is 51.18, 61.15, 71.78, 74.5, 76.81, and 79.08, and 56.69, 71.87, 76.27, 78.12, 79.2, and 80.17 accordingly. Initially, Z_{eff} values are greater at the lowest γ-ray energies, *i.e.* 20 KeV for all C1–C6 samples and 15 KeV for all S1–S6 glasses, and after, they rapidly reduce up to 0.4 MeV energy as PEA ($\propto Z^{4-5}$) process command this range, apart from a sudden hike in Z_{eff} at 0.1 MeV energy (because of 'Bi' K-absorption edge). For all C1–C6 glasses, at 0.4 MeV energy, corresponding Z_{eff} quantities are 13.03, 18.43, 25.73, 33.62, 35.1, and 42.3, and at the same energy, these are 14.95, 21.78, 28.32, 34.61, 40.08, and 47.07, respectively, for all S1–S6 samples. Then, from 0.5 MeV up to 1.5 MeV energy, for all C1–C6 and S1–S6 glasses, Z_{eff} decrements and/or changes are minor, approaching the minimal at 1.5 MeV, due to CS ($\propto Z$) action dominance. At 1.5 MeV energy, for all C1–C6 and S1–S6 samples obtained Z_{eff} quantities are 9.974, 12.64, 16.27, 21.13, 21.94, and 27.07, and 11.62, 14.32, 17.77, 21.71, 25.55, and 31.16, correspondingly. Thereafter, within 2–15 MeV γ-ray energy range, owing to PP ($\propto Z^2$) mechanism supremacy, Z_{eff} is moderately increased for all C1–C6 and S1–S6 glasses. For instance, 10.04, 12.76, 16.47, 21.41, 22.24, and 27.44, and 14.67, 20.91, 29.2, 37.44, 39.21, and 46.54, respectively, are the Z_{eff} values for all C1–C6 samples at 2 and 15 MeV energies, while they are, at the same energies, 11.73, 14.49, 18, 22, 25.9, and 31.57, and 18.02, 25.27, 32.36, 38.92, 44.4, and 51.12, accordingly, for all S1–S6 glasses. Here, as it contains bigger Z_{eff} to more efficiently interact with incident photons, in all selected samples, S6 glass is most beneficial for γ-ray attenuation.

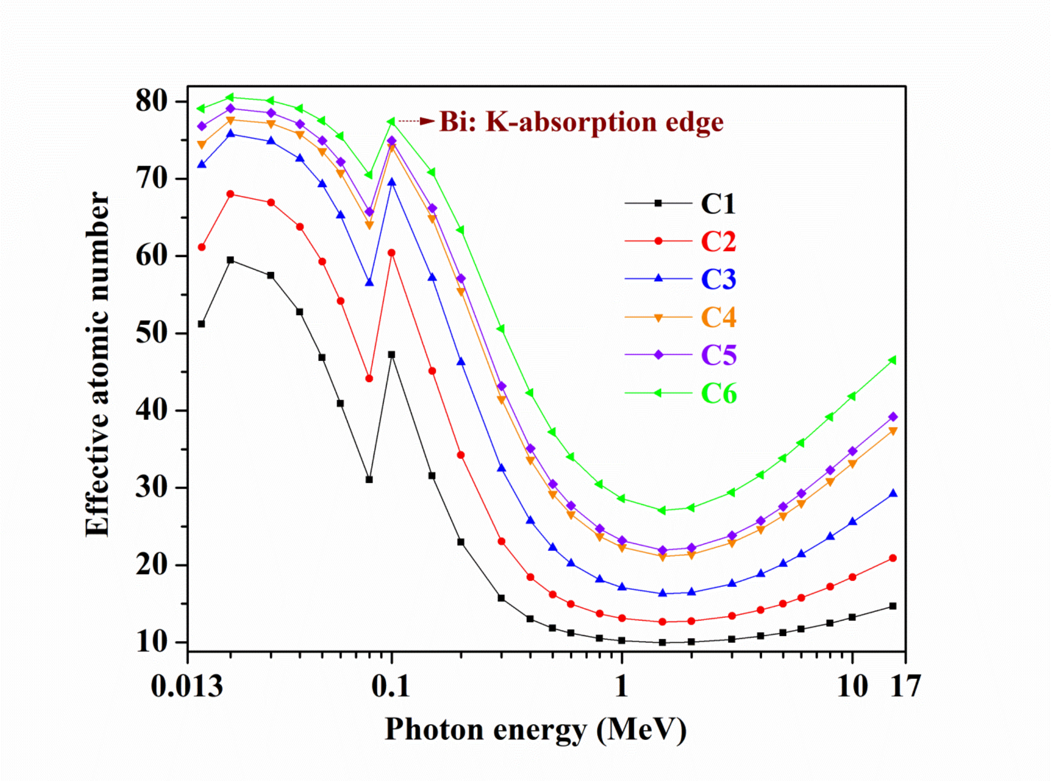

(a)

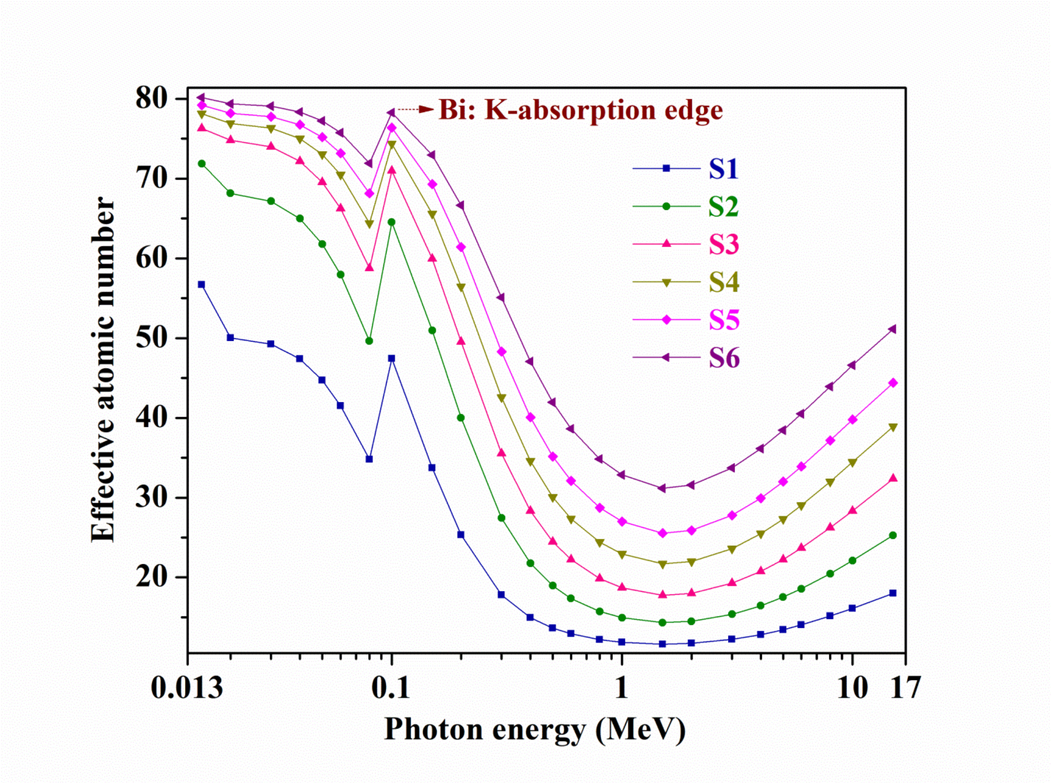

(b)

Figure S3. Variations of effective atomic number (Z_{eff}) with photon energy (MeV) for all (a) C1–C6 (b) S1–S6 glasses.

Figs. S4 (a) and (b) presents N_{eff} deviations for all C1–C6 and S1–S6 glasses, respectively. For all studied glasses, derived N_{eff} quantities swing in an alike fashion to that of Z_{eff} fluctuations at all chosen photon energies and can be elucidated principally by respective PEA, CS, and PP processes preeminence at 0.015 KeV–0.4 MeV (lower), 0.5–1.5 MeV (intermediate), and 2–15 MeV (high) energy regions. For exploring a material photon attenuation capacity, evaluation of its' Z_{eff} and N_{eff} is essential and commonly, greater N_{eff} represents higher interactions between photons and electrons and energy transfer probabilities. Among all C1–C6 and S1–S6 glasses, sample C1 (holds 70 mol% B_2O_3 and 46.4821 wt% 'O') has the largest N_{eff} at the lowest photon energies (0.015–0.05 MeV). For instance, at 0.05 MeV energy, from C1 to C6 glass, the computed N_{eff} values are 13.94×10^{23}, 13.92×10^{23}, 12.56×10^{23}, 10.17×10^{23}, 9.953×10^{23}, and 8.235×10^{23} electrons/g, accordingly, while at the same energy, from S1 to S6 sample, they are 11.08×10^{23}, 12.62×10^{23}, 11.43×10^{23}, 9.761×10^{23}, 8.465×10^{23}, and 7.032×10^{23} electrons/g, respectively. Further, among all S1–S6 glasses, sample S2 (contains 70 mol% B_2O_3 and 32.1965 wt% 'O') has higher N_{eff} within 0.015–0.2 MeV energy range, for example, at 0.2 MeV energy, 6.279×10^{23}, 8.17×10^{23}, 8.146×10^{23}, 7.547×10^{23}, 6.914×10^{23}, and 6.069×10^{23} electrons/g, individually, are the N_{eff} quantities for S1 to S6 sample. Likewise, respective minimal N_{eff} values achieved for all C1–C6 and S1–S6 samples at 1.5 MeV energy are 2.967×10^{23}, 2.966×10^{23}, 2.949×10^{23}, 2.922×10^{23}, 2.914×10^{23}, and 2.876×10^{23} electrons/g, and 2.881×10^{23}, 2.924×10^{23}, 2.922×10^{23}, 2.901×10^{23}, 2.877×10^{23}, and 2.837×10^{23} electrons/g. Next, for C1 to C6 and S1 to S6 glasses, at 15 MeV photon energy, the obtained N_{eff} quantities are 4.364×10^{23}, 4.908×10^{23}, 5.293×10^{23}, 5.175×10^{23}, 5.208×10^{23}, and 4.944×10^{23} electrons/g, and 4.466×10^{23}, 5.16×10^{23}, 5.322×10^{23}, 5.203×10^{23}, 4.999×10^{23}, and 4.655×10^{23} electrons/g, accordingly. Overall, within selected γ-ray energy range, N_{eff} has varied at 15.22×10^{23} – 4.364×10^{23} electrons/g and 14.68×10^{23} – 5.16×10^{23} electrons/g boundaries, for C1 and S2 samples, accordingly.

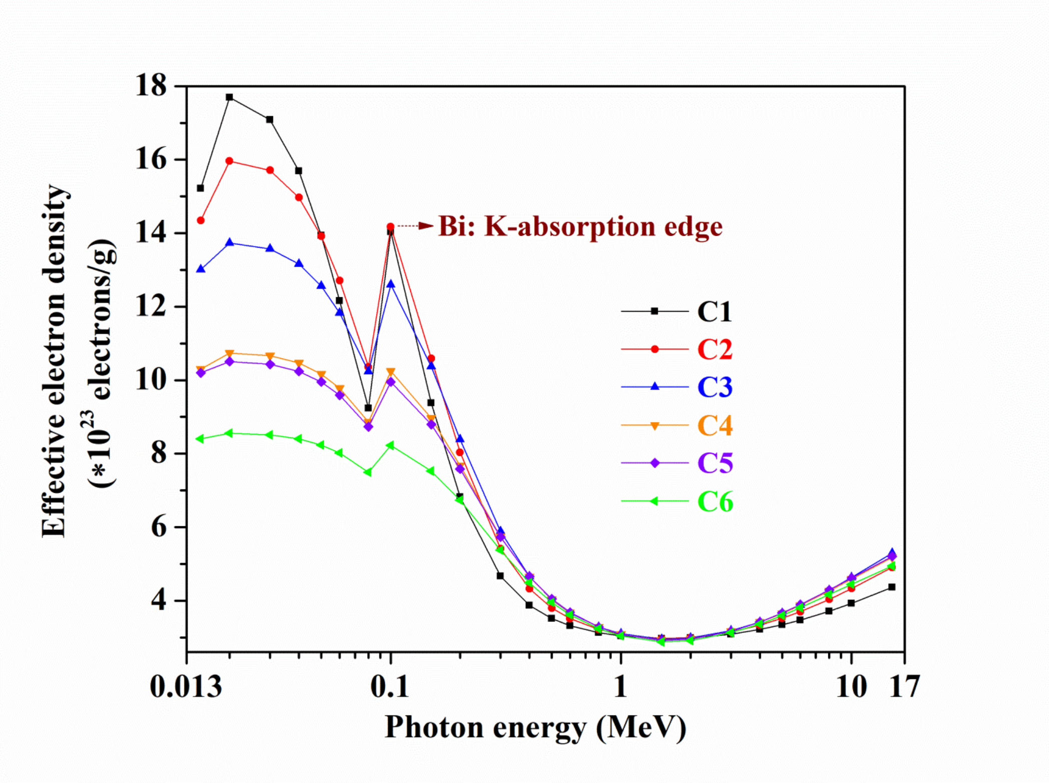

(a)

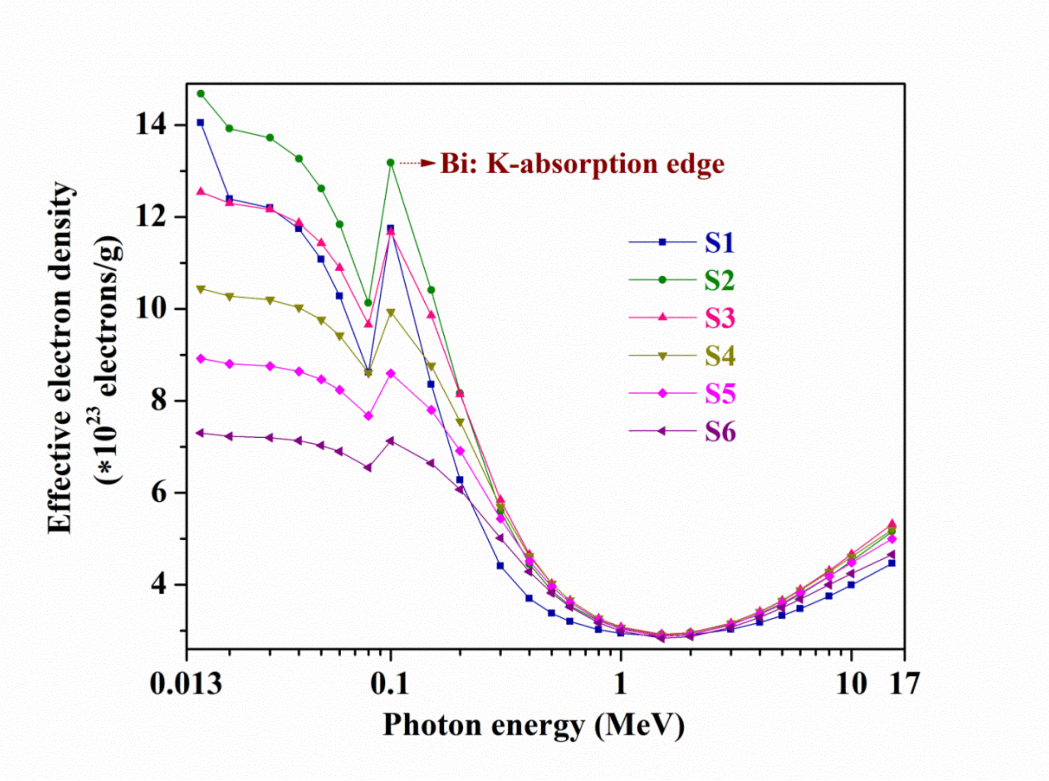

(b)

Figure S4. Variations of effective electron density (N_{eff}) with photon energy (MeV) for all (a) C1–C6 (b) S1–S6 glasses.

Usually, shielding substances thickness (in cm) is figured out by assessing HVL, TVL, and MFP (banks on substance's chemical balance, photon energy, 'μ', and ρ) and here, the lesser the HVL, TVL, and MFP, the lower the thickness requirements for avoiding larger space occupations by them at the nuclear medicine and nuclear reactor facilities for preferable γ-ray shielding. Discrepancies in estimated HVL and TVL values for all C1–C6 and S1–S6 samples are depicted in Figs. S5 (a) and (b) and Figs. S6 (a) and (b), accordingly, and respective inset plots show expanded 0.029–0.16 MeV energy regions. For all examined glasses, at all considered photon energies, calculated HVL and TVL drifts are the same (see Figs. S5 and S6). Here, HVL and TVL decrease in the C6<C5<C4<C3<C2<C1 and S6<S5<S4<S3<S2<S1 orders, respectively. As one can see, for all C1–C6 and S1–S6 samples, at the 0.015–0.1 MeV energies, HVL and TVL quantities are minimal exhibiting fewer changes. At 0.015 and 0.1 MeV energies, HVL and TVL calculated from C1 to C6 glass are (0.007, 0.004, 0.002, 0.002, 0.001, and 0.001 cm, and 0.023, 0.012, 0.007, 0.005, 0.005, and 0.004 cm), and (0.147, 0.075, 0.043, 0.032, 0.028, and 0.022 cm, and 0.488, 0.25, 0.143, 0.105, 0.092, and 0.073 cm), respectively. Similarly, (0.006, 0.003, 0.002, 0.001, 0.001, and 0.001 cm, and 0.021, 0.009, 0.006, 0.005, 0.004, and 0.003 cm), and (0.129, 0.054, 0.038, 0.028, 0.024, and 0.02 cm, and 0.429, 0.18, 0.125, 0.095, 0.079, and 0.065 cm), accordingly, are HVL and TVL from S1 to S6 sample at 0.015 and 0.1 MeV energies. Next, with rising photon energy from 0.15 MeV, HVL and TVL of all C1–C6 and S1–S6 glasses steeply grow, attaining maximal at 10, 8, and 6 MeV for respective C1 (=7.889 cm and 26.21 cm), C2 (=5.479 cm and 18.2 cm), and C3 (=3.963 cm and 13.17 cm) glasses, and 5 MeV for C4 (=3.248 cm and 10.79 cm), C5 (=2.962 cm and 9.841 cm), and C6 (=2.49 cm and 8.271 cm) samples, and accordingly, at 10 MeV energy for S1 (=6.453 cm and 21.43 cm) glass, 6 MeV for S2 (=4.422 cm and 14.69 cm) and S3 (=3.636 cm and 12.08 cm) samples, 5 MeV for S4 (=2.991 cm and 9.935 cm) and S5 (=2.638 cm and 8.764 cm) glasses, and 4 MeV for S6 glass (=2.27 cm and 7.542 cm). Then, from energies above 10 MeV for C1 and S1 glasses, beyond 8 MeV for C2 sample, after 6 MeV for C3, S2, and S3 glasses, above 5 MeV for C4, C5, C6, S4, and S5 samples, and after 4 MeV for S6 glass, HVL and TVL are slightly depleted. For instance, at 15 MeV energy, corresponding HVL and TVL for all C1–C6 and S1–S6 samples are (7.835, 5.18, 3.517, 2.755, 2.485, and 2.026 cm, and 26.03, 17.21, 11.68, 9.153, 8.254, and 6.731 cm), and (6.256, 4.033, 3.159, 2.509, 2.163, and 1.819 cm, and 20.78, 13.4, 10.49, 8.335,7.187, and 6.044 cm). Correspondingly as narrated for μ, μ/ϱ, Z_{eff}, and N_{eff} deviations at distinctive energy regions for all C1–C6 and S1–S6 glasses, both HVL and TVL alterations also can be explicated on account of separate PEA, CS, and PP events ascendancy. Among all selected C1–C6 and S1–S6 samples, relatively, glass S6 has the smaller HVL and TVL at all inspected energies as it owns proportionally bigger 'μ' and 'ρ' (=7.59 g/cm^3), implying its' better photon shielding effectiveness in all samples (see Figs. S5 and S6).

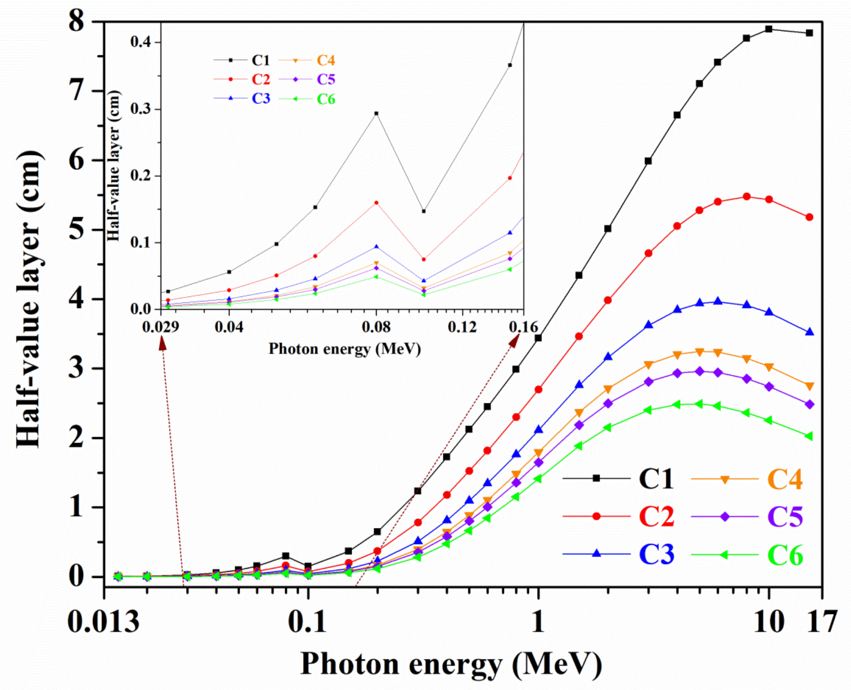

(a)

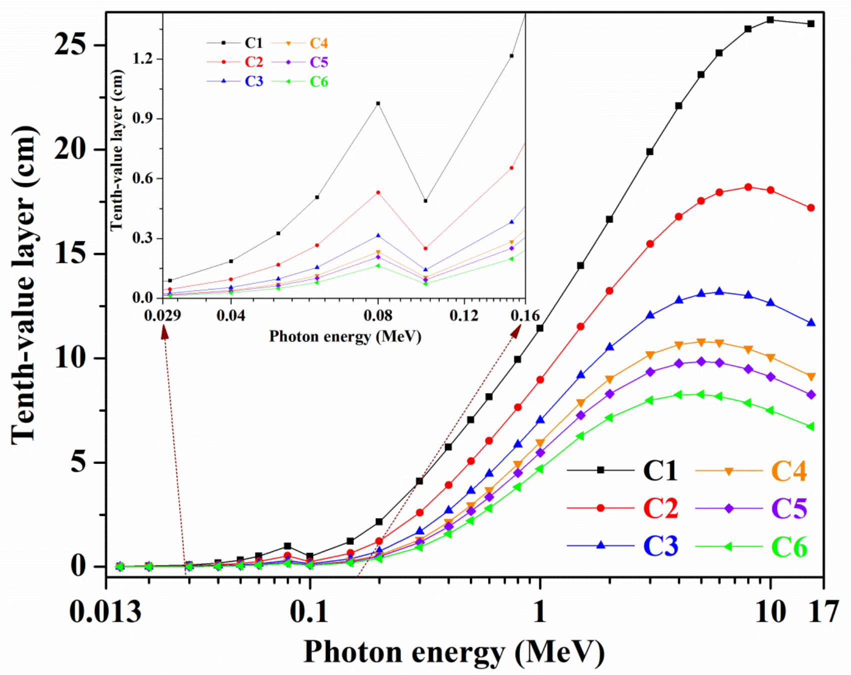

(b)

Figure S5. Variations of (a) half-value layer (HVL) (inset, within the 0.029–0.16 MeV photon energy range) and (b) tenth-value layer (TVL) (inset, within the range of 0.029–0.16 MeV photon energy) with photon energy for all C1–C6 glasses.

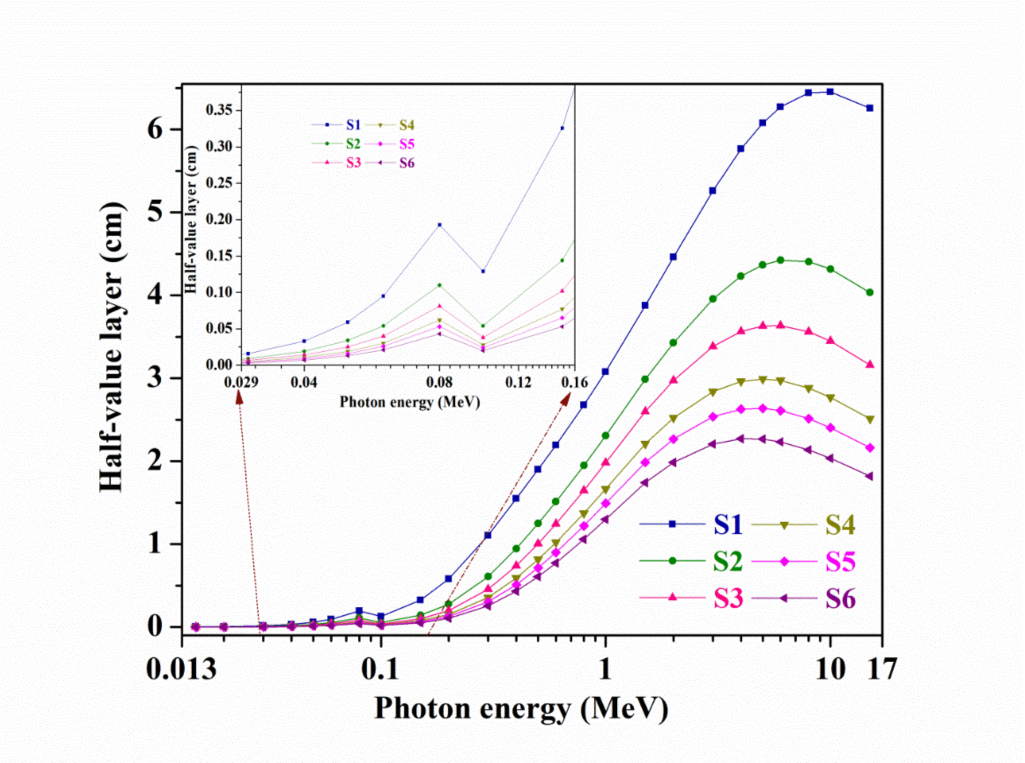

(a)

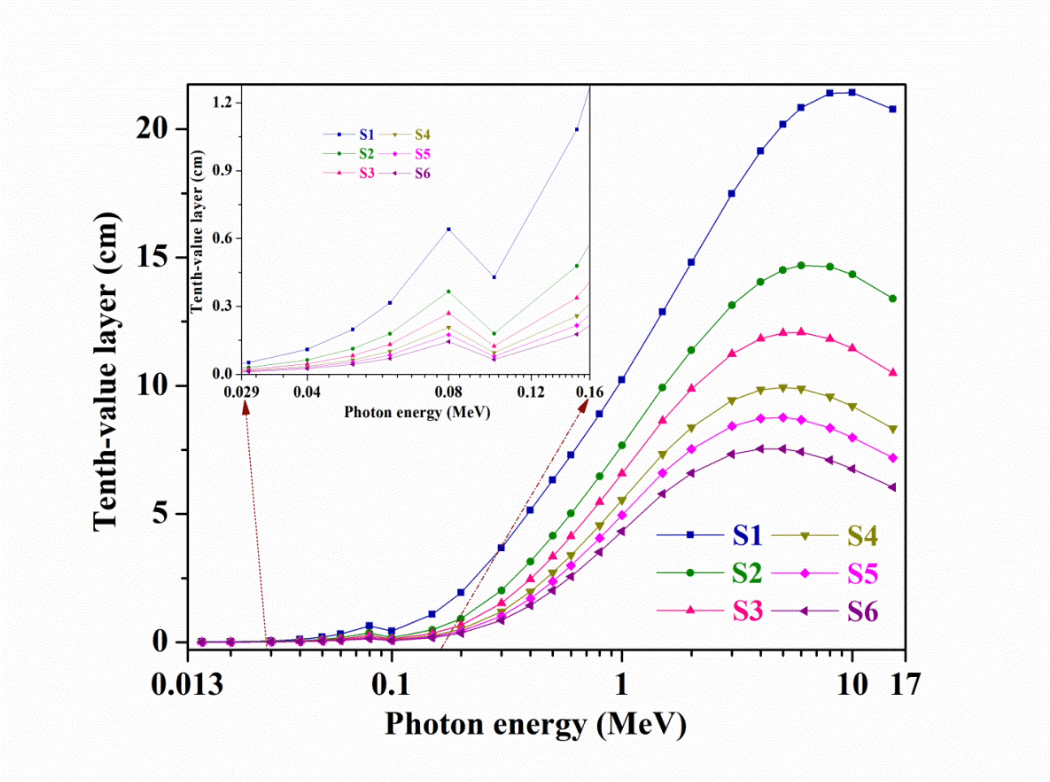

(b)

Figure S6. Variations of (a) half-value layer (HVL) (inset, within the 0.029–0.16 MeV photon energy range) and (b) tenth-value layer (TVL) (inset, within the range of 0.029–0.16 MeV photon energy) with photon energy for all S1–S6 glasses.

Figs. S7 (a) and (b) exhibits the alterations in deduced MFP values for all respective C1–C6 and S1–S6 glasses, whereas corresponding inset plots display 0.029–0.16 MeV energy zoom-in regions. Correspondingly to the HVL and TVL, for all selected glasses, derived MFP quantities also emulate a similar direction with photon energy. On behalf of B_2O_3/CaO or B_2O_3/SrO, the increasing insertion of Bi_2O_3 content from C1 to C6 and S1 to S6 glasses steadily lowered the MFP at any discrete γ-ray energy as sample's 'ρ' enhances (see Tables 1 and 2 in the main text). For all C1–C6 and S1–S6 glasses, at inferior photon energies (*i.e.* 0.015–0.1 MeV range), calculated MFP (=1/μ) values are minimal with small variations. At 15 KeV and 0.1 MeV energies, (0.01, 0.005, 0.003, 0.002, 0.002, and 0.002 cm) and (0.212, 0.109, 0.062, 0.045, 0.04, and 0.032 cm), and (0.009, 0.004, 0.003, 0.002, 0.002, and 0.001 cm) and (0.186, 0.078, 0.054, 0.041, 0.034, and 0.028 cm) are the respective obtained MFP quantities for all C1, C2, C3, C4, C5, and C6, and S1, S2, S3, S4, S5, and S6 glasses. Then, considerable accruals in MFP beyond 0.15 MeV energy up to 10 MeV for C1 (=11.38 cm), S1 (=9.309 cm) samples, 8 MeV for C2 glass (=7.904 cm), 6 MeV for C3 (=5.718 cm), S2 (=6.379 cm), and S3 (=5.246 cm) glasses, 5 MeV for C4 (=4.686 cm), C5 (=4.274 cm), C6 (=3.592 cm), S4 (=4.315 cm), and S5 (=3.806 cm) samples, and 4 MeV for S6 glass (=3.276 cm) are observed. Later, after 10 MeV for C1 and S1 glasses, beyond 8 MeV for C2 sample, above 6 MeV for C3, S2, and S3 samples, beyond 5 MeV for C4, C5, C6, S4, and S5 glasses, after 4 MeV for S6 sample up to 15 MeV energy, MFP quantities are slowly declined. At 15 MeV energy, derived MFP for corresponding all C1–C6 and S1–S6 samples are (11.3, 7.473, 5.074, 3.975, 3.585, and 2.923 cm) and (9.026 , 5.818, 4.558, 3.62, 3.121, and 2.625 cm). Within 15 KeV–15 MeV energy range, for MFP changes also, at typical γ-ray energy ranges (*i.e.* low, medium, and high), as made clear in cases of μ, μ/ϱ, Z_{eff}, N_{eff}, and HVL and TVL variations for all C1–C6 and S1–S6 samples, appropriate PEA, CS, and PP mechanisms play pivotal roles. In all studied glasses, correlatively, sample S6 owns the minimal MFP (larger 'ρ', bigger μ/ϱ, and lesser HVL and TVL), signifying its' better potential for γ-ray shielding in all samples.

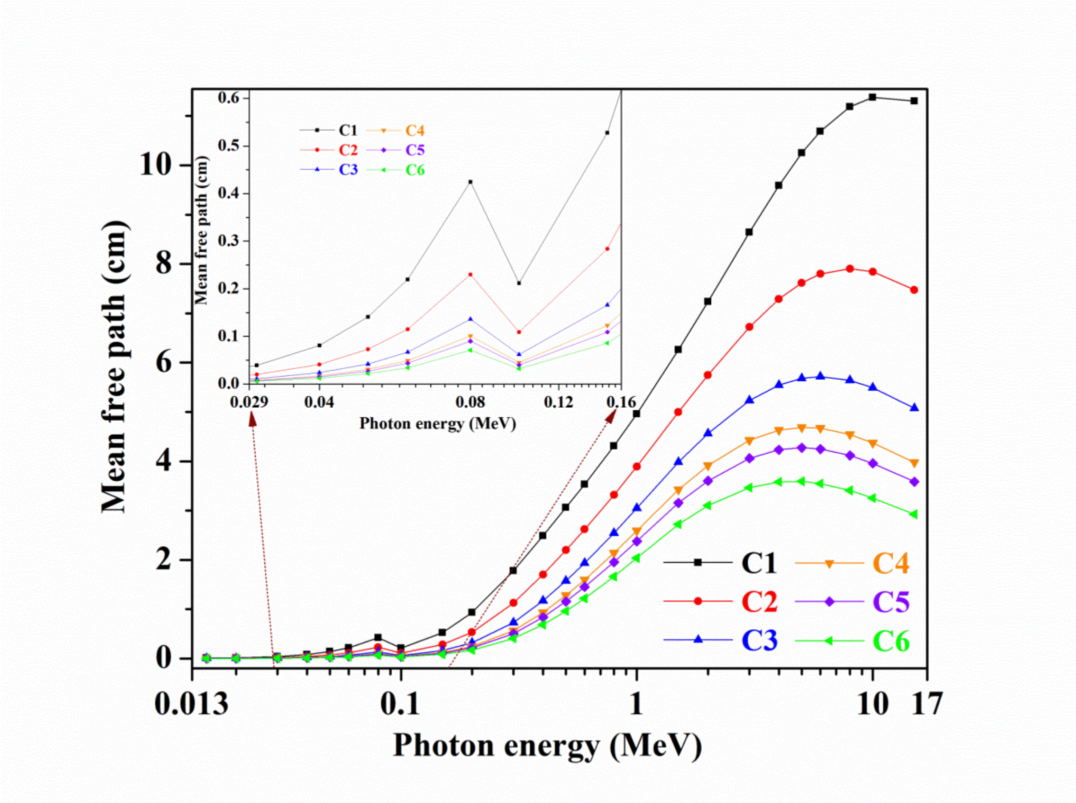

(a)

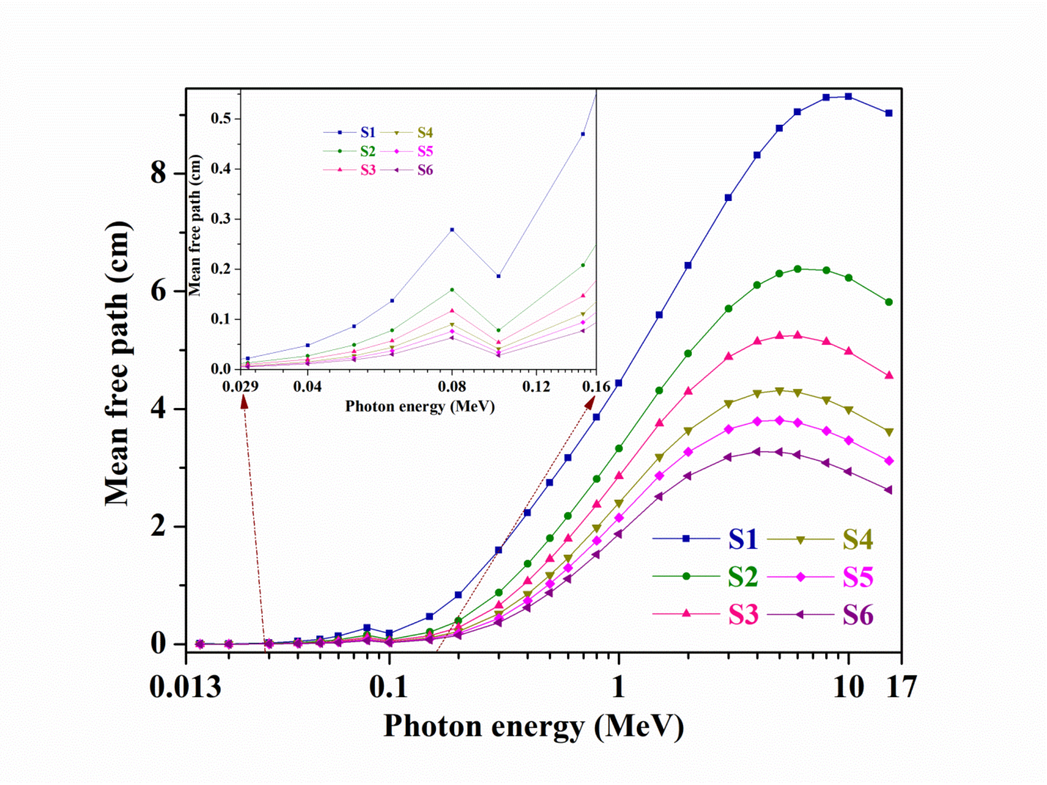

(b)

Figure S7. Variations of mean free path (MFP) with photon energy (MeV) for all (a) C1–C6 (inset, within the range of 0.029–0.16 MeV photon energy) and (b) S1–S6 (inset, within the 0.029–0.16 MeV photon energy range) glasses.

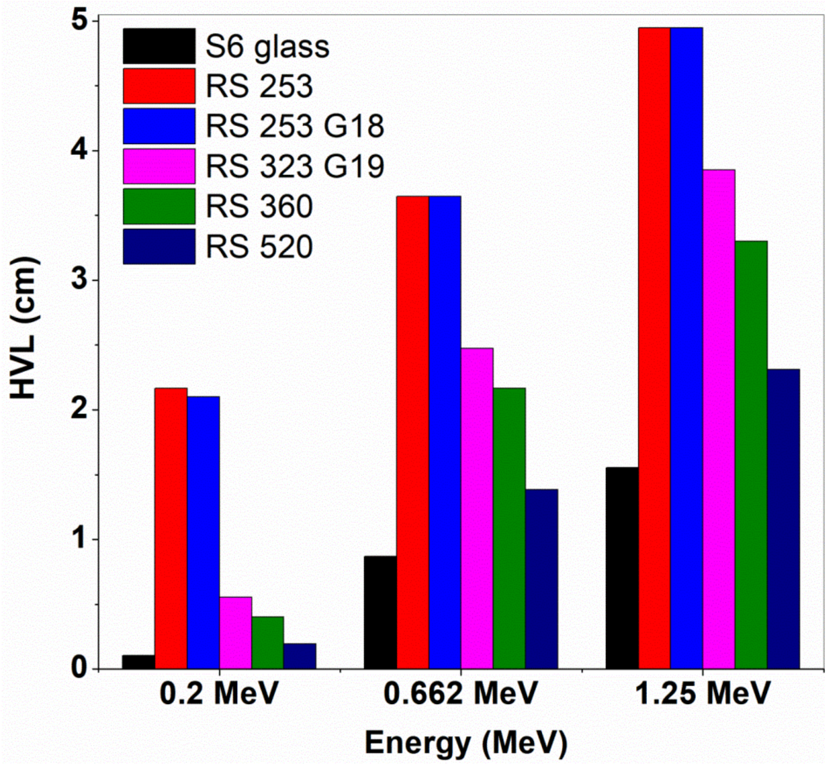

Figure S8. Comparison of HVL of the glass 'S6' with some commercial glasses.

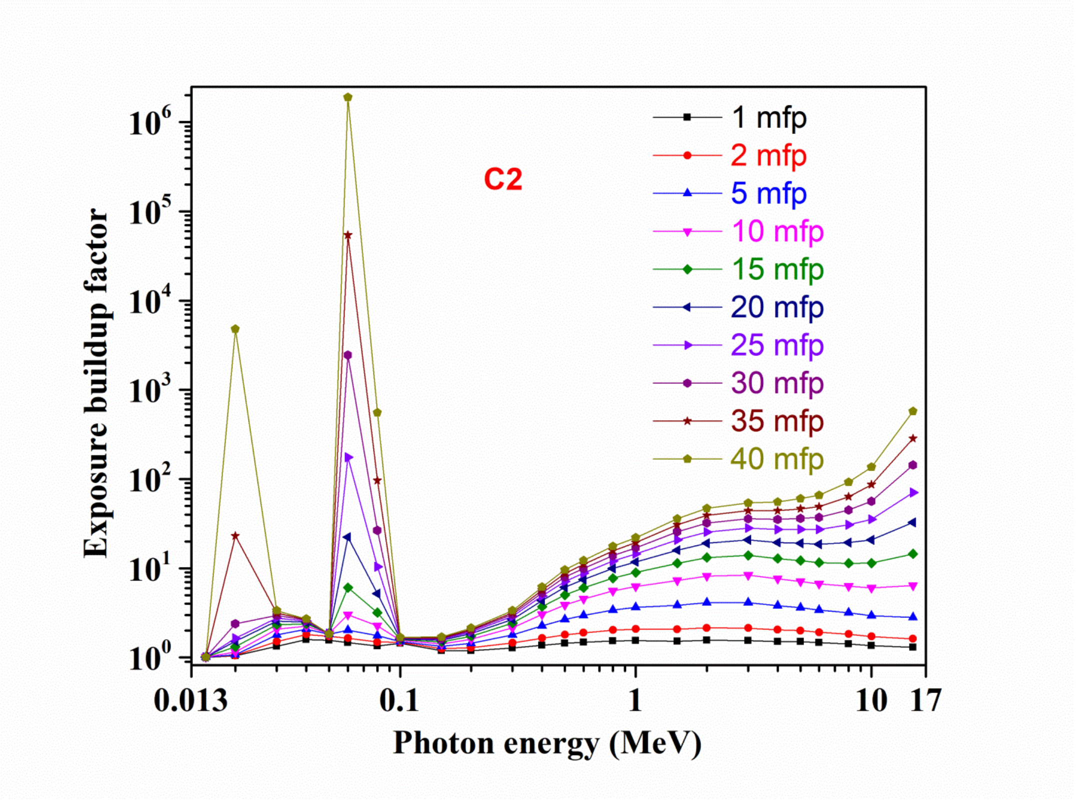
C2
1 mfp
2 mfp
5 mfp
10 mfp
15 mfp
20 mfp
25 mfp
30 mfp
35 mfp
40 mfp
Exposure buildup factor
Photon energy (MeV)
0.013
0.1
1
10
17

(a)

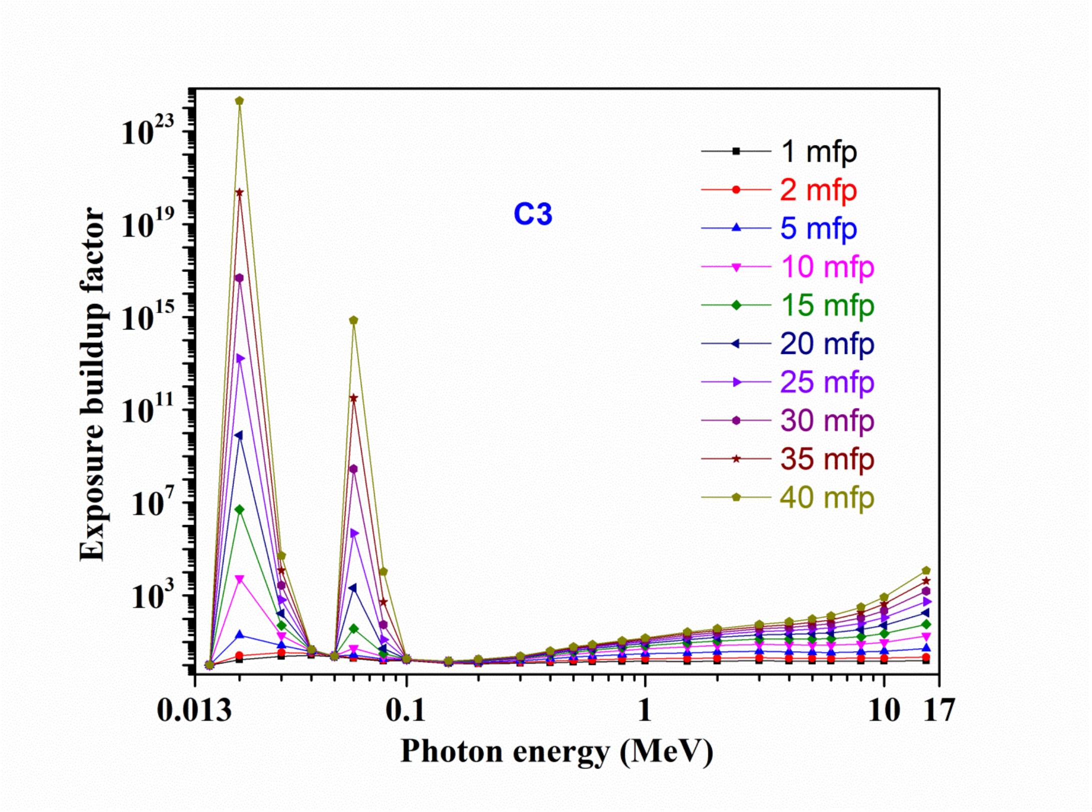
C3
1 mfp
2 mfp
5 mfp
10 mfp
15 mfp
20 mfp
25 mfp
30 mfp
35 mfp
40 mfp
Exposure buildup factor
Photon energy (MeV)
0.013
0.1
1
10
17

(b)

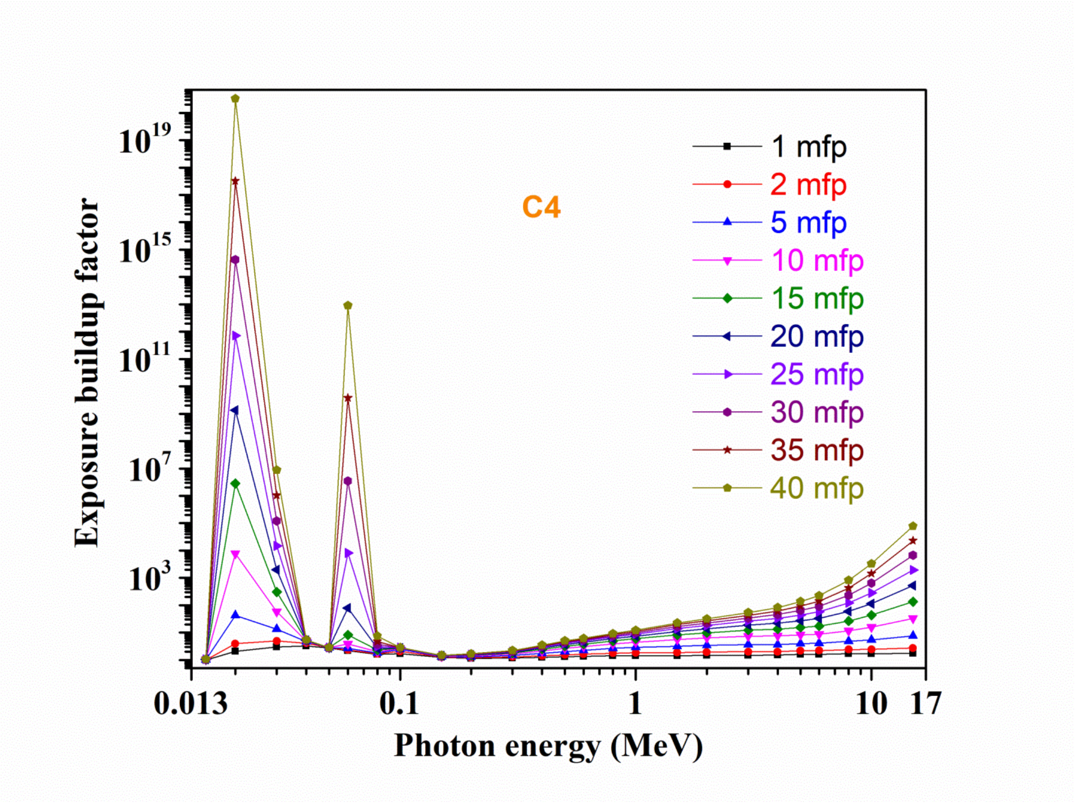
C4
1 mfp
2 mfp
5 mfp
10 mfp
15 mfp
20 mfp
25 mfp
30 mfp
35 mfp
40 mfp
Exposure buildup factor
Photon energy (MeV)
0.013
0.1
1
10
17

(c)

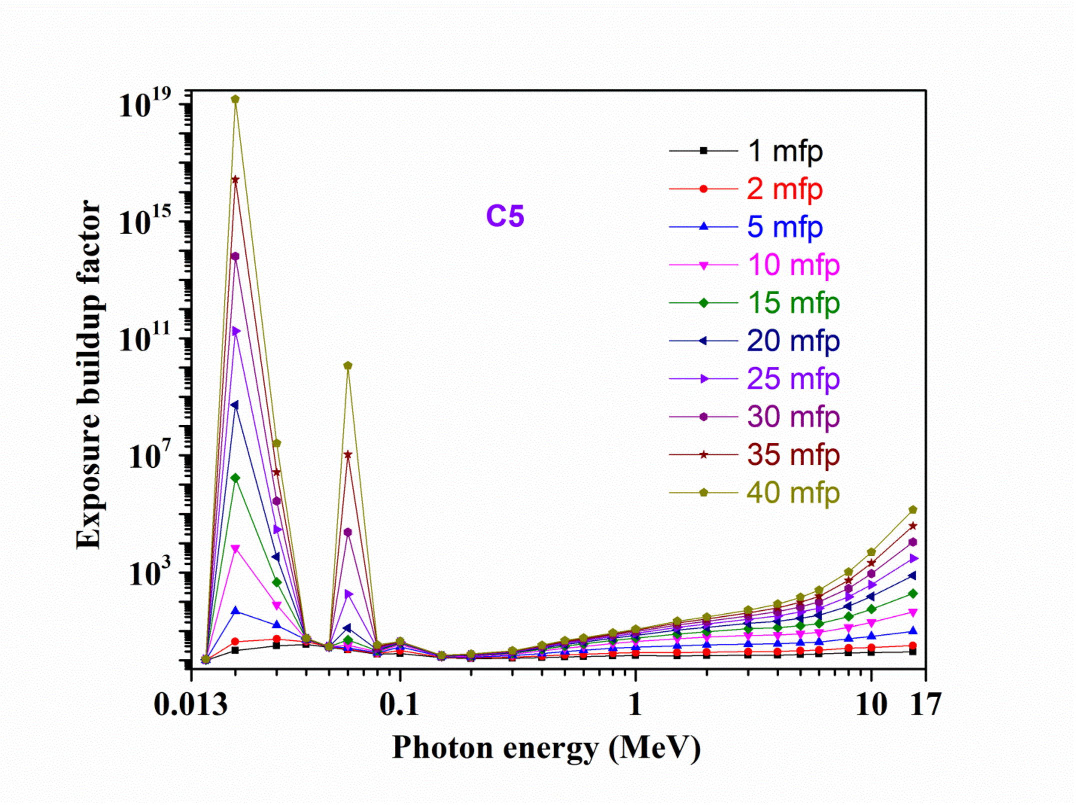
C5
1 mfp
2 mfp
5 mfp
10 mfp
15 mfp
20 mfp
25 mfp
30 mfp
35 mfp
40 mfp
Exposure buildup factor
Photon energy (MeV)
0.013
0.1
1
10
17

(d)

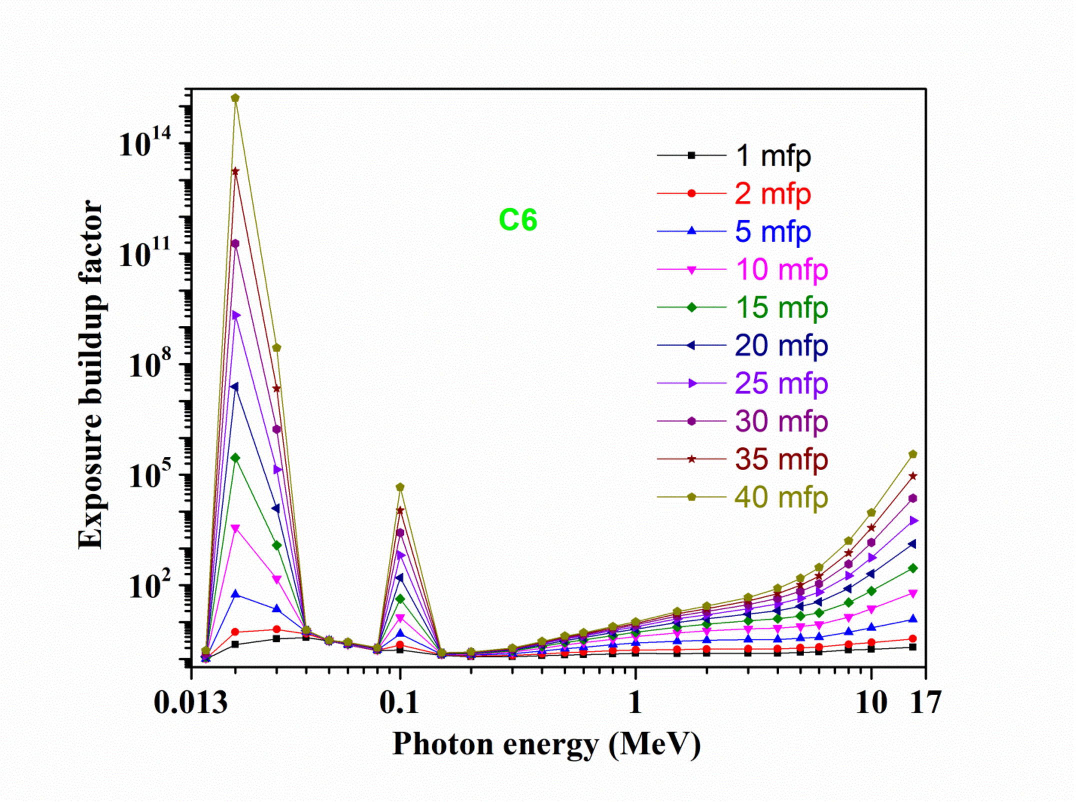

Exposure buildup factor
10^{14}
10^{11}
10^{8}
10^{5}
10^{2}
C6
1 mfp
2 mfp
5 mfp
10 mfp
15 mfp
20 mfp
25 mfp
30 mfp
35 mfp
40 mfp
0.013
0.1
1
10
17
Photon energy (MeV)

(e)

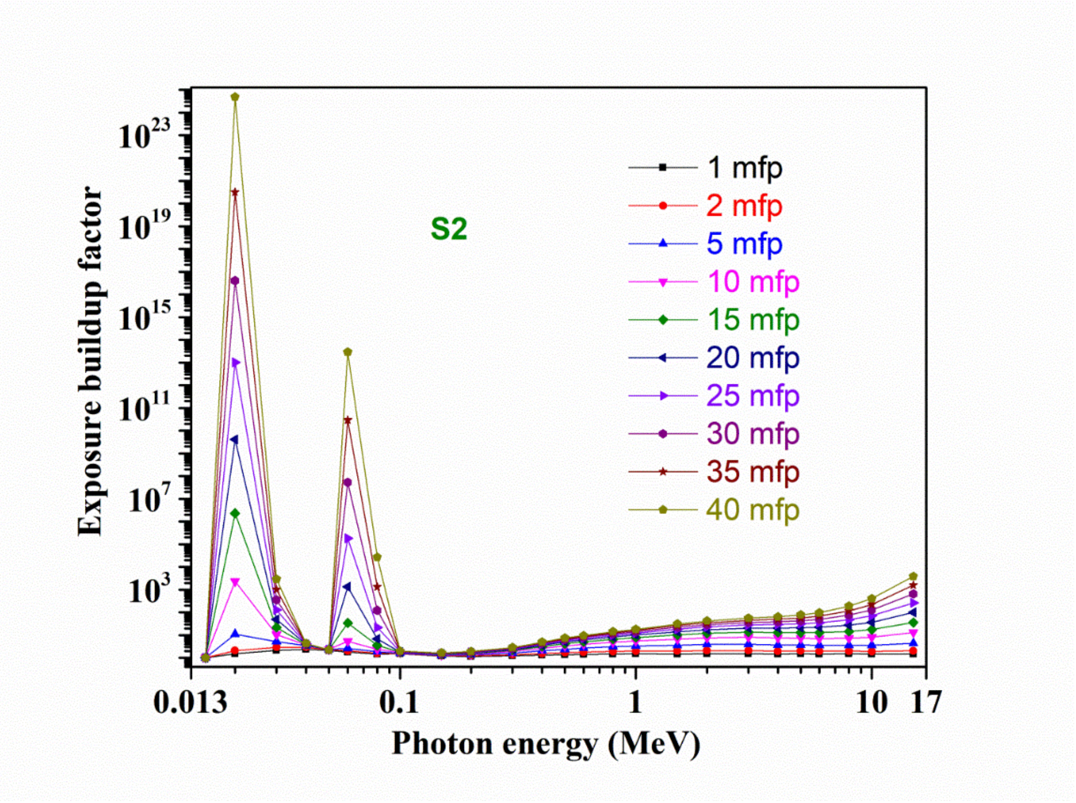

Exposure buildup factor
10^{23}
10^{19}
10^{15}
10^{11}
10^{7}
10^{3}
S2
1 mfp
2 mfp
5 mfp
10 mfp
15 mfp
20 mfp
25 mfp
30 mfp
35 mfp
40 mfp
0.013
0.1
1
10
17
Photon energy (MeV)

(f)

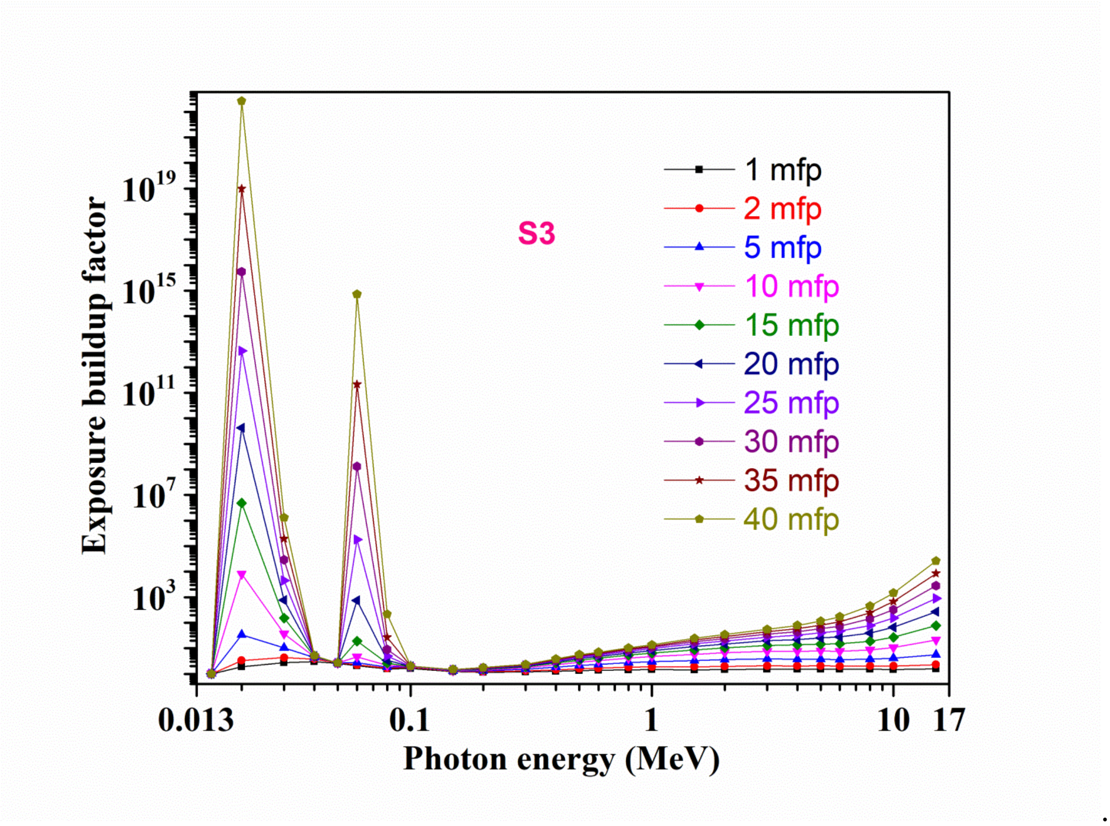
S3
1 mfp
2 mfp
5 mfp
10 mfp
15 mfp
20 mfp
25 mfp
30 mfp
35 mfp
40 mfp
Exposure buildup factor
10^{19}
10^{15}
10^{11}
10^{7}
10^{3}
0.013
0.1
1
10
17
Photon energy (MeV)

(g)

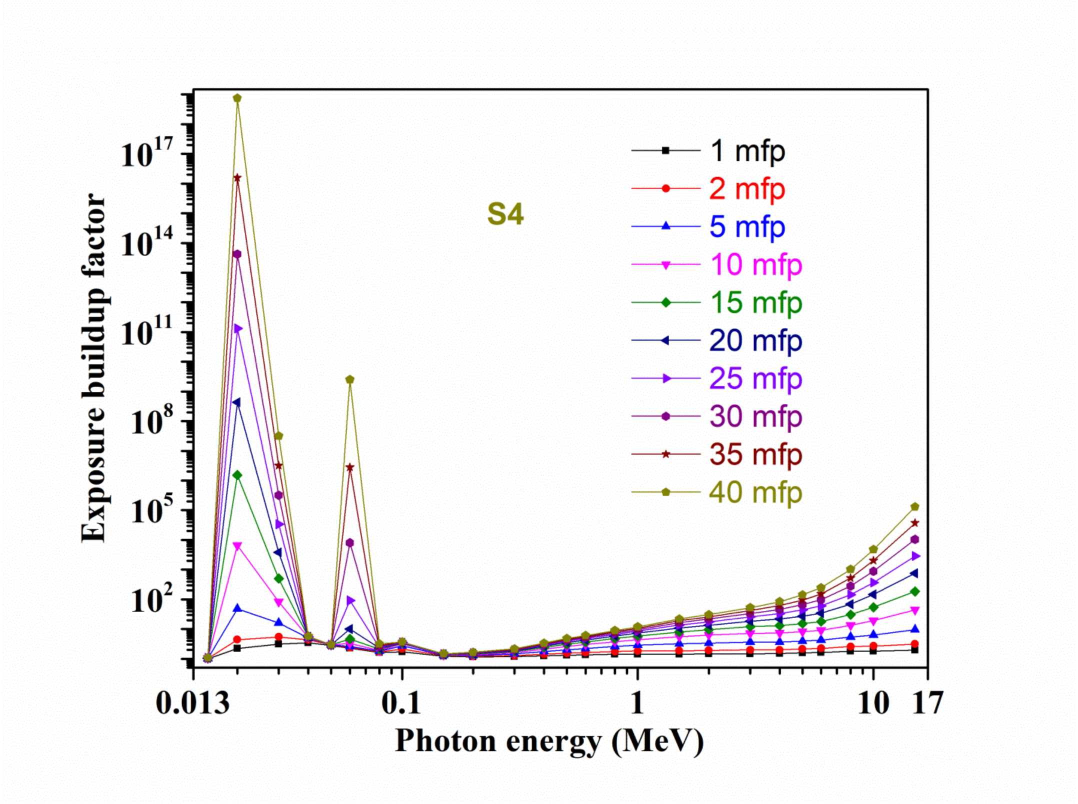
S4
1 mfp
2 mfp
5 mfp
10 mfp
15 mfp
20 mfp
25 mfp
30 mfp
35 mfp
40 mfp
Exposure buildup factor
10^{17}
10^{14}
10^{11}
10^{8}
10^{5}
10^{2}
0.013
0.1
1
10
17
Photon energy (MeV)

(h)

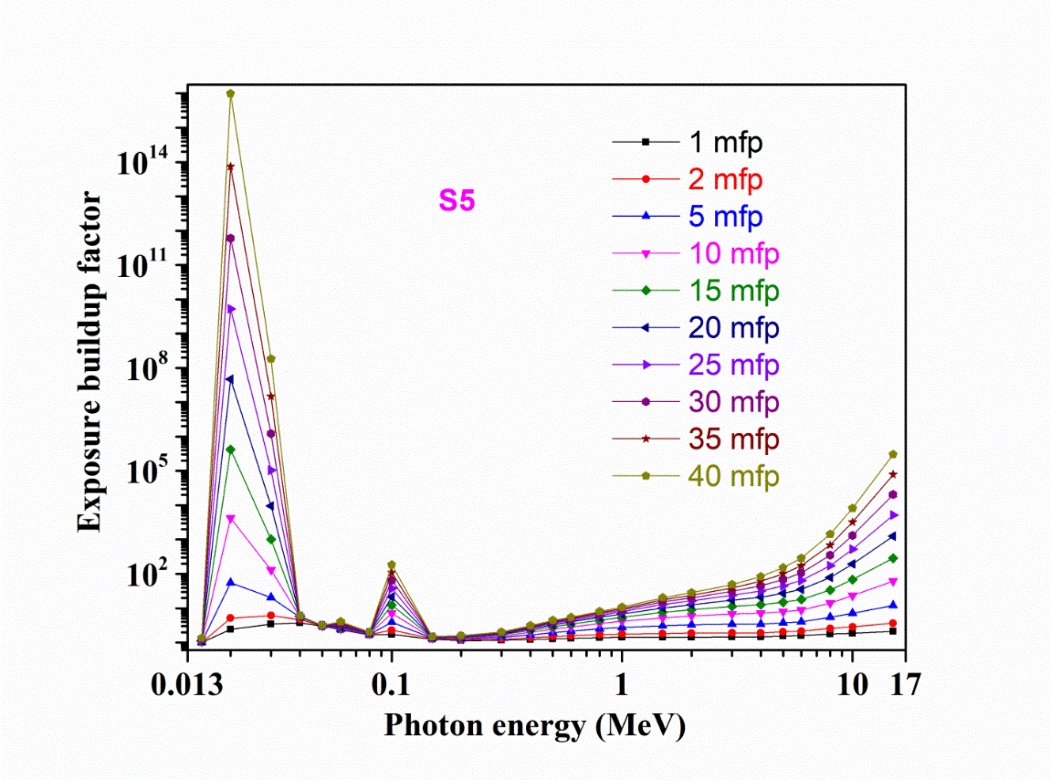

(i)

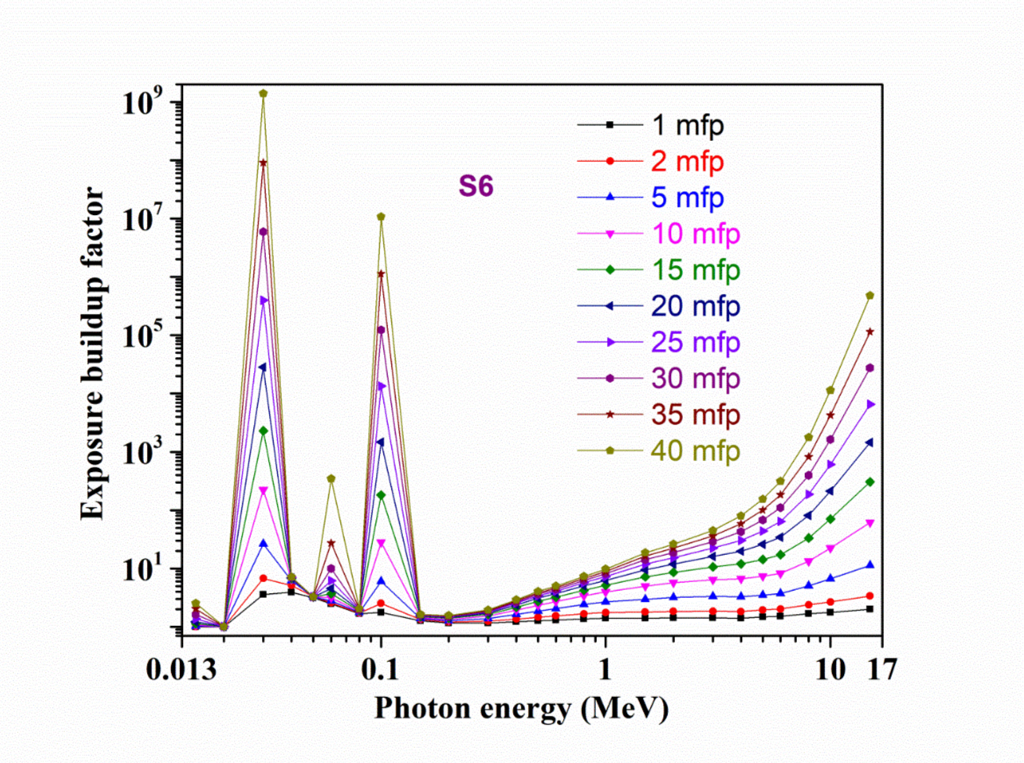

(j)

Figure S9. Variations of exposure buildup factor (EBF) with photon energy at different mean free paths for all (a-e) C2–C6 and (f-j) S2–S6 glasses.

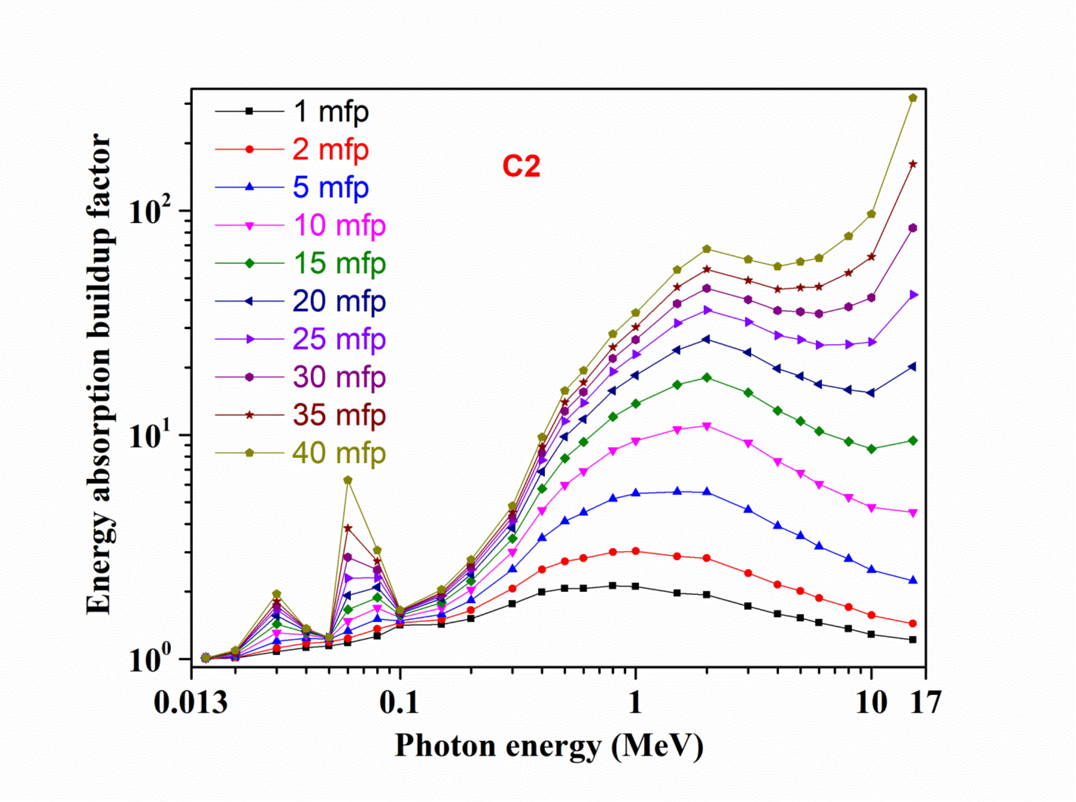
1 mfp
2 mfp
5 mfp
10 mfp
15 mfp
20 mfp
25 mfp
30 mfp
35 mfp
40 mfp
C2
Energy absorption buildup factor
Photon energy (MeV)
0.013
0.1
1
10
17

(a)

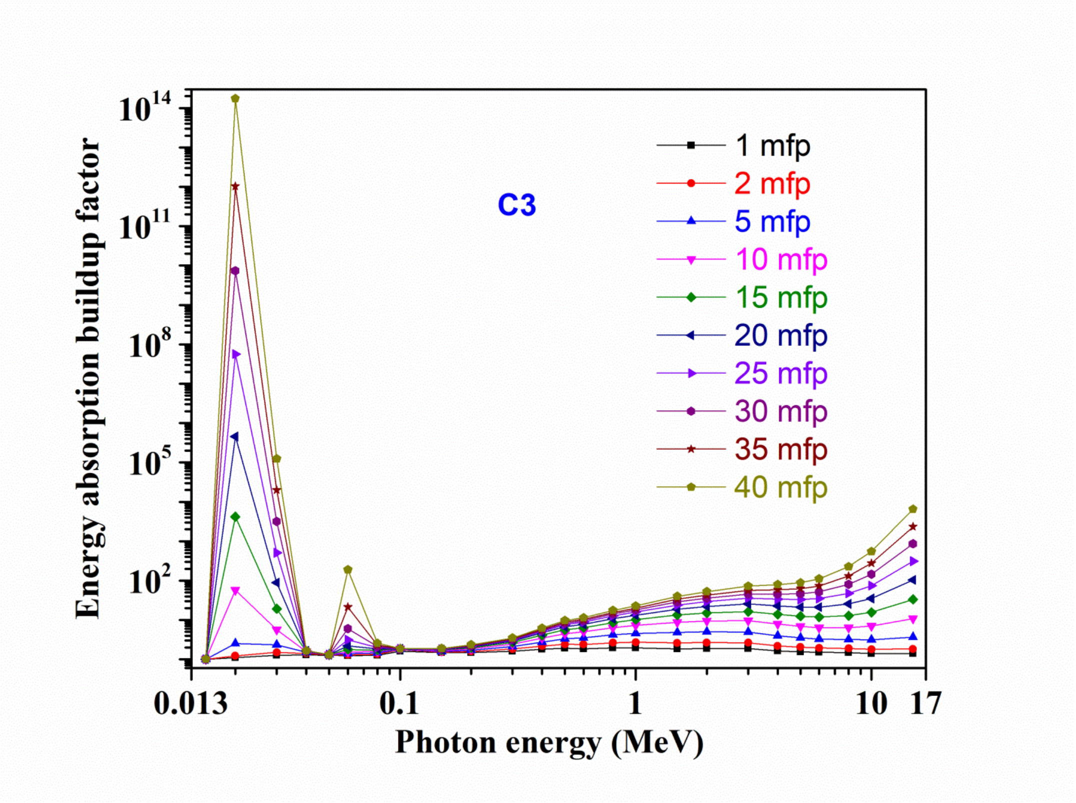
1 mfp
2 mfp
5 mfp
10 mfp
15 mfp
20 mfp
25 mfp
30 mfp
35 mfp
40 mfp
C3
Energy absorption buildup factor
Photon energy (MeV)
0.013
0.1
1
10
17

(b)

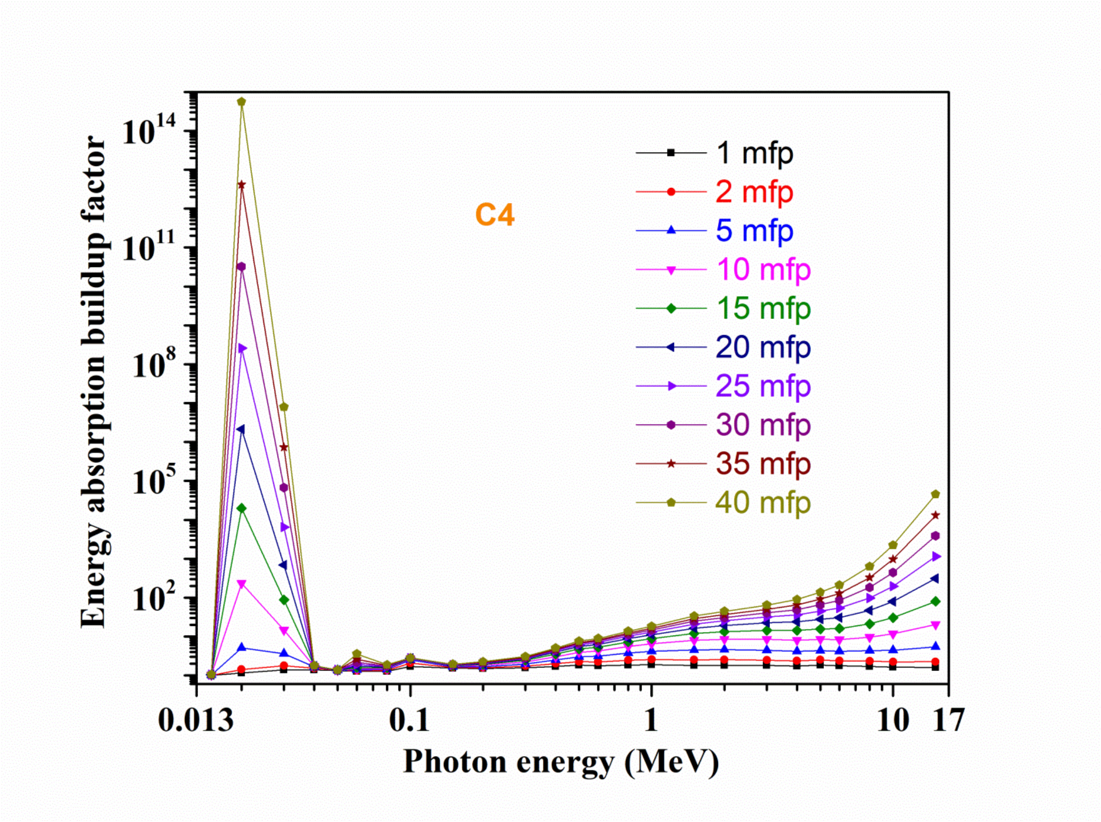

Energy absorption buildup factor
10^{14}
10^{11}
10^{8}
10^{5}
10^{2}
0.013
0.1
1
10
17
Photon energy (MeV)
C4
1 mfp
2 mfp
5 mfp
10 mfp
15 mfp
20 mfp
25 mfp
30 mfp
35 mfp
40 mfp

(c)

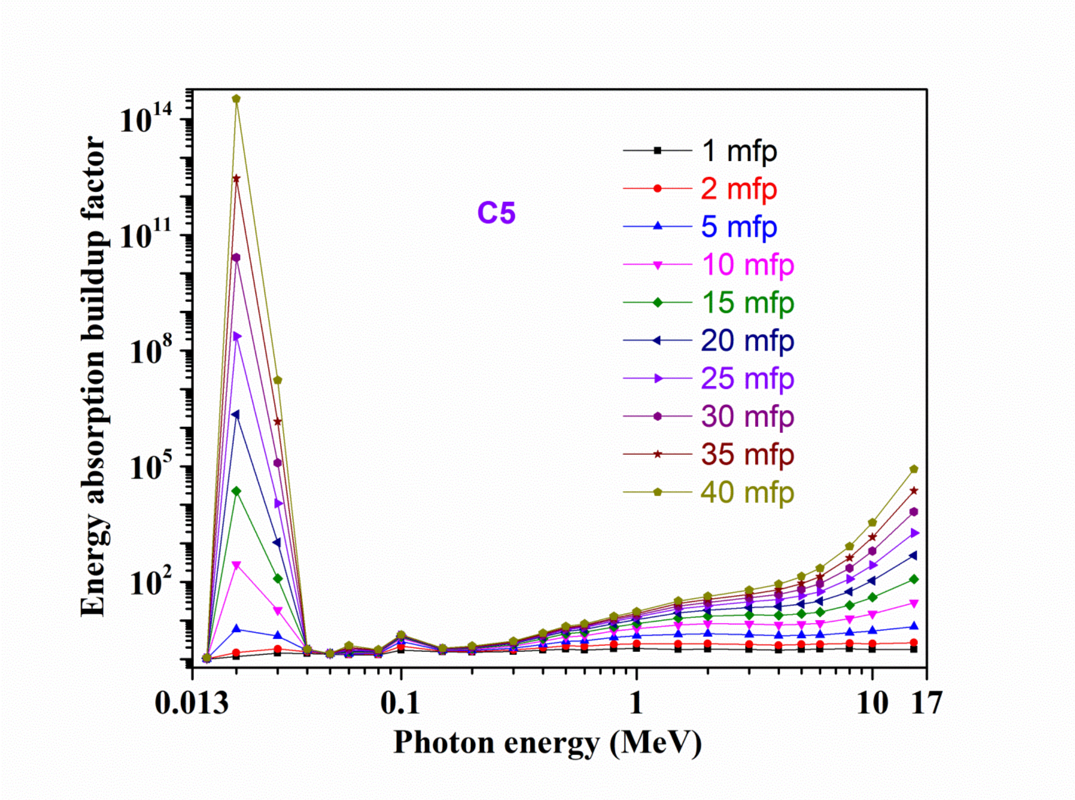

Energy absorption buildup factor
10^{14}
10^{11}
10^{8}
10^{5}
10^{2}
0.013
0.1
1
10
17
Photon energy (MeV)
C5
1 mfp
2 mfp
5 mfp
10 mfp
15 mfp
20 mfp
25 mfp
30 mfp
35 mfp
40 mfp

(d)

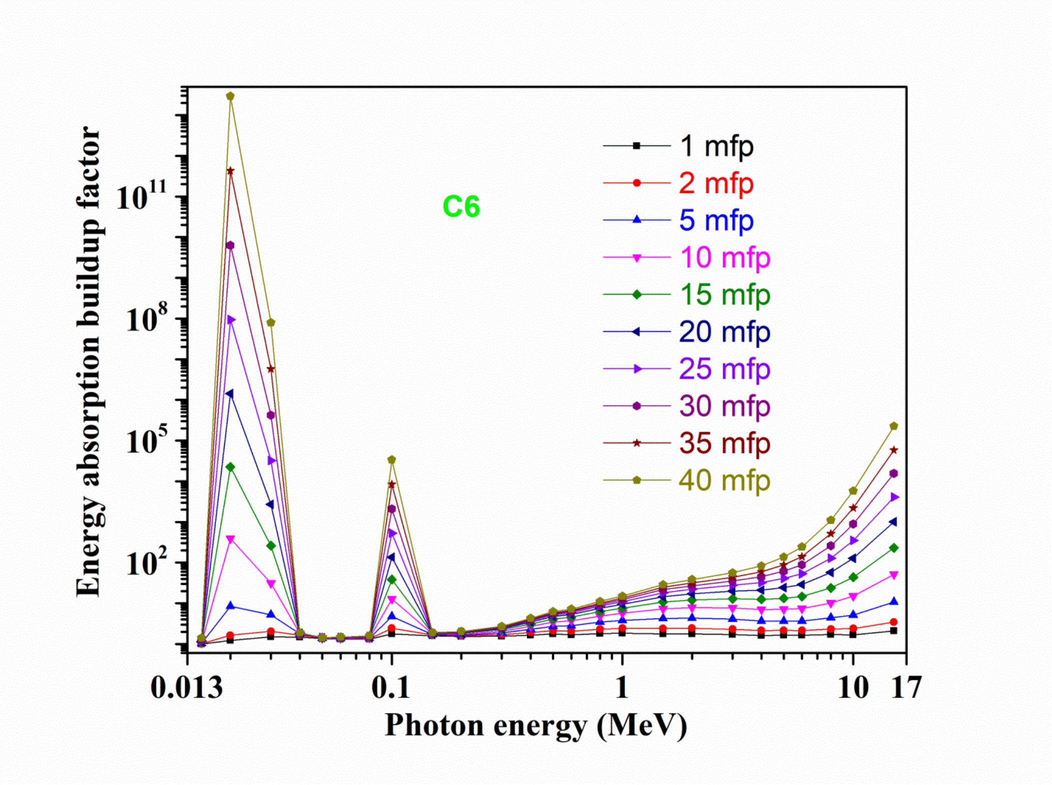
C6
1 mfp
2 mfp
5 mfp
10 mfp
15 mfp
20 mfp
25 mfp
30 mfp
35 mfp
40 mfp
Energy absorption buildup factor
10^{11}
10^8
10^5
10^2
0.013
0.1
1
10
17
Photon energy (MeV)

(e)

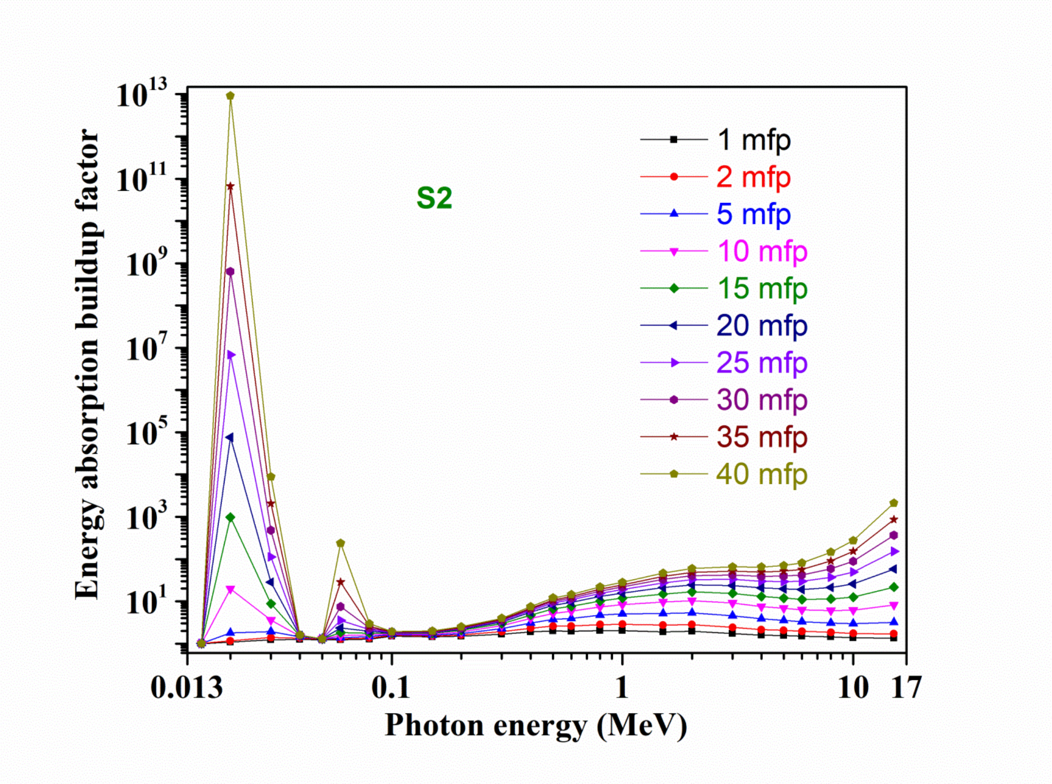
S2
1 mfp
2 mfp
5 mfp
10 mfp
15 mfp
20 mfp
25 mfp
30 mfp
35 mfp
40 mfp
Energy absorption buildup factor
10^{13}
10^{11}
10^9
10^7
10^5
10^3
10^1
0.013
0.1
1
10
17
Photon energy (MeV)

(f)

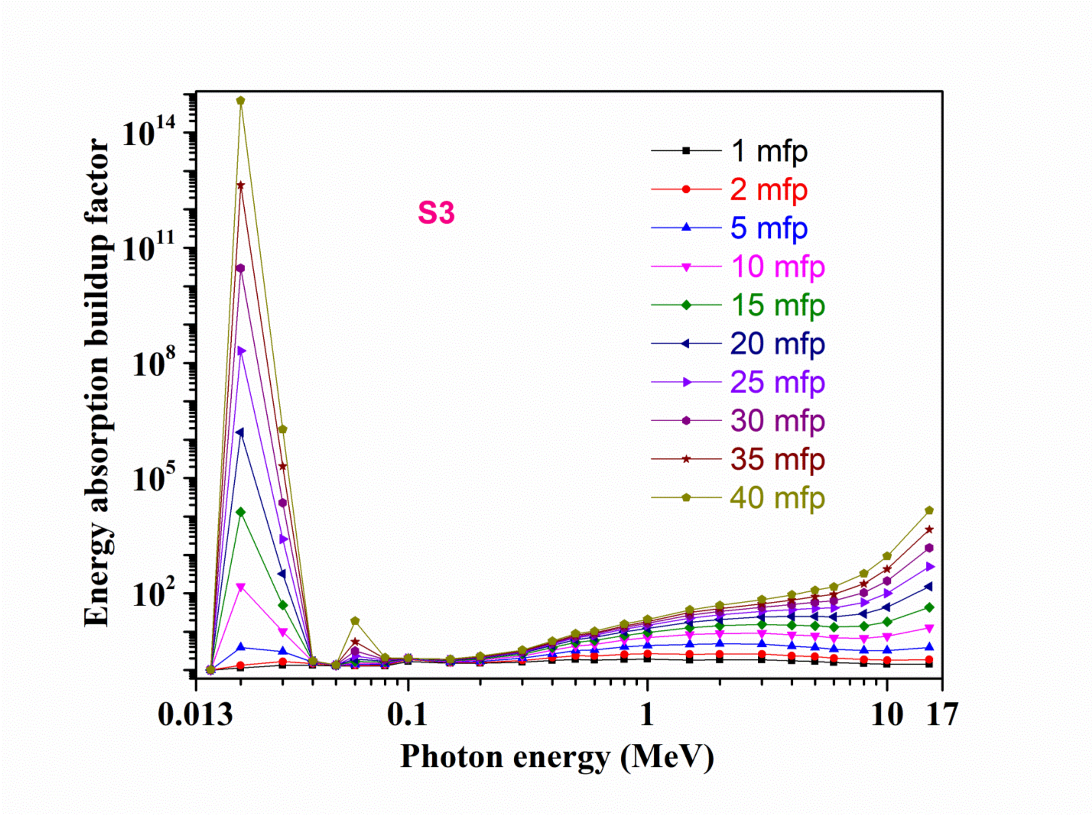
Energy absorption buildup factor
10^{14}
10^{11}
10^{8}
10^{5}
10^{2}
S3
1 mfp
2 mfp
5 mfp
10 mfp
15 mfp
20 mfp
25 mfp
30 mfp
35 mfp
40 mfp
0.013
0.1
1
10
17
Photon energy (MeV)

(g)

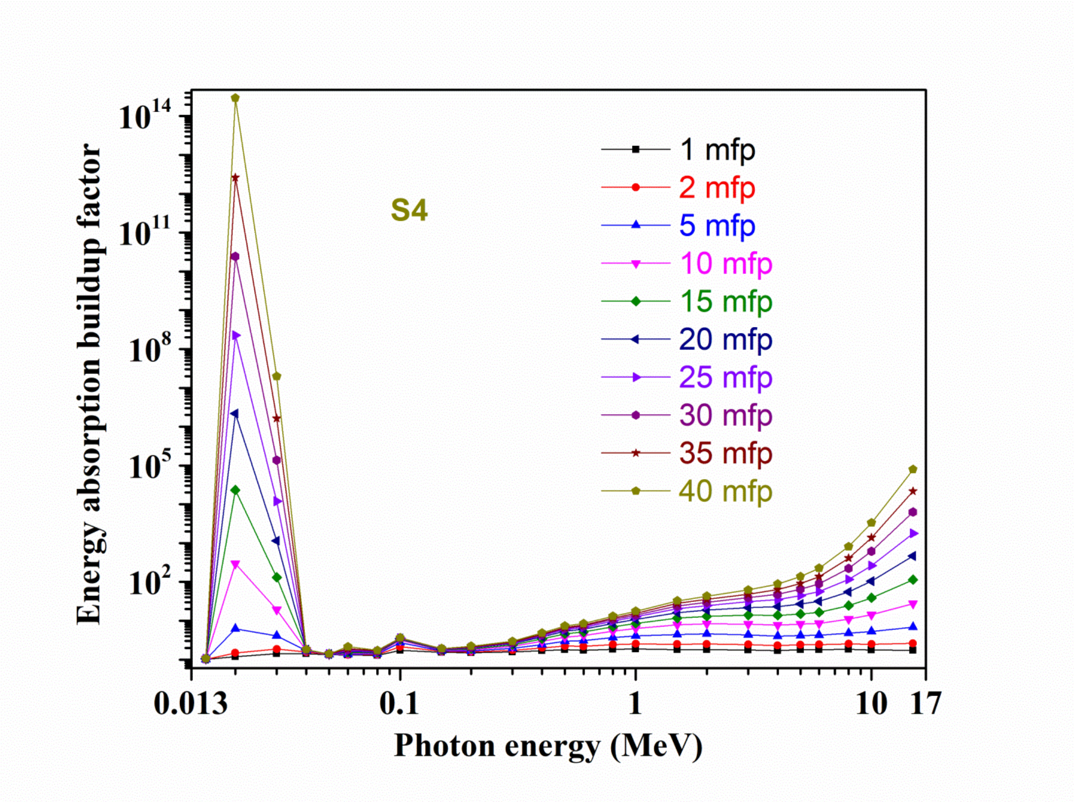
Energy absorption buildup factor
10^{14}
10^{11}
10^{8}
10^{5}
10^{2}
S4
1 mfp
2 mfp
5 mfp
10 mfp
15 mfp
20 mfp
25 mfp
30 mfp
35 mfp
40 mfp
0.013
0.1
1
10
17
Photon energy (MeV)

(h)

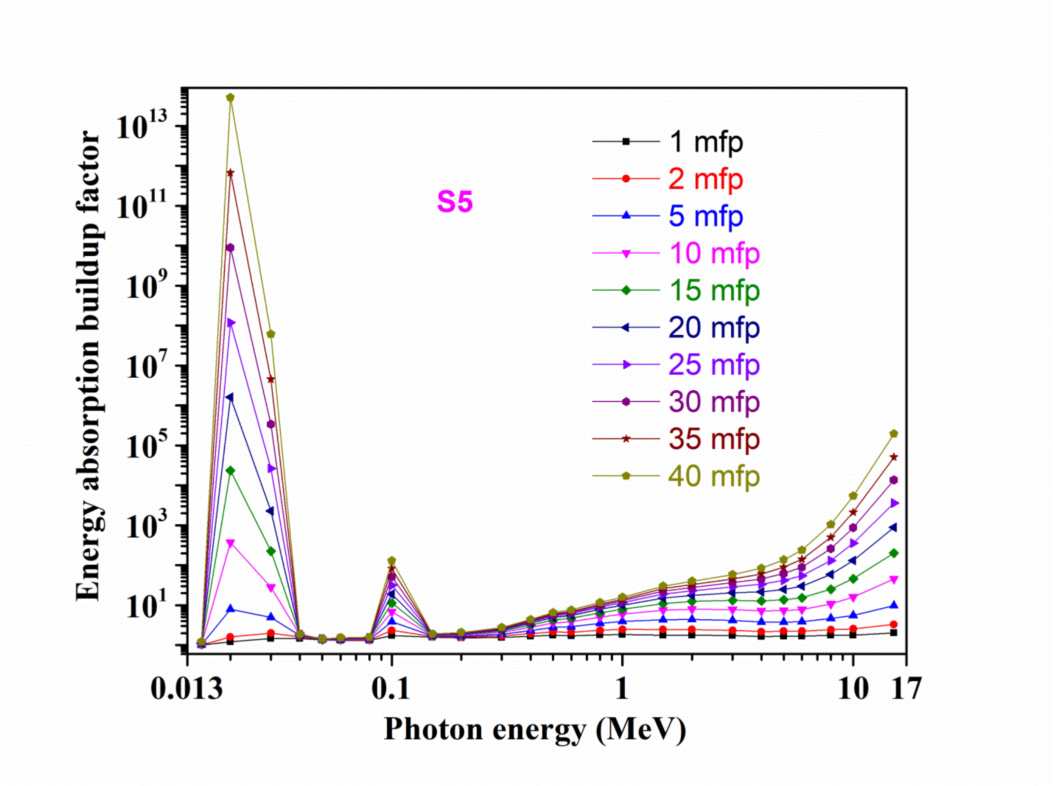

(i)

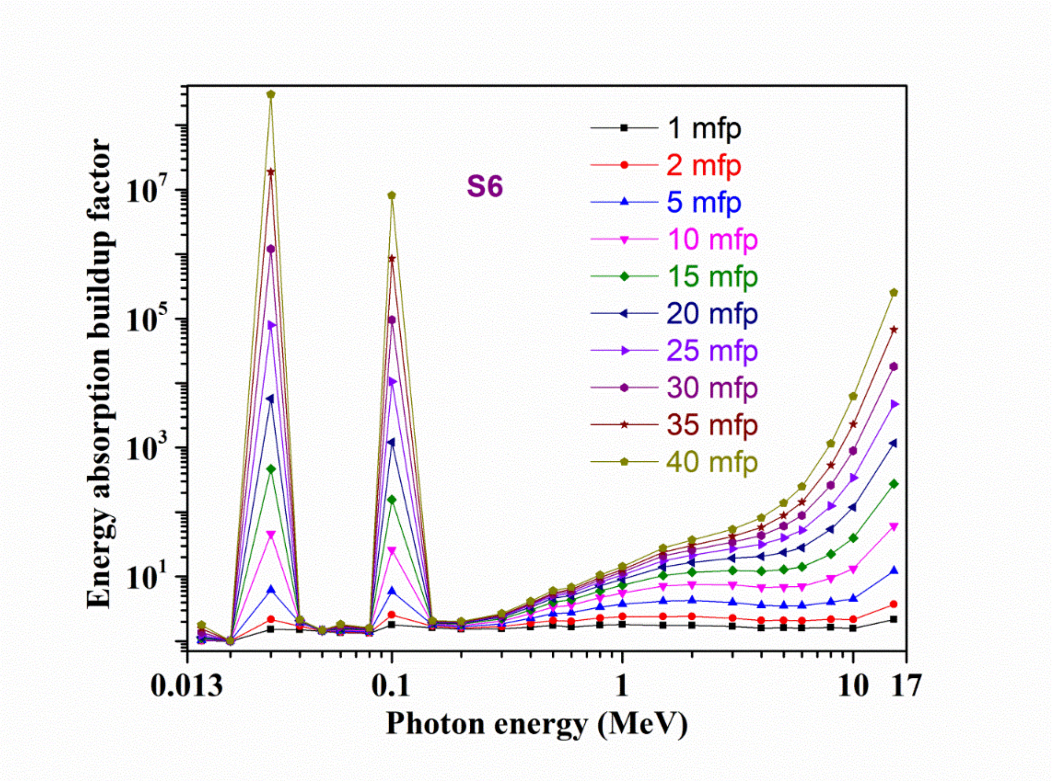

(j)

Figure S10. Variations of energy absorption buildup factor (EABF) with photon energy at different mean free paths for all (a-e) C2–C6 and (f-j) S2–S6 glasses.

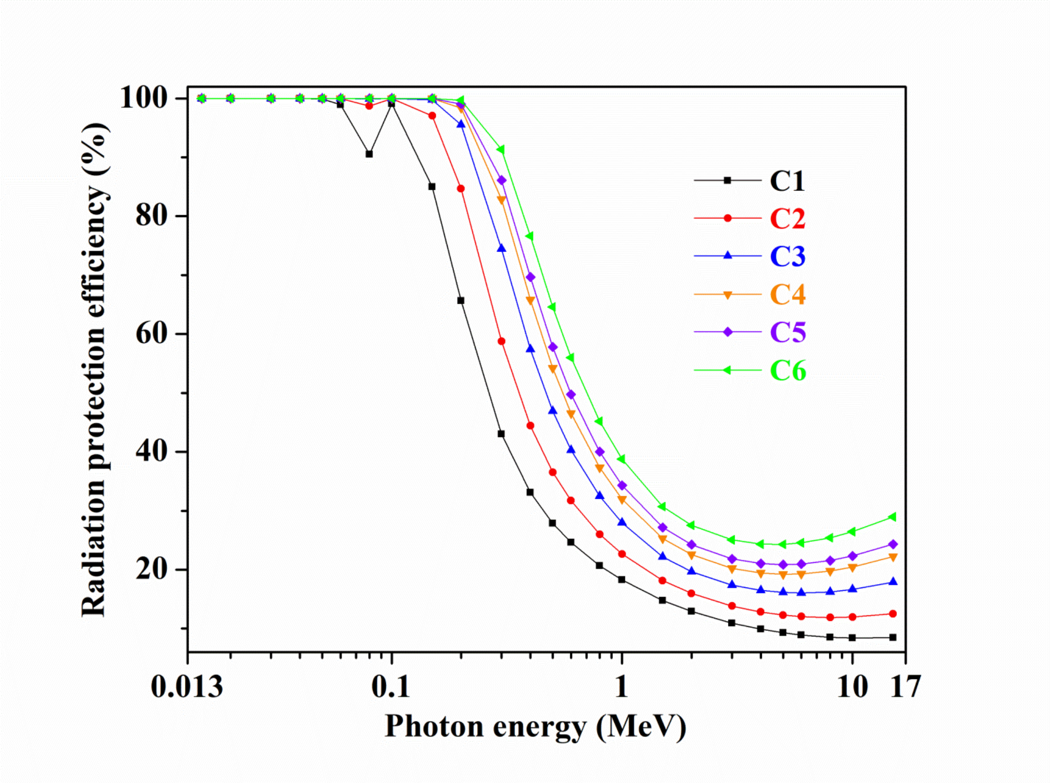

Figure S11. Variations of radiation protection efficiency (RPE) with photon energy (MeV) for all C1–C6 glasses.

Table S1. Mass attenuation coefficients (μ/ϱ) of all C1–C6 glasses estimated using (i) Phy-X/PSD program (ii) MCNPX (iii) Geant4 and (iv) Penelope codes.

(i) Phy-X/PSD program

Energy (MeV)	Sample code					
	C1	C2	C3	C4	C5	C6
0.015	32.569	50.21	68.51	80.572	83.502	91.727
0.02	23.715	37.412	52.177	61.534	64.048	70.562
0.03	8.341	13.152	18.372	21.658	22.556	24.851
0.04	3.996	6.2667	8.7402	10.291	10.719	11.805
0.05	2.282	3.5439	4.9224	5.7844	6.0238	6.6281
0.06	1.464	2.2436	3.0968	3.6292	3.7779	4.1516
0.08	0.759	1.1232	1.5229	1.7717	1.8416	2.0164
0.1	1.520	2.3829	3.3515	3.9406	4.1155	4.5347
0.15	0.610	0.9115	1.2497	1.4553	1.5164	1.6628
0.2	0.344	0.4855	0.6437	0.74	0.7685	0.837
0.3	0.181	0.2294	0.2834	0.3164	0.3261	0.3495
0.4	0.129	0.152	0.1772	0.1926	0.1971	0.208
0.5	0.105	0.1177	0.1315	0.14	0.1425	0.1485
0.6	0.091	0.0987	0.1071	0.1122	0.1137	0.1174
0.8	0.075	0.078	0.0816	0.0838	0.0844	0.086
1	0.065	0.0665	0.0681	0.0692	0.0694	0.0702
1.5	0.051	0.0518	0.0521	0.0523	0.0524	0.0525
2	0.045	0.045	0.0455	0.0458	0.0458	0.0461
3	0.037	0.0385	0.0397	0.0405	0.0407	0.0412
4	0.034	0.0355	0.0374	0.0387	0.039	0.0399
5	0.031	0.034	0.0365	0.0383	0.0386	0.0398
6	0.030	0.0332	0.0363	0.0384	0.0389	0.0403
8	0.029	0.0327	0.0368	0.0395	0.0401	0.0419
10	0.028	0.033	0.0378	0.041	0.0418	0.0439
15	0.029	0.0346	0.0409	0.0451	0.0461	0.0489

(ii) MCNPX code

Energy (MeV)	Sample code					
	C1	C2	C3	C4	C5	C6
0.015	32.857143	50.605812	68.54781	80.704324	83.602142	91.803251
0.02	24.086651	37.586614	52.32745	61.638751	64.125644	70.632744
0.03	8.452330	13.195230	18.46521	21.746336	22.598742	24.963562
0.04	4.087443	6.295471	8.866254	10.308734	10.756983	11.856915
0.05	2.300875	3.600854	4.99851	5.798541	6.109854	6.630984
0.06	1.531446	2.306217	3.106981	3.636587	3.799875	4.163254
0.08	0.768741	1.141246	1.545417	1.785476	1.863571	2.045571
0.1	1.535975	2.390651	3.378355	3.959874	4.132964	4.545876
0.15	0.619554	0.920574	1.250972	1.463587	1.521743	1.679324
0.2	0.351400	0.490651	0.650365	0.752174	0.775586	0.841782
0.3	0.188874	0.231174	0.290744	0.318967	0.329873	0.350695
0.4	0.130985	0.156387	0.180654	0.195324	0.199751	0.209351
0.5	0.110775	0.119541	0.140632	0.146373	0.143623	0.149532
0.6	0.100054	0.099871	0.112718	0.113288	0.115214	0.118047
0.8	0.075954	0.079844	0.082324	0.083984	0.085693	0.087462
1	0.066338	0.067324	0.069065	0.070543	0.070416	0.071087
1.5	0.052743	0.052174	0.053271	0.054539	0.053975	0.053065
2	0.046089	0.046338	0.045987	0.048754	0.046398	0.046874
3	0.038047	0.039674	0.039951	0.041247	0.040899	0.041365
4	0.034681	0.036991	0.037514	0.039365	0.039327	0.040542
5	0.032005	0.034875	0.036637	0.038693	0.038706	0.039951
6	0.032317	0.033636	0.036478	0.038658	0.039005	0.040605
8	0.029782	0.0328969	0.036994	0.039861	0.040354	0.042004

10	0.028887	0.033565	0.037866	0.041563	0.041954	0.044052
15	0.029691	0.034861	0.041402	0.045284	0.046239	0.049056

(iii) Geant4 code

Energy (MeV)	Sample code					
	C1	C2	C3	C4	C5	C6
0.015	32.5480	50.1753	68.4595	80.5110	83.4382	91.6563
0.02	23.6252	37.2658	51.9656	61.2843	63.7864	70.2718
0.03	8.3366	13.1451	18.3617	21.6463	22.5433	24.8374
0.04	3.9881	6.2536	8.7212	10.2686	10.6954	11.7785
0.05	2.2728	3.5291	4.9010	5.7590	5.9973	6.5988
0.06	1.4542	2.2277	3.0740	3.6022	3.7496	4.1203
0.08	0.7475	1.1046	1.4961	1.7400	1.8084	1.9797
0.1	1.5140	2.3733	3.3377	3.9242	4.0984	4.5158
0.15	0.6081	0.9081	1.2448	1.4495	1.5104	1.6561
0.2	0.3435	0.4840	0.6416	0.7375	0.7660	0.8342
0.3	0.1806	0.2285	0.2821	0.3148	0.3245	0.3477
0.4	0.1291	0.1514	0.1763	0.1916	0.1960	0.2068
0.5	0.1050	0.1172	0.1309	0.1393	0.1417	0.1476
0.6	0.0908	0.0983	0.1065	0.1116	0.1130	0.1166
0.8	0.0745	0.0777	0.0812	0.0834	0.0840	0.0855
1	0.0648	0.0663	0.0678	0.0688	0.0691	0.0698
1.5	0.0513	0.0516	0.0519	0.0521	0.0521	0.0523
2	0.0444	0.0449	0.0453	0.0457	0.0457	0.0459
3	0.0372	0.0384	0.0396	0.0404	0.0406	0.0411
4	0.0335	0.0354	0.0373	0.0387	0.0389	0.0398
5	0.0314	0.0339	0.0365	0.0382	0.0386	0.0397
6	0.0301	0.0331	0.0362	0.0383	0.0388	0.0402
8	0.0287	0.0327	0.0367	0.0394	0.0401	0.0419
10	0.0283	0.0330	0.0377	0.0410	0.0417	0.0439
15	0.0285	0.0346	0.0409	0.0451	0.0460	0.0489

(iv) Penelope code

Energy (MeV)	Sample code					
	C1	C2	C3	C4	C5	C6
0.015	31.9019	49.1454	67.0038	78.7932	81.6587	89.6775
0.02	23.1587	36.5134	50.8941	60.0176	62.4735	68.8096
0.03	8.1860	12.9012	18.0138	21.2346	22.1168	24.3620
0.04	3.9209	6.1444	8.5652	10.0838	10.5041	11.5650
0.05	2.2370	3.4706	4.8173	5.6598	5.8946	6.4841
0.06	1.4332	2.1930	3.0242	3.5432	3.6885	4.0520
0.08	0.7382	1.0892	1.4739	1.7136	1.7810	1.9491
0.1	1.4952	2.3422	3.2929	3.8711	4.0435	4.4542
0.15	0.6031	0.8996	1.2325	1.4349	1.4953	1.6390
0.2	0.3409	0.4796	0.6353	0.7300	0.7582	0.8255
0.3	0.1798	0.2271	0.2801	0.3124	0.3219	0.3448
0.4	0.1287	0.1507	0.1753	0.1903	0.1948	0.2054
0.5	0.1047	0.1169	0.1303	0.1386	0.1410	0.1468
0.6	0.0907	0.0981	0.1062	0.1112	0.1126	0.1162
0.8	0.0744	0.0776	0.0810	0.0832	0.0838	0.0853
1	0.0647	0.0662	0.0677	0.0687	0.0690	0.0697
1.5	0.0513	0.0516	0.0519	0.0521	0.0521	0.0522
2	0.0444	0.0449	0.0453	0.0456	0.0456	0.0458
3	0.0372	0.0384	0.0396	0.0404	0.0406	0.0411
4	0.0335	0.0354	0.0373	0.0386	0.0389	0.0398
5	0.0314	0.0339	0.0364	0.0382	0.0385	0.0397
6	0.0301	0.0331	0.0362	0.0383	0.0388	0.0402

8	0.0287	0.0327	0.0367	0.0394	0.0400	0.0419
10	0.0282	0.0329	0.0377	0.0409	0.0417	0.0439
15	0.0285	0.0346	0.0409	0.0450	0.0460	0.0489

Table S2. Mass attenuation coefficients (μ/ϱ) of all S1–S6 glasses estimated using (i) Phy-X/PSD program (ii) MCNPX (iii) Geant4 and (iv) Penelope codes

(i) Phy-X/PSD program

Energy (MeV)	Sample code					
	S1	S2	S3	S4	S5	S6
0.015	30.582	56.867	71.979	81.391	87.835	94.419
0.02	35.998	48.395	57.847	64.685	69.367	74.151
0.03	12.473	16.983	20.347	22.757	24.408	26.094
0.04	5.851	8.0517	9.6596	10.801	11.583	12.381
0.05	3.263	4.522	5.4259	6.0624	6.4981	6.9434
0.06	2.041	2.8393	3.4033	3.7976	4.0675	4.3434
0.08	1.005	1.3941	1.6621	1.8473	1.9741	2.1036
0.1	1.503	2.8285	3.5777	4.0392	4.3551	4.6779
0.15	0.596	1.0639	1.327	1.4884	1.5989	1.7118
0.2	0.334	0.5555	0.6792	0.7549	0.8067	0.8596
0.3	0.175	0.2524	0.2951	0.321	0.3388	0.357
0.4	0.125	0.1621	0.1823	0.1945	0.2028	0.2113
0.5	0.102	0.1228	0.1341	0.1409	0.1455	0.1502
0.6	0.088	0.1015	0.1085	0.1126	0.1154	0.1183
0.8	0.073	0.0787	0.0819	0.0838	0.085	0.0863
1	0.063	0.0665	0.0681	0.069	0.0696	0.0702
1.5	0.050	0.0513	0.0519	0.0521	0.0523	0.0524
2	0.044	0.0448	0.0453	0.0456	0.0458	0.046
3	0.037	0.0388	0.0398	0.0405	0.0409	0.0414
4	0.034	0.0363	0.0378	0.0388	0.0395	0.0402
5	0.032	0.0351	0.0371	0.0385	0.0394	0.0403
6	0.031	0.0347	0.0371	0.0387	0.0398	0.0409
8	0.030	0.0348	0.0379	0.0399	0.0413	0.0427
10	0.030	0.0355	0.0391	0.0415	0.0432	0.0449
15	0.031	0.038	0.0427	0.0458	0.048	0.0502

(ii) MCNPX code

Energy (MeV)	Sample code					
	S1	S2	S3	S4	S5	S6
0.015	30.635742	56.932541	71.983365	81.407411	87.869851	94.456241
0.02	36.109814	48.412387	57.863651	64.696524	69.374451	74.178954
0.03	12.512965	16.995813	20.357434	22.763998	24.450688	26.105648
0.04	5.885377	8.100874	9.663074	10.806384	11.593372	12.390654
0.05	3.295267	4.563906	5.432147	6.063654	6.500413	6.948914
0.06	2.063086	2.845714	3.406221	3.798142	4.098541	4.344156
0.08	1.050631	1.403365	1.663221	1.848215	1.976384	2.104069
0.1	1.524185	2.832874	3.578065	4.039974	4.359852	4.679074
0.15	0.599874	1.086055	1.328064	1.489008	1.600540	1.713261
0.2	0.335039	0.560380	0.679974	0.755047	0.810746	0.860094
0.3	0.176054	0.256371	0.296327	0.321563	0.339997	0.358412
0.4	0.126310	0.164571	0.183058	0.194893	0.203621	0.212361
0.5	0.104682	0.123652	0.134638	0.141004	0.146174	0.151274
0.6	0.089151	0.103650	0.108983	0.112865	0.116032	0.119063
0.8	0.073257	0.079541	0.082047	0.083912	0.085852	0.087031
1	0.064165	0.067411	0.068633	0.069537	0.069984	0.071421
1.5	0.051216	0.052321	0.052074	0.052325	0.052463	0.052563
2	0.043965	0.045699	0.045638	0.045714	0.045991	0.046515
3	0.037031	0.039065	0.039997	0.040612	0.041054	0.041532
4	0.034055	0.036458	0.037955	0.038925	0.039864	0.040315
5	0.032099	0.035450	0.037326	0.038632	0.039526	0.040412
6	0.031021	0.034932	0.037274	0.038800	0.039997	0.041065

8	0.031078	0.034991	0.037985	0.040054	0.041417	0.042803
10	0.030652	0.035745	0.039234	0.041601	0.043326	0.044997
15	0.031563	0.038174	0.042813	0.045854	0.048164	0.050325

(iii) Geant4 code

Energy (MeV)	Sample code					
	S1	S2	S3	S4	S5	S6
0.015	30.4967	56.8002	71.9114	81.3181	87.7584	94.3393
0.02	35.8883	48.2103	57.6165	64.4250	69.0864	73.8497
0.03	12.4730	16.9765	20.3371	22.7458	24.3950	26.0801
0.04	5.8462	8.0371	9.6399	10.7785	11.5581	12.3547
0.05	3.2559	4.5050	5.4034	6.0367	6.4703	6.9133
0.06	2.0341	2.8210	3.3792	3.7701	4.0378	4.3113
0.08	0.9953	1.3722	1.6337	1.8149	1.9390	2.0658
0.1	1.4985	2.8173	3.5631	4.0225	4.3370	4.6585
0.15	0.5938	1.0598	1.3218	1.4825	1.5925	1.7049
0.2	0.3335	0.5538	0.6770	0.7524	0.8040	0.8567
0.3	0.1748	0.2513	0.2937	0.3195	0.3371	0.3552
0.4	0.1250	0.1614	0.1814	0.1935	0.2017	0.2102
0.5	0.1017	0.1223	0.1334	0.1401	0.1447	0.1493
0.6	0.0881	0.1010	0.1079	0.1119	0.1147	0.1176
0.8	0.0723	0.0784	0.0815	0.0833	0.0845	0.0858
1	0.0629	0.0662	0.0678	0.0687	0.0692	0.0698
1.5	0.0499	0.0512	0.0517	0.0519	0.0520	0.0522
2	0.0434	0.0446	0.0452	0.0455	0.0457	0.0459
3	0.0368	0.0387	0.0397	0.0404	0.0408	0.0413
4	0.0336	0.0362	0.0377	0.0387	0.0394	0.0401
5	0.0319	0.0351	0.0371	0.0384	0.0393	0.0402
6	0.0309	0.0346	0.0370	0.0386	0.0397	0.0408
8	0.0301	0.0348	0.0378	0.0398	0.0412	0.0427
10	0.0300	0.0355	0.0391	0.0415	0.0431	0.0448
15	0.0310	0.0380	0.0426	0.0458	0.0479	0.0502

(iv) Penelope code

Energy (MeV)	Sample code					
	S1	S2	S3	S4	S5	S6
0.015	29.8409	55.5742	70.3547	79.5569	85.8573	92.2952
0.02	35.4244	47.3134	56.4707	63.1264	67.6833	72.3397
0.03	12.3277	16.6873	19.9659	22.3245	23.9394	25.5895
0.04	5.7802	7.9071	9.4731	10.5892	11.3534	12.1343
0.05	3.2206	4.4352	5.3138	5.9350	6.3603	6.7949
0.06	2.0126	2.7794	3.3259	3.7096	3.9722	4.2407
0.08	0.9861	1.3537	1.6099	1.7878	1.9097	2.0342
0.1	1.4814	2.7805	3.5153	3.9682	4.2782	4.5950
0.15	0.5891	1.0497	1.3086	1.4675	1.5762	1.6874
0.2	0.3311	0.5486	0.6703	0.7447	0.7956	0.8477
0.3	0.1741	0.2496	0.2915	0.3169	0.3344	0.3522
0.4	0.1246	0.1606	0.1803	0.1922	0.2004	0.2087
0.5	0.1015	0.1218	0.1328	0.1394	0.1439	0.1485
0.6	0.0880	0.1008	0.1075	0.1116	0.1143	0.1171
0.8	0.0723	0.0783	0.0814	0.0831	0.0843	0.0856
1	0.0629	0.0661	0.0677	0.0686	0.0691	0.0697
1.5	0.0499	0.0512	0.0516	0.0519	0.0520	0.0521

2	0.0434	0.0446	0.0451	0.0454	0.0456	0.0458
3	0.0368	0.0387	0.0397	0.0404	0.0408	0.0412
4	0.0336	0.0361	0.0377	0.0387	0.0394	0.0401
5	0.0318	0.0350	0.0370	0.0383	0.0392	0.0402
6	0.0309	0.0346	0.0370	0.0386	0.0397	0.0408
8	0.0301	0.0347	0.0378	0.0398	0.0412	0.0427
10	0.0300	0.0355	0.0391	0.0415	0.0431	0.0448
15	0.0310	0.0380	0.0426	0.0458	0.0479	0.0501

Table S3. Equivalent atomic numbers, G–P fitting parameters for EBF and EABF, respectively, for glass C1.

Energy (MeV)	Z_{eq}	G–P fitting parameters for EBF					G–P fitting parameters for EABF				
		**a**	**b**	**c**	**d**	**X_k**	**a**	**b**	**c**	**d**	**X_k**
0.015	20.49	-0.032	1.007	0.684	0.149	6.899	-0.020	1.007	0.674	0.133	9.343
0.02	24.33	0.509	1.013	0.209	-0.482	11.263	0.301	1.012	0.286	-0.265	16.457
0.03	25.42	0.193	1.030	0.374	-0.300	27.804	0.251	1.029	0.322	-0.187	18.356
0.04	26.18	0.247	1.057	0.336	-0.117	11.831	0.245	1.057	0.335	-0.142	14.673
0.05	26.77	0.235	1.091	0.363	-0.137	13.709	0.243	1.096	0.346	-0.137	13.994
0.06	27.25	0.221	1.131	0.387	-0.123	13.898	0.238	1.147	0.358	-0.142	14.627
0.08	28.03	0.188	1.213	0.454	-0.106	14.129	0.209	1.275	0.410	-0.118	14.615
0.1	45.81	0.488	1.276	0.179	-0.238	13.770	0.493	1.265	0.161	-0.263	13.601
0.15	47.87	0.253	1.225	0.367	-0.139	14.068	0.393	1.440	0.216	-0.216	13.920
0.2	49.09	0.171	1.283	0.501	-0.093	14.585	0.325	1.693	0.286	-0.192	13.932
0.3	50.57	0.108	1.397	0.641	-0.051	14.197	0.211	1.908	0.447	-0.120	13.796
0.4	51.43	0.070	1.506	0.772	-0.044	14.136	0.162	2.221	0.568	-0.113	13.868
0.5	52.01	0.048	1.573	0.851	-0.037	14.072	0.117	2.285	0.679	-0.089	13.873
0.6	52.38	0.030	1.606	0.910	-0.027	13.945	0.092	2.321	0.744	-0.076	13.723
0.8	52.81	0.014	1.643	0.972	-0.020	13.911	0.063	2.291	0.832	-0.059	13.622
1	52.96	0.007	1.649	1.005	-0.019	13.347	0.047	2.221	0.882	-0.051	13.513
1.5	50.32	-0.020	1.568	1.120	-0.005	14.268	0.006	1.921	1.030	-0.025	13.654
2	42.16	-0.021	1.600	1.127	-0.005	12.631	0.002	1.850	1.047	-0.025	13.071
3	30.92	-0.003	1.607	1.060	-0.017	12.319	0.007	1.681	1.026	-0.024	12.432
4	27.04	0.006	1.550	1.025	-0.021	12.926	0.013	1.557	1.003	-0.029	13.996
5	25.31	0.012	1.486	1.006	-0.026	13.129	0.024	1.478	0.968	-0.038	14.185
6	24.33	0.023	1.446	0.975	-0.034	13.325	0.025	1.403	0.969	-0.038	13.992
8	23.32	0.030	1.363	0.961	-0.040	13.596	0.034	1.312	0.947	-0.041	13.328
10	22.86	0.040	1.305	0.942	-0.049	13.788	0.042	1.254	0.931	-0.050	14.093
15	22.48	0.050	1.210	0.934	-0.057	14.115	0.040	1.159	0.956	-0.045	14.541

Table S4. Equivalent atomic numbers, G–P fitting parameters for EBF and EABF, respectively, for glass C2.

**Energy (MeV)**	Z_{eq}	G–P fitting parameters for EBF					G–P fitting parameters for EABF				
		**a**	**b**	**c**	**d**	X_k	**a**	**b**	**c**	**d**	X_k
0.015	24.5	-0.42	1.005	1.343	0.302	5.9228	-0.42	1.005	1.341	0.298	6.5302
0.02	29.24	0.747	1.044	0.146	-1.038	10.836	0.433	1.011	0.271	-0.499	12.733
0.03	30.34	0.193	1.339	0.448	-0.071	14.451	0.23	1.077	0.459	-0.17	13.299
0.04	31.11	0.21	1.591	0.328	-0.084	16.138	0.18	1.123	0.378	-0.242	25.028
0.05	31.69	0.109	1.56	0.277	-0.094	12.667	0.148	1.146	0.281	-0.078	11.789
0.06	32.17	0.48	1.473	0.263	-0.139	14.656	0.404	1.179	0.251	-0.162	14.637
0.08	32.93	0.39	1.351	0.304	-0.16	14.118	0.339	1.265	0.287	-0.159	14.27
0.1	54.7	0.669	1.447	0.044	-0.215	14.25	0.646	1.417	0.051	-0.227	14.007
0.15	56.7	0.358	1.195	0.24	-0.204	13.748	0.544	1.429	0.112	-0.286	13.754
0.2	57.86	0.199	1.189	0.442	-0.107	14.197	0.406	1.514	0.203	-0.232	13.83
0.3	59.22	0.135	1.283	0.568	-0.063	13.83	0.286	1.759	0.329	-0.163	13.487
0.4	60.02	0.092	1.374	0.693	-0.052	14.169	0.217	1.99	0.453	-0.143	13.824
0.5	60.53	0.068	1.439	0.772	-0.042	14.126	0.164	2.06	0.562	-0.113	13.864
0.6	60.87	0.051	1.482	0.828	-0.034	13.776	0.126	2.067	0.646	-0.09	13.682
0.8	61.25	0.032	1.537	0.895	-0.026	13.679	0.091	2.118	0.739	-0.072	13.604
1	61.4	0.02	1.554	0.945	-0.022	13.42	0.072	2.105	0.797	-0.063	13.517
1.5	59.6	-0	1.521	1.053	-0.013	13.782	0.041	1.967	0.911	-0.051	13.594
2	54.02	-0.01	1.554	1.07	-0.017	13.088	0.04	1.93	0.93	-0.055	13.238
3	43.67	9E-04	1.549	1.063	-0.029	12.874	0.037	1.718	0.949	-0.06	13.223
4	38.85	0.015	1.514	1.023	-0.039	13.267	0.045	1.588	0.929	-0.065	13.638
5	36.45	0.034	1.504	0.967	-0.055	13.464	0.059	1.524	0.894	-0.077	13.921
6	35.09	0.04	1.464	0.957	-0.059	13.534	0.061	1.449	0.895	-0.077	14.146
8	33.6	0.055	1.424	0.927	-0.072	13.758	0.066	1.365	0.893	-0.079	14.145
10	32.84	0.048	1.361	0.963	-0.065	14.011	0.056	1.289	0.931	-0.07	14.337
15	32.28	0.047	1.302	1.012	-0.063	14.387	0.059	1.218	0.965	-0.07	14.657

Table S5. Equivalent atomic numbers, G–P fitting parameters for EBF and EABF, respectively, for glass C3.

Energy (MeV)	Z_{eq}	G–P fitting parameters for EBF					G–P fitting parameters for EABF				
		a	b	c	d	X_k	a	b	c	d	X_k
0.015	28.41	-0.36	1.002	1.953	0.2709	10.099	-0.36	1.002	1.953	0.271	10.099
0.02	34.16	0.436	1.694	0.954	-0.597	11.895	0.255	1.104	1.033	-0.288	15.057
0.03	35.14	0.155	2.379	0.739	-0.137	22.438	0.174	1.275	0.748	-0.147	19.941
0.04	35.81	0.153	2.694	0.325	-0.06	19.532	0.146	1.295	0.351	-0.15	24.179
0.05	36.34	-0.1	2.313	0.161	-0.021	12.323	0.001	1.261	0.17	0.019	10.094
0.06	36.78	0.793	1.942	0.133	-0.143	16.093	0.602	1.253	0.137	-0.176	14.774
0.08	37.47	0.59	1.516	0.16	-0.211	14.268	0.463	1.283	0.178	-0.196	14.148
0.1	63.24	0.062	1.602	0.22	0.0373	18.826	0.06	1.623	0.217	0.033	17.938
0.15	64.98	0.46	1.204	0.149	-0.242	13.965	0.64	1.48	0.065	-0.282	15.094
0.2	65.96	0.253	1.158	0.36	-0.137	14.076	0.516	1.492	0.129	-0.274	13.799
0.3	67.07	0.151	1.213	0.526	-0.071	13.749	0.342	1.625	0.26	-0.191	13.357
0.4	67.72	0.108	1.287	0.643	-0.058	14.195	0.261	1.801	0.378	-0.167	13.753
0.5	68.13	0.085	1.351	0.713	-0.048	14.141	0.208	1.923	0.471	-0.138	13.803
0.6	68.4	0.068	1.394	0.764	-0.04	13.723	0.15	1.857	0.579	-0.1	13.623
0.8	68.7	0.047	1.455	0.835	-0.031	13.7	0.114	1.951	0.67	-0.08	13.59
1	68.83	0.033	1.481	0.891	-0.025	13.43	0.093	1.969	0.732	-0.072	13.52
1.5	67.68	0.007	1.466	1.005	-0.019	14.122	0.051	1.857	0.872	-0.054	13.746
2	64	0.001	1.496	1.042	-0.019	13.15	0.06	1.884	0.865	-0.069	13.37
3	56.13	0.017	1.541	1.015	-0.043	13.343	0.087	1.887	0.817	-0.106	13.464
4	51.44	0.021	1.481	1.021	-0.048	13.497	0.073	1.63	0.864	-0.095	13.685
5	48.8	0.046	1.494	0.955	-0.073	13.676	0.091	1.56	0.826	-0.112	13.875
6	47.28	0.058	1.485	0.929	-0.083	13.888	0.104	1.513	0.801	-0.123	14.068
8	45.5	0.078	1.528	0.894	-0.098	14.155	0.111	1.479	0.8	-0.127	14.313
10	44.57	0.055	1.503	0.988	-0.076	14.2	0.086	1.415	0.89	-0.102	14.33
15	43.75	0.033	1.577	1.139	-0.059	14.102	0.062	1.408	1.033	-0.086	14.236

Table S6. Equivalent atomic numbers, G–P fitting parameters for EBF and EABF, respectively, for glass C4.

Energy (MeV)	Z_{eq}	G–P fitting parameters for EBF					G–P fitting parameters for EABF				
		a	b	c	d	X_k	a	b	c	d	X_k
0.015	30.99	-0.214	1.002	1.743	0.1872	11.414	-0.22	1.002	1.746	0.192	11.726
0.02	37.46	0.252	2.078	1.433	-0.336	12.522	0.15	1.159	1.484	-0.163	16.432
0.03	38.18	0.134	2.967	0.903	-0.175	26.956	0.142	1.386	0.912	-0.134	23.699
0.04	38.68	0.122	3.297	0.324	-0.046	21.39	0.127	1.389	0.336	-0.099	23.715
0.05	39.1	-0.209	2.715	0.099	0.0178	12.14	-0.08	1.323	0.11	0.071	9.1865
0.06	39.47	0.958	2.19	0.064	-0.146	16.853	0.707	1.293	0.076	-0.184	14.846
0.08	40.07	0.694	1.601	0.085	-0.237	14.345	0.528	1.293	0.121	-0.215	14.084
0.1	68.09	0.064	1.68	0.508	-0.023	17.859	0.068	1.706	0.492	-0.027	17.499
0.15	69.57	0.404	1.23	0.147	-0.159	14.571	0.468	1.54	0.087	-0.143	19.905
0.2	70.37	0.306	1.156	0.297	-0.171	13.853	0.581	1.509	0.098	-0.29	13.884
0.3	71.28	0.162	1.186	0.502	-0.077	13.669	0.375	1.584	0.225	-0.208	13.328
0.4	71.8	0.118	1.252	0.615	-0.062	14.159	0.283	1.72	0.344	-0.179	13.725
0.5	72.13	0.094	1.311	0.685	-0.052	14.134	0.227	1.833	0.434	-0.148	13.759
0.6	72.34	0.077	1.354	0.734	-0.043	13.699	0.161	1.756	0.549	-0.104	13.592
0.8	72.59	0.054	1.416	0.807	-0.033	13.707	0.124	1.864	0.638	-0.084	13.583
1	72.69	0.04	1.447	0.862	-0.028	13.362	0.102	1.896	0.702	-0.075	13.528
1.5	71.89	0.011	1.436	0.986	-0.02	14.242	0.056	1.808	0.851	-0.055	13.816
2	69.28	0.004	1.459	1.026	-0.02	13.286	0.063	1.819	0.852	-0.069	13.408
3	63.31	0.018	1.493	1.014	-0.044	13.272	0.093	1.797	0.801	-0.111	13.526
4	59.37	0.036	1.503	0.976	-0.06	13.74	0.109	1.744	0.775	-0.126	13.857
5	56.96	0.07	1.61	0.888	-0.089	13.959	0.145	1.853	0.701	-0.159	14.109
6	55.54	0.073	1.616	0.894	-0.09	14.136	0.138	1.762	0.727	-0.152	14.236
8	53.84	0.074	1.707	0.922	-0.094	14.171	0.122	1.719	0.791	-0.138	14.306
10	52.92	0.042	1.683	1.061	-0.065	14.128	0.086	1.621	0.921	-0.107	14.191
15	52.02	0.021	1.769	1.24	-0.051	13.841	0.056	1.586	1.103	-0.087	13.993

Table S7. Equivalent atomic numbers, G–P fitting parameters for EBF and EABF, respectively, for glass C5.

Energy (MeV)	Z_{eq}	G–P fitting parameters for EBF					G–P fitting parameters for EABF				
		a	b	c	d	X_k	a	b	c	d	X_k
0.015	31.66	-0.18	1.003	1.646	0.166	11.499	-0.189	1.003	1.65	0.1725	11.911
0.02	38.38	0.203	2.18	1.559	-0.267	12.688	0.123	1.173	1.604	-0.13	16.796
0.03	39.01	0.128	3.119	0.946	-0.184	28.126	0.134	1.415	0.954	-0.131	24.671
0.04	39.47	0.113	3.455	0.323	-0.043	21.873	0.122	1.413	0.332	-0.086	23.594
0.05	39.86	-0.24	2.822	0.082	0.028	12.091	-0.098	1.339	0.094	0.0848	8.9473
0.06	40.21	1.001	2.256	0.046	-0.146	17.053	0.735	1.303	0.06	-0.186	14.865
0.08	40.8	0.722	1.624	0.065	-0.244	14.366	0.545	1.296	0.106	-0.22	14.068
0.1	69.53	0.088	1.701	0.598	-0.052	17.362	0.093	1.727	0.579	-0.055	17.208
0.15	70.91	0.388	1.237	0.146	-0.136	14.741	0.42	1.557	0.093	-0.105	21.25
0.2	71.67	0.321	1.156	0.279	-0.18	13.79	0.599	1.513	0.089	-0.294	13.908
0.3	72.51	0.164	1.179	0.495	-0.079	13.647	0.385	1.573	0.215	-0.213	13.32
0.4	72.98	0.121	1.242	0.607	-0.064	14.149	0.289	1.697	0.334	-0.182	13.717
0.5	73.28	0.097	1.3	0.677	-0.053	14.131	0.232	1.808	0.424	-0.151	13.747
0.6	73.49	0.079	1.343	0.726	-0.043	13.693	0.165	1.728	0.541	-0.105	13.584
0.8	73.71	0.056	1.405	0.799	-0.033	13.709	0.127	1.84	0.629	-0.085	13.581
1	73.8	0.042	1.438	0.854	-0.028	13.343	0.105	1.876	0.693	-0.076	13.53
1.5	73.07	0.012	1.427	0.981	-0.02	14.275	0.058	1.794	0.845	-0.056	13.835
2	70.72	0.005	1.449	1.021	-0.02	13.322	0.063	1.802	0.849	-0.069	13.418
3	65.24	0.017	1.477	1.015	-0.043	13.281	0.092	1.768	0.803	-0.11	13.531
4	61.48	0.034	1.486	0.981	-0.058	13.74	0.106	1.7	0.781	-0.124	13.863
5	59.16	0.068	1.586	0.897	-0.087	13.966	0.142	1.795	0.709	-0.157	14.123
6	57.75	0.075	1.64	0.888	-0.091	14.187	0.144	1.801	0.713	-0.156	14.273
8	56.08	0.073	1.807	0.927	-0.092	14.178	0.127	1.853	0.781	-0.143	14.287
10	55.16	0.038	1.815	1.081	-0.061	14.09	0.087	1.77	0.926	-0.109	14.117
15	54.23	0.015	1.957	1.275	-0.046	13.767	0.053	1.769	1.125	-0.086	13.892

Table S8. Equivalent atomic numbers, G–P fitting parameters for EBF and EABF, respectively, for glass C6.

Energy (MeV)	Z_{eq}	G–P fitting parameters for EBF					G–P fitting parameters for EABF				
		a	b	c	d	X_k	a	b	c	d	X_k
0.015	33.66	-0.096	1.004	1.369	0.1069	11.741	-0.105	1.004	1.375	0.117	12.441
0.02	41.01	0.071	2.456	1.902	-0.08	13.137	0.0473	1.213	1.928	-0.041	17.782
0.03	41.19	0.114	3.504	1.053	-0.209	31.076	0.1134	1.488	1.061	-0.122	27.125
0.04	41.48	0.093	3.844	0.322	-0.034	23.072	0.11	1.474	0.322	-0.053	23.294
0.05	41.78	-0.309	3.08	0.042	0.053	11.973	-0.148	1.379	0.056	0.118	8.3655
0.06	42.06	1.093	2.413	0.006	-0.147	17.412	0.7915	1.329	0.025	-0.189	14.964
0.08	42.57	0.77	1.672	0.032	-0.248	14.481	0.5853	1.307	0.077	-0.228	14.055
0.1	72.9	0.143	1.749	0.802	-0.116	16.237	0.1499	1.775	0.774	-0.119	16.549
0.15	74.04	0.353	1.254	0.146	-0.084	15.13	0.3129	1.595	0.108	-0.018	24.278
0.2	74.65	0.354	1.159	0.24	-0.196	13.717	0.6189	1.527	0.074	-0.285	14.245
0.3	75.31	0.174	1.166	0.476	-0.085	13.598	0.4097	1.554	0.193	-0.224	13.305
0.4	75.69	0.126	1.224	0.592	-0.066	14.151	0.3056	1.66	0.312	-0.192	13.686
0.5	75.92	0.101	1.278	0.663	-0.054	14.147	0.2452	1.763	0.402	-0.159	13.723
0.6	76.07	0.082	1.319	0.712	-0.044	13.714	0.17	1.672	0.526	-0.107	13.572
0.8	76.25	0.059	1.381	0.786	-0.033	13.704	0.1341	1.791	0.61	-0.087	13.565
1	76.31	0.045	1.415	0.84	-0.028	13.439	0.1107	1.833	0.675	-0.078	13.524
1.5	75.79	0.015	1.409	0.969	-0.021	14.382	0.063	1.769	0.828	-0.058	13.883
2	74.09	0.007	1.427	1.012	-0.02	13.401	0.0652	1.764	0.841	-0.069	13.441
3	69.99	0.016	1.437	1.02	-0.041	13.359	0.0893	1.707	0.808	-0.106	13.536
4	66.94	0.028	1.434	1.002	-0.053	13.734	0.1028	1.617	0.792	-0.12	13.864
5	64.99	0.062	1.518	0.92	-0.082	13.976	0.1339	1.652	0.73	-0.149	14.149
6	63.76	0.069	1.566	0.914	-0.087	14.161	0.1353	1.637	0.739	-0.149	14.299
8	62.29	0.068	1.754	0.957	-0.087	14.142	0.1213	1.708	0.805	-0.139	14.28
10	61.48	0.034	1.836	1.117	-0.059	13.995	0.0805	1.691	0.96	-0.104	14.112
15	60.61	0.006	2.085	1.338	-0.038	13.696	0.0278	2.089	1.238	-0.061	13.722

Table S9. Equivalent atomic numbers, G–P fitting parameters for EBF and EABF, respectively, for glass S1.

**Energy (MeV)**	Z_{eq}	G–P fitting parameters for EBF					G–P fitting parameters for EABF				
		**a**	**b**	**c**	**d**	X_k	**a**	**b**	**c**	**d**	X_k
0.015	20.61	-0.04	1.007	0.706	0.155	6.8667	-0.03	1.007	0.696	0.1388	9.2494
0.02	28.68	0.75	1.009	0.106	-1.017	10.841	0.43	1.006	0.233	-0.488	13.174
0.03	29.68	0.199	1.184	0.405	-0.061	13.262	0.238	1.048	0.416	-0.173	12.309
0.04	30.34	0.221	1.396	0.329	-0.088	15.541	0.186	1.093	0.383	-0.259	25.177
0.05	30.82	0.151	1.406	0.301	-0.108	12.737	0.178	1.122	0.303	-0.097	12.134
0.06	31.2	0.408	1.365	0.293	-0.137	14.325	0.358	1.162	0.277	-0.158	14.605
0.08	31.78	0.334	1.306	0.344	-0.146	14.076	0.305	1.259	0.317	-0.149	14.303
0.1	46.18	0.507	1.288	0.167	-0.246	13.772	0.508	1.272	0.153	-0.272	13.583
0.15	47.93	0.254	1.224	0.366	-0.14	14.064	0.394	1.44	0.215	-0.217	13.918
0.2	48.98	0.171	1.286	0.502	-0.093	14.581	0.325	1.701	0.286	-0.192	13.933
0.3	50.26	0.107	1.401	0.644	-0.051	14.214	0.208	1.913	0.452	-0.118	13.809
0.4	51.02	0.068	1.513	0.776	-0.044	14.137	0.159	2.232	0.574	-0.112	13.868
0.5	51.52	0.047	1.582	0.855	-0.037	14.069	0.114	2.304	0.686	-0.088	13.872
0.6	51.86	0.029	1.614	0.915	-0.026	13.961	0.09	2.337	0.75	-0.075	13.724
0.8	52.24	0.013	1.651	0.977	-0.02	13.946	0.061	2.302	0.839	-0.058	13.623
1	52.37	0.006	1.656	1.01	-0.018	13.363	0.045	2.229	0.888	-0.05	13.511
1.5	50.15	-0.02	1.569	1.121	-0.005	14.285	0.005	1.919	1.032	-0.025	13.657
2	43.54	-0.02	1.592	1.123	-0.006	12.719	0.006	1.85	1.037	-0.027	13.08
3	34.46	-0	1.589	1.062	-0.021	12.503	0.016	1.695	1.001	-0.036	12.708
4	31.14	0.01	1.538	1.022	-0.029	13.005	0.018	1.553	0.995	-0.037	13.972
5	29.62	0.017	1.481	1.004	-0.033	13.279	0.023	1.458	0.984	-0.041	14.129
6	28.72	0.021	1.429	0.994	-0.037	13.242	0.023	1.382	0.983	-0.039	14.303
8	27.81	0.035	1.353	0.965	-0.05	13.558	0.035	1.293	0.959	-0.048	14.031
10	27.37	0.042	1.293	0.955	-0.057	13.942	0.039	1.231	0.958	-0.051	14.298
15	27.04	0.05	1.196	0.961	-0.061	14.409	0.051	1.146	0.951	-0.057	14.694

Table S10. Equivalent atomic numbers, G–P fitting parameters for EBF and EABF, respectively, for glass S2.

Energy (MeV)	Z_{eq}	G–P fitting parameters for EBF					G–P fitting parameters for EABF				
		a	b	c	d	X_k	a	b	c	d	X_k
0.015	26.29	-0.53	1.004	1.61	0.342	6.1687	-0.53	1.004	1.61	0.3421	6.1687
0.02	32.83	0.516	1.527	0.747	-0.71	11.623	0.301	1.08	0.837	-0.342	14.46
0.03	33.83	0.165	2.111	0.664	-0.12	20.381	0.188	1.224	0.674	-0.153	18.23
0.04	34.55	0.168	2.414	0.326	-0.066	18.67	0.154	1.251	0.358	-0.173	24.395
0.05	35.07	-0.04	2.116	0.191	-0.04	12.413	0.039	1.231	0.199	-0.006	10.536
0.06	35.5	0.71	1.818	0.167	-0.142	15.713	0.55	1.233	0.167	-0.172	14.737
0.08	36.18	0.536	1.471	0.199	-0.197	14.227	0.43	1.278	0.207	-0.186	14.181
0.1	59.06	0.456	1.515	0.101	-0.097	15.954	0.441	1.511	0.103	-0.098	15.532
0.15	60.89	0.425	1.196	0.186	-0.237	13.791	0.623	1.451	0.081	-0.302	13.904
0.2	61.93	0.219	1.169	0.406	-0.117	14.186	0.46	1.494	0.163	-0.256	13.783
0.3	63.14	0.142	1.242	0.548	-0.065	13.814	0.311	1.675	0.294	-0.176	13.399
0.4	63.84	0.099	1.323	0.67	-0.053	14.228	0.239	1.883	0.413	-0.155	13.782
0.5	64.3	0.077	1.391	0.742	-0.045	14.149	0.189	2.015	0.508	-0.127	13.846
0.6	64.6	0.059	1.435	0.795	-0.036	13.746	0.139	1.959	0.609	-0.096	13.655
0.8	64.95	0.04	1.495	0.864	-0.028	13.692	0.103	2.039	0.703	-0.077	13.598
1	65.07	0.025	1.517	0.92	-0.023	13.499	0.083	2.045	0.763	-0.068	13.512
1.5	63.68	0.002	1.497	1.025	-0.017	13.994	0.046	1.907	0.892	-0.052	13.674
2	59.29	0.002	1.534	1.043	-0.021	13.104	0.056	1.952	0.879	-0.068	13.337
3	50.61	0.005	1.525	1.053	-0.034	13.027	0.051	1.731	0.913	-0.075	13.29
4	45.88	0.016	1.487	1.029	-0.044	13.395	0.059	1.592	0.898	-0.081	13.581
5	43.41	0.046	1.514	0.945	-0.071	13.609	0.086	1.568	0.831	-0.104	13.801
6	42.01	0.057	1.497	0.921	-0.08	13.82	0.096	1.514	0.811	-0.113	14
8	40.38	0.076	1.512	0.887	-0.094	14.081	0.103	1.46	0.81	-0.117	14.265
10	39.54	0.056	1.467	0.965	-0.075	14.161	0.08	1.383	0.891	-0.094	14.347
15	38.87	0.039	1.493	1.087	-0.061	14.227	0.062	1.351	1.003	-0.08	14.391

Table S11. Equivalent atomic numbers, G–P fitting parameters for EBF and EABF, respectively, for glass S3.

Energy (MeV)	Z_{eq}	G–P fitting parameters for EBF					G–P fitting parameters for EABF				
		a	b	c	d	X_k	a	b	c	d	X_k
0.015	29.34	-0.29	1.001	1.991	0.2405	11.196	-0.29	1.001	1.991	0.241	11.251
0.02	36.04	0.329	1.917	1.232	-0.446	12.259	0.194	1.136	1.295	-0.215	15.856
0.03	36.91	0.142	2.727	0.836	-0.16	25.114	0.155	1.341	0.845	-0.139	22.167
0.04	37.5	0.134	3.055	0.324	-0.052	20.643	0.135	1.351	0.342	-0.119	23.902
0.05	37.97	-0.16	2.553	0.124	0.0022	12.214	-0.05	1.298	0.134	0.05	9.5515
0.06	38.36	0.891	2.09	0.092	-0.145	16.547	0.665	1.277	0.101	-0.181	14.817
0.08	39	0.652	1.567	0.116	-0.227	14.314	0.502	1.289	0.144	-0.207	14.11
0.1	65.31	0.016	1.638	0.329	0.0332	18.848	0.017	1.664	0.321	0.028	18.078
0.15	66.93	0.436	1.215	0.148	-0.206	14.227	0.566	1.506	0.074	-0.222	17.174
0.2	67.82	0.276	1.157	0.333	-0.152	13.98	0.544	1.499	0.116	-0.281	13.836
0.3	68.84	0.156	1.201	0.515	-0.074	13.715	0.356	1.608	0.245	-0.198	13.345
0.4	69.42	0.112	1.272	0.631	-0.06	14.18	0.27	1.766	0.363	-0.172	13.741
0.5	69.79	0.089	1.334	0.701	-0.05	14.138	0.216	1.885	0.455	-0.142	13.784
0.6	70.04	0.072	1.377	0.752	-0.041	13.713	0.155	1.814	0.566	-0.102	13.61
0.8	70.31	0.05	1.439	0.823	-0.031	13.703	0.118	1.914	0.657	-0.082	13.587
1	70.41	0.035	1.467	0.879	-0.026	13.402	0.096	1.939	0.719	-0.073	13.523
1.5	69.45	0.009	1.453	0.997	-0.019	14.173	0.053	1.836	0.863	-0.054	13.776
2	66.31	0.002	1.479	1.035	-0.019	13.211	0.061	1.855	0.859	-0.069	13.387
3	59.4	0.019	1.523	1.011	-0.044	13.344	0.092	1.865	0.802	-0.111	13.504
4	55.09	0.032	1.509	0.987	-0.057	13.659	0.098	1.74	0.8	-0.117	13.795
5	52.58	0.055	1.536	0.931	-0.079	13.793	0.112	1.672	0.777	-0.131	13.974
6	51.11	0.062	1.508	0.925	-0.085	13.965	0.114	1.565	0.782	-0.133	14.129
8	49.37	0.076	1.525	0.91	-0.098	14.159	0.112	1.477	0.809	-0.129	14.336
10	48.43	0.05	1.504	1.021	-0.072	14.185	0.085	1.417	0.907	-0.104	14.301
15	47.58	0.029	1.587	1.182	-0.058	13.981	0.06	1.411	1.064	-0.087	14.144

Table S12. Equivalent atomic numbers, G–P fitting parameters for EBF and EABF, respectively, for glass S4.

Energy (MeV)	Z_{eq}	G–P fitting parameters for EBF					G–P fitting parameters for EABF				
		a	b	c	d	X_k	a	b	c	d	X_k
0.015	31.4	-0.195	1.003	1.683	0.1744	11.466	-0.2	1.003	1.686	0.18	11.84
0.02	38.58	0.193	2.201	1.586	-0.253	12.722	0.117	1.176	1.629	-0.123	16.872
0.03	39.18	0.127	3.15	0.954	-0.186	28.357	0.132	1.421	0.963	-0.13	24.864
0.04	39.61	0.112	3.483	0.323	-0.042	21.962	0.121	1.418	0.331	-0.083	23.572
0.05	39.99	-0.243	2.84	0.08	0.0298	12.083	-0.1	1.342	0.092	0.087	8.9068
0.06	40.33	1.008	2.266	0.043	-0.146	17.085	0.739	1.304	0.058	-0.187	14.868
0.08	40.9	0.726	1.627	0.063	-0.245	14.369	0.547	1.296	0.104	-0.221	14.065
0.1	69.08	0.081	1.694	0.57	-0.043	17.514	0.085	1.72	0.552	-0.047	17.297
0.15	70.47	0.393	1.234	0.146	-0.144	14.686	0.436	1.551	0.091	-0.117	20.811
0.2	71.22	0.316	1.156	0.285	-0.177	13.812	0.593	1.512	0.092	-0.292	13.9
0.3	72.07	0.163	1.181	0.497	-0.078	13.655	0.381	1.577	0.218	-0.211	13.323
0.4	72.56	0.12	1.246	0.61	-0.063	14.152	0.287	1.705	0.338	-0.181	13.72
0.5	72.86	0.096	1.304	0.68	-0.052	14.132	0.23	1.817	0.428	-0.15	13.752
0.6	73.07	0.078	1.347	0.729	-0.043	13.695	0.163	1.738	0.544	-0.105	13.587
0.8	73.29	0.056	1.409	0.802	-0.033	13.709	0.126	1.849	0.633	-0.084	13.581
1	73.39	0.041	1.441	0.857	-0.028	13.35	0.104	1.883	0.697	-0.076	13.529
1.5	72.65	0.012	1.43	0.983	-0.02	14.263	0.057	1.799	0.847	-0.056	13.828
2	70.29	0.005	1.452	1.023	-0.02	13.311	0.063	1.807	0.85	-0.069	13.415
3	64.82	0.018	1.481	1.015	-0.043	13.274	0.092	1.774	0.802	-0.11	13.531
4	61.11	0.035	1.489	0.98	-0.059	13.74	0.107	1.708	0.78	-0.124	13.862
5	58.82	0.068	1.59	0.895	-0.088	13.965	0.142	1.804	0.707	-0.157	14.121
6	57.44	0.076	1.644	0.886	-0.092	14.188	0.144	1.81	0.712	-0.157	14.272
8	55.81	0.073	1.795	0.926	-0.092	14.177	0.126	1.837	0.782	-0.142	14.29
10	54.91	0.039	1.8	1.079	-0.062	14.094	0.086	1.753	0.925	-0.109	14.126
15	53.99	0.015	1.937	1.271	-0.047	13.775	0.054	1.749	1.122	-0.086	13.902

Table S13. Equivalent atomic numbers, G–P fitting parameters for EBF and EABF, respectively, for glass S5.

Energy (MeV)	Z_{eq}	G–P fitting parameters for EBF					G–P fitting parameters for EABF				
		a	b	c	d	X_k	a	b	c	d	X_k
0.015	32.9	-0.13	1.004	1.473	0.129	11.65	-0.136	1.004	1.478	0.1379	12.243
0.02	40.49	0.096	2.403	1.837	-0.116	13.051	0.062	1.205	1.866	-0.058	17.595
0.03	40.74	0.117	3.427	1.032	-0.204	30.487	0.118	1.473	1.04	-0.124	26.636
0.04	41.07	0.097	3.765	0.322	-0.036	22.829	0.112	1.462	0.324	-0.06	23.355
0.05	41.38	-0.29	3.027	0.051	0.048	11.997	-0.138	1.37	0.064	0.1112	8.4851
0.06	41.67	1.084	2.38	0.012	-0.148	17.434	0.787	1.323	0.03	-0.19	14.902
0.08	42.17	0.768	1.664	0.033	-0.254	14.425	0.577	1.302	0.08	-0.228	14.045
0.1	71.63	0.123	1.731	0.726	-0.092	16.654	0.129	1.757	0.702	-0.095	16.793
0.15	72.82	0.366	1.247	0.145	-0.104	14.977	0.353	1.58	0.102	-0.051	23.127
0.2	73.48	0.341	1.155	0.255	-0.193	13.704	0.624	1.519	0.077	-0.3	13.941
0.3	74.2	0.169	1.169	0.485	-0.082	13.617	0.398	1.558	0.201	-0.219	13.309
0.4	74.6	0.124	1.23	0.598	-0.065	14.144	0.298	1.671	0.321	-0.187	13.701
0.5	74.86	0.099	1.286	0.668	-0.053	14.138	0.24	1.78	0.411	-0.156	13.732
0.6	75.02	0.081	1.329	0.717	-0.044	13.702	0.168	1.694	0.532	-0.106	13.576
0.8	75.22	0.058	1.39	0.791	-0.033	13.707	0.131	1.81	0.618	-0.086	13.572
1	75.29	0.044	1.424	0.846	-0.028	13.396	0.108	1.85	0.682	-0.077	13.527
1.5	74.72	0.014	1.416	0.974	-0.021	14.333	0.061	1.778	0.836	-0.057	13.863
2	72.83	0.006	1.435	1.015	-0.02	13.373	0.064	1.777	0.845	-0.069	13.432
3	68.39	0.016	1.451	1.018	-0.041	13.333	0.09	1.727	0.806	-0.107	13.535
4	65.18	0.031	1.453	0.993	-0.055	13.737	0.103	1.636	0.79	-0.121	13.867
5	63.15	0.064	1.542	0.912	-0.084	13.978	0.136	1.689	0.724	-0.151	14.145
6	61.9	0.071	1.588	0.906	-0.089	14.169	0.138	1.686	0.731	-0.151	14.291
8	60.4	0.069	1.786	0.948	-0.088	14.155	0.124	1.777	0.796	-0.141	14.28
10	59.57	0.034	1.87	1.108	-0.058	14.022	0.083	1.772	0.948	-0.107	14.09
15	58.71	0.007	2.134	1.327	-0.039	13.688	0.039	2.036	1.193	-0.073	13.747

Table S14. Equivalent atomic numbers, G–P fitting parameters for EBF and EABF, respectively, for glass S6.

Energy (MeV)	Z_{eq}	G–P fitting parameters for EBF					G–P fitting parameters for EABF				
		a	b	c	d	X_k	a	b	c	d	X_k
0.015	34.62	-0.055	1.005	1.241	0.0795	11.853	-0.066	1.005	1.248	0.0917	12.686
0.02	73.55	0.139	1.003	0.465	-0.082	24.785	0.139	1.003	0.465	-0.081	24.676
0.03	42.39	0.105	3.603	1.133	-0.208	31.016	0.103	1.527	1.14	-0.114	27.118
0.04	42.57	0.089	3.913	0.391	-0.039	23.597	0.106	1.502	0.392	-0.043	22.459
0.05	42.79	-0.253	3.149	0.086	0.0245	12.477	-0.108	1.41	0.099	0.0834	9.1944
0.06	43.02	0.947	2.482	0.036	-0.135	15.791	0.678	1.36	0.055	-0.157	15.774
0.08	43.46	0.776	1.69	0.03	-0.236	14.606	0.604	1.318	0.072	-0.228	14.079
0.1	74.19	0.158	1.768	0.883	-0.136	16.224	0.166	1.794	0.848	-0.137	16.254
0.15	75.21	0.34	1.277	0.178	-0.075	15.431	0.311	1.615	0.138	-0.015	24.786
0.2	75.73	0.365	1.165	0.228	-0.195	13.779	0.599	1.536	0.075	-0.258	14.73
0.3	76.31	0.178	1.163	0.468	-0.087	13.581	0.42	1.55	0.185	-0.228	13.301
0.4	76.63	0.128	1.218	0.588	-0.067	14.157	0.312	1.649	0.304	-0.195	13.673
0.5	76.83	0.102	1.271	0.658	-0.054	14.156	0.25	1.749	0.394	-0.161	13.714
0.6	76.97	0.083	1.312	0.708	-0.044	13.725	0.172	1.654	0.521	-0.107	13.568
0.8	77.12	0.06	1.373	0.782	-0.033	13.702	0.136	1.774	0.604	-0.088	13.56
1	77.18	0.046	1.408	0.836	-0.028	13.475	0.113	1.818	0.669	-0.079	13.522
1.5	76.75	0.015	1.403	0.965	-0.021	14.424	0.065	1.761	0.821	-0.059	13.9
2	75.34	0.008	1.42	1.006	-0.02	13.419	0.068	1.758	0.832	-0.071	13.45
3	71.95	0.015	1.422	1.021	-0.04	13.389	0.088	1.683	0.811	-0.105	13.538
4	69.33	0.025	1.409	1.015	-0.05	13.729	0.102	1.59	0.795	-0.119	13.859
5	67.65	0.058	1.48	0.934	-0.079	13.965	0.131	1.614	0.738	-0.147	14.146
6	66.58	0.065	1.518	0.928	-0.084	14.209	0.134	1.591	0.745	-0.148	14.297
8	65.28	0.066	1.69	0.969	-0.086	14.133	0.12	1.625	0.812	-0.138	14.284
10	64.55	0.033	1.774	1.128	-0.059	13.968	0.078	1.577	0.975	-0.101	14.144
15	63.76	0.005	2.006	1.357	-0.037	13.709	0.009	2.174	1.311	-0.041	13.683

Table S15. Effective removal cross-sections for fast neutrons, Σ_R (cm^{-1}), for all C1–C6 glasses.

Glass code	Element	Σ_R/ρ (cm^2/g)	Fraction by weight %	Partial density (g/cm^3)	Σ_R (cm^{-1})
C1	O	0.0405	0.4648	1.4428	0.0584
	Ca	0.0243	0.1164	0.3614	0.0088
	B	0.0575	0.1759	0.5460	0.0314
	Bi	0.0103	0.2429	0.7538	0.0078
			**Total 0.1064**		
C2	O	0.0405	0.3650	1.4105	0.0571
	Ca	0.0243	0.1143	0.4417	0.0107
	B	0.0575	0.1233	0.4765	0.0274
	Bi	0.0103	0.3973	1.5353	0.0158
			**Total 0.1110**		
C3	O	0.0405	0.2846	1.3711	0.0555
	Ca	0.0243	0.0548	0.2642	0.0064
	B	0.0575	0.0887	0.4276	0.0246
	Bi	0.0103	0.5718	2.7552	0.0284
			**Total 0.1149**		
C4	O	0.0405	0.2160	1.2529	0.0507
	Ca	0.0243	0.0541	0.3138	0.0076
	B	0.0575	0.0526	0.3048	0.0175
	Bi	0.0103	0.6773	3.9275	0.0405
			**Total 0.1163**		
C5	O	0.0405	0.2094	1.2676	0.0513
	Ca	0.0243	0.0291	0.1764	0.0043
	B	0.0575	0.0524	0.3172	0.0182
	Bi	0.0103	0.7091	4.2927	0.0442
			**Total 0.1180**		
C6	O	0.0405	0.168139	1.1758	0.0476
	Ca	0.0243	0.015042	0.1052	0.0026
	B	0.0575	0.032461	0.2270	0.0131
	Bi	0.0103	0.784356	5.4850	0.0565
			**Total 0.1198**		

Table S16. Effective removal cross-sections for fast neutrons, Σ_R (cm^{-1}), for all S1–S6 glasses.

Glass code	Element	Σ_R/ρ (cm^2/g)	Fraction by weight %	Partial density (g/cm^3)	Σ_R (cm^{-1})
S1	O	0.0405	0.3854	1.3766	0.0558
	Sr	0.0160	0.2638	0.9424	0.0151
	B	0.0575	0.1411	0.5038	0.0290
	Bi	0.0103	0.2097	0.7492	0.0077
			**Total 0.1076**		
S2	O	0.0405	0.3220	1.4228	0.0576
	Sr	0.0160	0.0980	0.4427	0.0071
	B	0.0575	0.1128	0.5098	0.0293
	Bi	0.0103	0.4673	2.1116	0.0217
			**Total 0.1157**		
S3	O	0.0405	0.2603	1.3900	0.0563
	Sr	0.0160	0.0509	0.2617	0.0042
	B	0.0575	0.0817	0.4197	0.0241
	Bi	0.0103	0.6071	3.1207	0.0321
			**Total 0.1167**		
S4	O	0.0405	0.2116	1.2752	0.0516
	Sr	0.0160	0.0414	0.2494	0.0040
	B	0.0575	0.0562	0.3385	0.0195
	Bi	0.0103	0.6909	4.1639	0.0429
			**Total 0.1180**		
S5	O	0.0405	0.1782	1.1898	0.0482
	Sr	0.0160	0.0349	0.2327	0.0037
	B	0.0575	0.0387	0.2584	0.0149
	Bi	0.0103	0.7482	4.9951	0.0514
			**Total 0.1182**		
S6	O	0.0405	0.1441	1.0939	0.0443
	Sr	0.0160	0.0282	0.2140	0.0034
	B	0.0575	0.0209	0.1584	0.0091
	Bi	0.0103	0.8068	6.1237	0.0631
			**Total 0.1199**		

Table S17. Coherent scattering cross-section (σ_{cs}), incoherent scattering cross-section (σ_{ics}), absorption cross-section (σ_A), and total cross-section (σ_T) of all (i) C1–C6 and (ii) S1–S6 glasses for thermal neutrons attenuation. All derived vlaues are in cm^{-1} units.

(i) C1–C6 glasses

Glass code	σ_{cs}	σ_{ics}	σ_A	σ_T
C1	0.372320836	0.052014595	23.31988889	23.74422432
C2	0.377438108	0.045522246	20.3560067	20.77896705
C3	0.386244416	0.040783038	18.26411142	18.69113887
C4	0.361844198	0.028104991	12.52273588	12.91268507
C5	0.384875769	0.03030552	13.55084797	13.96602926
C6	0.380909001	0.021735561	9.69630217	10.09894673

(ii) S1–S6 glasses

Glass code	σ_{cs}	σ_{ics}	σ_A	σ_T
S1	0.378341783	0.048143765	21.52756033	21.95404588
S2	0.406639459	0.048534109	21.77641243	22.231586
S3	0.389148279	0.039957207	17.9294571	18.35856259
S4	0.390110323	0.032285366	14.45984187	14.88223756
S5	0.381917834	0.024714239	11.039053	11.44568507
S6	0.375888227	0.015263495	6.767510604	7.158662326

Table S18. Coherent scattering cross-section (σ_{cs}, barn), incoherent scattering cross-section (σ_{ics}, barn), absorption cross-section (σ_A, barn), and total cross-section (σ_T, barn) of B, Bi, Ca, Sr, and O elements for thermal neutrons.

Element	σ_{cs}	σ_{ics}	σ_A	σ_T
B	3.54	1.7	767	772.24
Bi	9.148	0.0084	0.0338	9.1902
Ca	2.78	0.05	0.43	3.26
Sr	6.19	0.06	1.28	7.53
O	4.232	0.0008	0.00019	4.23299
